# Nanoceria as an Acid–Base Catalyst for Interfacial Hydrolysis of Sulfonylureas: A Combined ^31^P MAS NMR and DFT Study

**DOI:** 10.1021/acs.inorgchem.6c04712

**Published:** 2026-10-23

**Authors:** Jan Čundrle, Martin Št’astný, Tereza Dušková, Dmytro Bavol, Daniela Popelková, Jakub Ederer, Nusrat Jahan Rifat, Ye Xu, Jiří Henych

**Affiliations:** † Institute of Inorganic Chemistry of the Czech Academy of Sciences, 250 68 Husinec-Řež, Czechia; ‡ Faculty of Environment, Jan Evangelista Purkyne University in Ústí nad Labem, Pasteurova 3632/15, 400 96 Ústí nad Labem, Czechia; § Faculty of Science, 562998 Jan Evangelista Purkyne University in Ústí nad Labem, Pasteurova 3632/15, 400 96 Ústí nad Labem, Czechia; ∥ Cain Department of Chemical Engineering, Louisiana State University, Baton Rouge, Louisiana 70803, United States; ⊥ Charles University Prague, Faculty of Science, Imaging Methods Core Facility BIOCEV, Vestec 252 50, Czech Republic

## Abstract

Understanding coordination-driven bond activation at metal oxide interfaces is essential for predicting the chemical stability of complex organic molecules. Here, we demonstrate that nanoceria efficiently drives the spontaneous, dark hydrolysis of sulfonylureas (amidosulfuron, metsulfuron-methyl, and nicosulfuron) via surface acid–base chemistry under ambient conditions. By combining liquid chromatography–mass spectrometry (LC-HRMS) with density functional theory (DFT) calculations, we show that the degradation proceeds through urea N–C bond cleavage. While the experimental identification of the degradation products confirms the overall hydrolytic pathway, DFT calculations are used to provide unique insights into the specific mechanism, proposing that the reaction is initiated by lattice-oxygen nucleophilic attack leading preferentially to sulfonyl-side N–C bond cleavage. Crucially, solid-state ^31^P MAS NMR spectroscopy using a trimethylphosphine oxide probe explicitly mapped the distribution and strength of surface Lewis and Brønsted acidic sites (oxygen vacancies and bridging hydroxyls). This provides a molecular-level link between ceria defect structures and their unique interfacial reactivity, highlighting the potential of tailored ceria surfaces to drive spontaneous chemical transformations without external energy inputs.

## Graphical abstract

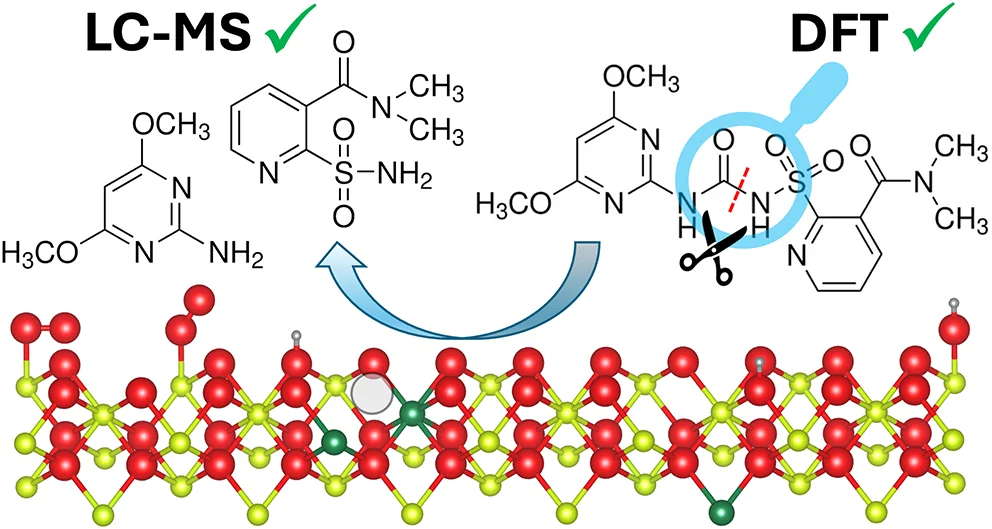

## Introduction

Sulfonylurea herbicides (SUHs) are widely used due to their high efficacy at low doses (10–40 g/ha), low mammalian toxicity, and broad-spectrum weed control in crops such as wheat, rice, and soybeans.^1^ Their action is based on the inhibition of acetolactate synthase (ALS), which is essential for the biosynthesis of branched-chain amino acids in plants.^2^ SUHs are often found in surface and groundwater as a result of runoff and leaching.^2,3^ Even low concentrations can harm nontarget plants and affect ecosystems.^3^ Although SUHs are often considered to have low general toxicity, several studies have reported adverse effects on aquatic organisms. Sublethal concentrations were shown to impair neurological, metabolic, and developmental functions in fish, indicating potential ecological risks to aquatic environments.^4^

In the environment, SUHs are degraded mainly by chemical hydrolysis and microbial degradation.^1,2^ Hydrolysis is faster under acidic conditions and at higher temperatures, and involves cleavage of the sulfonylurea bridge.^2^ At neutral to alkaline pH, the anionic form of the SUHs predominates, which reduces the rate of hydrolysis.^1,3^ Microbial degradation is efficient and environmentally safe and proceeds via hydrolysis, redox reactions, or decarboxylation.^1,3^ In soils, both abiotic and biotic degradation occur depending on pH and microbial presence.^1^ Photolysis is usually negligible due to low light absorption and limited UV exposure.^2,3^

Removal of SUHs from water can be achieved by a variety of physical, biological and chemical methods. Biochar^5,6^ and polymeric materials containing graphene oxide,^7^ as well as oxygen-defective graphdiyne,^8^ have been investigated for simple adsorption. Other techniques have included electroflotation,^9^ oxidative degradation using HClO^10^ or ClO_2_,^11^ as well as photolytic ozonation^12^ and Fenton or Fenton-like reactions.^13^ Photocatalytic degradation has also been extensively investigated using TiO_2_-based materials,^14,15^ other metal oxides^16^ and g-C_3_N_4_.^17^ In addition, a comprehensive review^3^ has summarized the use of diverse microbial species for the bioremediation of SUH-contaminated water and soil.

Reactive adsorption and catalytic degradation of SUH have been less studied. Nanoscale zerovalent iron^18^ supported on biochar has been used for SUH removal from soil by combined adsorption and degradation, and MnO_2_ nanoimmobilized laccase^19^ has enabled catalytic degradation of nicosulfuron. Degradation of bensulfuron-methyl has also been reported on mineral surfaces such as kaolinite, silica gel, alumina, H^+^-bentonite, montmorillonite K10 and humic acids.^20^ Metal oxide–carbon catalysts Co_3_O_4_@C^21^ and Fe_2_O_3_/Fe_3_O_4_@C^22^ have been used to activate peroxymonosulfate for the degradation of bensulfuron-methyl. Over the past decade, nanoceria has emerged as a prominent abiotic catalyst and nanozyme capable of driving the hydrolytic cleavage of various organically and biologically relevant esters.^23^ This exceptional mimetic activity is heavily governed by surface chemistry and defect engineering. Specifically, vacancy-engineered CeO_2_ nanoparticles^24^ have been demonstrated to create enzymatic “hotspots” that significantly accelerate the degradation of complex molecules via advanced surface-bound intermediates and cooperative multistep pathways. The efficiency of these transformations is strongly dictated by the Ce^3+^/Ce^4+^ ratios, surface states, and the specific architecture of accessible active sites.^25^

Herein, we demonstrate for the first time—through a combination of experimental techniques and first-principles density functional theory (DFT) calculations—that nanoceria efficiently catalyzes the spontaneous, dark hydrolysis of sulfonylurea linkages at the solid–liquid interface. Notably, this process occurs under ambient conditions without requiring light illumination or external chemical reagents, driven by the inherent acid–base and redox properties of CeO_2_. To establish a molecular-level understanding of the active centers, solid-state ^31^P MAS NMR spectroscopy using a TMPO probe was employed to map the specific surface sites, explicitly distinguishing Lewis and Brønsted acidic sites, oxygen vacancies, and surface hydroxyl groups. While LC-MS kinetic studies established the structural stability of the molecules under various temperature, pH, and coadsorption conditions, DFT calculations revealed that the reaction proceeds via a lattice-oxygen-mediated nucleophilic attack leading to sulfonyl-side N–C bond cleavage. By evaluating differently synthesized nanoceria samples, this work highlights how tailored metal oxide defect chemistry and surface properties dictate the coordination and abiotic transformation pathways of complex organic ligands.

## Materials and Methods

### Sample Synthesis

To evaluate the robustness of sulfonylurea transformation across different ceria surface states, three nanoceria samples with distinct synthesis histories and surface properties were selected based on our previous study.^26^ Three nanoceria samples were synthesized using different precipitation methods reported previously^26,27^ and denoted according to the synthesis route as CeAMM (ammonia-based), CePER (peroxide-assisted), and CeUREA (urea-based). In short, the CeAMM sample was prepared by precipitation of an aqueous cerium­(III) nitrate hexahydrate solution with ammonium hydroxide at 60 °C under CO_2_-free conditions and aging in solution for 4 h. The CePER sample was synthesized by precipitating an aqueous cerium­(III) nitrate hexahydrate solution with NaOH (to reach pH > 11), followed by the addition of 30% hydrogen peroxide and refluxing at 100 °C for 24 h. The CeUREA sample was obtained by homogeneous precipitation of an aqueous cerium nitrate­(III) solution with urea under heating at ∼90 °C for 6 h, followed by calcination of the dried precipitate at 500 °C for 2 h. CePER and CeAMM samples were purified by distilled water using a dialysis membrane and air-dried. See experimental details in the Supporting Information (SI).

### Characterization Methods

The crystalline phase structure of the materials was investigated by powder X-ray diffraction (PXRD) using a PANalytical X′Pert PRO instrument equipped with Cu Kα radiation (λ = 1.5418 Å). Raman spectroscopy was performed with a DXR Raman confocal microscope (Thermo Fisher Scientific) using a 532 nm laser as the excitation source. The morphological features and particle architecture were analyzed by transmission electron microscopy (TEM) using a FEI Talos F200X microscope (Thermo Fisher Scientific) and scanning electron microscope (SEM) using a FEI Nova NanoSEM 450 with a Circular Backscatter (CBS) detector in high vacuum mode and at an acceleration voltage of 5 kV. To assess textural properties, nitrogen adsorption–desorption measurements were carried out on a NOVAtouch LX2 analyzer (Quantachrome Instruments, Anton Paar, Austria). Prior to analysis, the samples were degassed at 25 °C for 20 h. Specific surface area was determined using the Brunauer–Emmett–Teller (BET) method, and pore size distribution was determined by applying nonlinear density functional theory (NLDFT). Direct potentiometric acid–base titration with HCl or NaOH was used to quantify the number of surface hydroxyl groups by the method described previously^28^ with some modifications. 300 mg of sample was placed in 50 mL of 0.1 M NaCl; 3 mL of 0.1 M HCl solution (in 0.1 M NaCl) was added, and titrated with 0.1 M NaOH (in 0.1 M NaCl) to pH approximately 11 using an automated titrator (794 Basic Titrino, Metrohm, Switzerland). The solution was constantly stirred and bubbled with N_2_ during the titration. The titration rate was set to 100 μL/min. For acid (reverse) titration, the sample was first basified with NaOH and titrated with HCl (to pH 2.5–3) under identical experimental conditions.

### NMR Spectroscopy

The samples for ^31^P solid-state magic angle spin nuclear magnetic resonance (MAS NMR) analysis were prepared by activating of 0.20 g of CeO_2_ or SiO_2_ (used as a reference) in a round-bottom flask at 100 °C for 2 h under vacuum (10^–1^ Pa), followed by the addition of 3 mL of CH_2_Cl_2_ solution containing 0.1 M trimethyl phosphine oxide (TMPO), which was used as a probe molecule for acid–base sites in ceria, under argon atmosphere. The mixture was sonicated in an ultrasonic bath for 1 h to reach equilibrium, and the solvent was subsequently evacuated under vacuum.^29^ The spectra were recorded at room temperature using a JEOL 400 MHz JNM-ECZ400R/M1NMR spectrometer equipped with a 3.2 mm High Resolution 400 MHz AutoMAS probe. Solid NH_4_H_2_PO_4_ was used as an external standard (δ_P_ = 1 ppm).^30^ Powder samples were placed in a 3.2 mm zirconium dioxide rotor (volume 49 μL). In the single-pulse experiment, a 90° pulse width of 2.4 μs was applied. The cross-polarization (CP) experiment amplifies the signal from phosphorus nuclei in spatial contact with protons in the sample, as CP is driven by the ^1^H–^31^P-dipole–dipole interaction. The technique allows the detection of surface sites and reaction products.^31^ The MAS frequency was set to 10.0 kHz for all experiments. The spectra were deconvoluted in OriginPro 2020b using Lorentzian curve-fitting.

### Catalytic SUH Hydrolysis

The kinetics of SUH adsorption and catalytic hydrolysis were monitored by an analytical procedure developed previously^26,27^ with some modifications. Briefly, 70 mg of the powdered sample was weighed into a 4 mL glass reagent vial and dispersed in 3.9 mL dH_2_O by bath sonication for 10 min. The reaction was initiated by adding 0.1 mL of a stock solution of the selected SUH (400 mg/L in acetonitrile). The vial was placed into a thermo-shaker (1000 rpm, 4 h, 25 ± 0.5 °C), and at selected times, 0.2 mL of the suspension was aliquoted, diluted with 0.8 mL dH_2_O in a 2 mL Eppendorf tube, vortexed (3 s), centrifuged (18,000 rpm, 2 min), and the supernatants were analyzed immediately by HPLC-DAD. Additional LC-MS analysis was performed to identify the degradation products. See further details in SI.

### Blank Experiments

To assess the stability of SUHs, blank experiments without the catalyst in pure deionized water and in an acidic environment mimicking the pH obtained in the CePER aqueous suspension (pH ∼ 3) were conducted. The pH of an acidic aqueous solution was adjusted using a diluted solution of formic acid, which was also employed as a mobile phase modifier (buffer) in subsequent HPLC analysis.

### Effect of Reaction Conditions on Hydrolysis

The effect of reaction conditions (i.e., temperature, pH, ionic strength) on the kinetics of herbicide hydrolysis was studied using the CePER sample. All procedures followed the identical kinetic setup, but with only 10 mg of catalyst to prevent too rapid hydrolysis under given conditions. Temperature-dependent experiments were performed at 5, 15, 25, 35, and 45 °C using a thermo-shaker. Conventional buffers were avoided due to strong inhibitory interactions with the ceria surface (confirmed by preliminary tests, data not shown), likely caused by buffer adsorption on active sites. Instead, pH was adjusted by the addition of 0 to 100 μL of 0.1 M NaOH solution into the reaction mixture, with the pH monitored by a calibrated microelectrode. To evaluate ionic strength and ion-specific effects, sodium or chloride salts were added to the reaction mixture to achieve a 1 mM concentration of the studied cation/anion.

### Theoretical Calculations

Periodic DFT calculations were performed using the optB86b van der Waals functional^32^ within the Vienna Ab initio Simulation Package (VASP, version 5.4.4)^33^ using the optB86b van der Waals (vdW) functional^34^ to account for possible vdW contributions to adsorption. The potentials due to the core electrons were described using the projector-augmented wave method,^35,36^ and the Kohn–Sham valence states (including Ce­(4f5s5p5d6s); S­(3s3p); C, N, and O­(2s2p); and H­(1s)) were expanded in a plane wave basis set with a cutoff energy of 400 eV. The DFT+U approach of Dudarev et al.^37^ was commonly employed to rectify the delocalization of Ce 4f electrons in ceria resulting from the self-interaction error of semilocal functionals. As was done previously,^38,39^ a *U*_eff_ value of 2 eV was used based on better agreements between GGA+*U* predictions at *U*_eff_ of 2∼3 eV and available experimental measurements of chemical reaction energies involving ceria bulk and surfaces, although *U*_eff_ = 2 eV does not fully counteract the erroneous delocalization of Ce 4f electrons upon Ce^4+^ reduction to Ce^3+^. The bulk lattice constant of ceria calculated with optB86b-vdW at *U*_eff_ = 2 eV was 5.452 Å,^39^ in good agreement with the experimental value of 5.41 Å.^40^

The (111) facet of CeO_2_ was modeled as a (3 × 3) slab consisting of 9 atomic layers (i.e., three O–Ce–O trilayers), with periodic images of the slab separated by ca. 16 Å of vacuum in the z direction and with dipole decoupling applied in the same direction.^41^ A Γ-centered Monkhorst–Pack 2 × 2 × 1 *k*-point grid was used to sample the surface Brillouin zone^42^ with Gaussian smearing of the electronic states at a width of 0.01 eV. The top three atomic layers in a slab and any adsorbate thereon were fully relaxed, while the remainder of the slab was held fixed at the bulk coordinates. Geometry optimization was performed until the maximum residual force fell below 0.03 eV/Å in each relaxed ionic degree of freedom. Isolated molecules were calculated and optimized in a (18 Å)^3^ simulation cell with dipole decoupling applied in all directions, until the maximum residual force dropped below 0.01 eV/Å in each relaxed degree of freedom.

The transition states (TSs) of elementary reaction steps were determined using the climbing-image nudged elastic band method^43,44^ and dimer method,^45^ until the maximum residual force converged to 0.01 eV/Å or less in all relaxed degrees of freedom in a TS. The reaction energy, Δ*E*, and the activation energy, *E*_a_, of an elementary step were evaluated as Δ*E* = *E*_FS_ – *E*_IS_ and *E*_a_ = *E*_TS_ – *E*_IS_, respectively, where *E*_IS_, *E*_TS_, and *E*_FS_ are the total energies of the initial, transition, and final states, respectively. Each TS was verified to possess a vibrational mode with a negative curvature in the reaction coordinate under consideration.

To capture aqueous interfacial effects, the implicit nonlinear solvation model of Plaisance and co-workers,^46^ VASPsol++, was used to calculate the solvation energy (Δ*G*_sol_) with the default parameters for water. Energy quantities dependent on Δ*G*_sol_ are referred to below as *G* instead of *E*.

## Results and Discussion

### Phase and Structural Analysis

All three synthesized samples correspond to the standard pattern (JCPDS card No. 34–0394)^47^ for ceria with a cubic fluorite-type structure (Figure 1). The CeAMM and CePER samples show low intensity and significant broadening of diffraction lines due to their nanocrystalline nature. On the contrary, the CeUREA sample exhibits significantly sharper and more intense diffraction lines, indicating a higher degree of crystallinity and larger crystallite domains, due to the calcination at 500 °C. The approximate crystallite sizes estimated using the Scherrer equation were 5.4, 6.8, and 9.4 nm for CePER, CeAMM, and CeUREA, respectively (Table 1).

**Figure 1:**
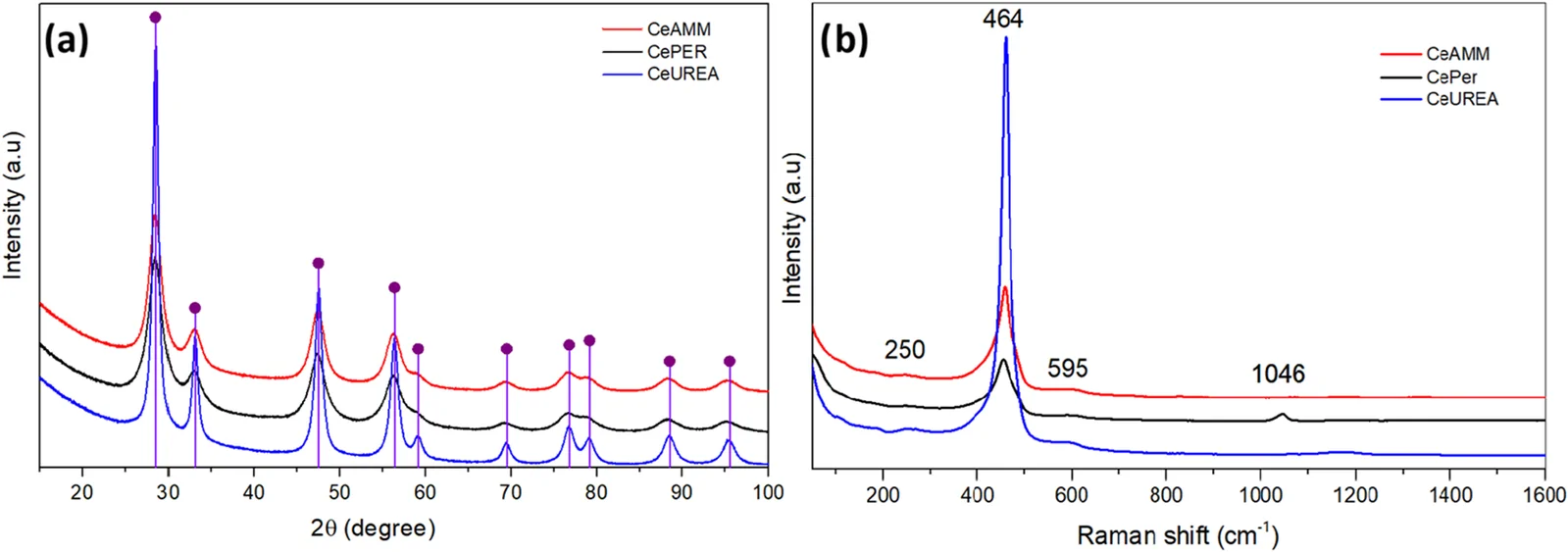
(a) X-ray diffractograms and (b) Raman spectra of prepared ceria samples.

**Table 1:** Total Pore Volume, Specific Surface Area, and Mean Pore Diameter of Prepared Ceria Samples Calculated by the BET Method; Average Crystallite Size Determined from PXRD Data; Concentration of Surface Hydroxyl Groups (qOH) Obtained by Acid and Base Titration

Sample	CeAMM	CePER	CeUREA
Specific surface area (m^2^/g)	199	221	87
Total pore volume (cm^3^/g)	0.333	0.137	0.134
Average pore diameter (nm)	9.9	2.6	12.7
Average crystallite size, XRD (nm)	6.8	5.4	9.4
qOH/base titration (mmol/g)	0.401	0.628	0.069
qOH/acid titration (mmol/g)	0.196	0.317	0.228

The Raman spectra of all three samples (Figure 1b) exhibit a prominent band at ∼464 cm^–1^, corresponding to the triply degenerate F_2_g symmetric stretching mode of O^2–^ anions surrounding Ce^4+^ cations in the CeO_8_ octahedra.^48^ The redshift and asymmetric broadening of this band are related to particle size and the presence of structural defects in the crystal lattice.^49^ Specifically, the F_2_g band is centered at 463 cm^–1^ for CeUREA, 460 cm^–1^ for CeAMM, and 457 cm^–1^ for CePER, indicating that CePER likely has the smallest crystallites and/or the highest concentrations of structural defects. Frenkel-type oxygen defects^50^ at ∼595 cm^–1^ were observed in all samples. A very weak band around 250 cm^–1^ is either the second-order transverse acoustic (2TA) mode of CeO_2_, or is related to surface termination of crystalline (111) planes.^49^ Additionally, the band at 1046 cm^–1^ is due to residual nitrates originating from the Ce precursor used in synthesis.^50^

### Electron Microscopy

The morphology and particle size of the synthesized CeO_2_ materials are significantly influenced by the synthesis conditions, as observed by SEM and TEM, shown in Figures S1–S4 and 2. The CeAMM sample (Figure S1) consists of irregular microagglomerates. At higher magnifications (Figure S1d), these microparticles are seen to be composed of needle-like primary particles. In contrast, the CePER sample (Figure S2) exhibits angular, plate-like microaggregates formed by much smaller primary particles, indicating a heterogeneous microstructure. The CeUREA sample (Figure S3) is formed by disordered platelet-like particles of varying size. Such a morphology typically correlates with a lower specific surface area, which was confirmed by nitrogen physisorption (see below).

**Figure 2:**
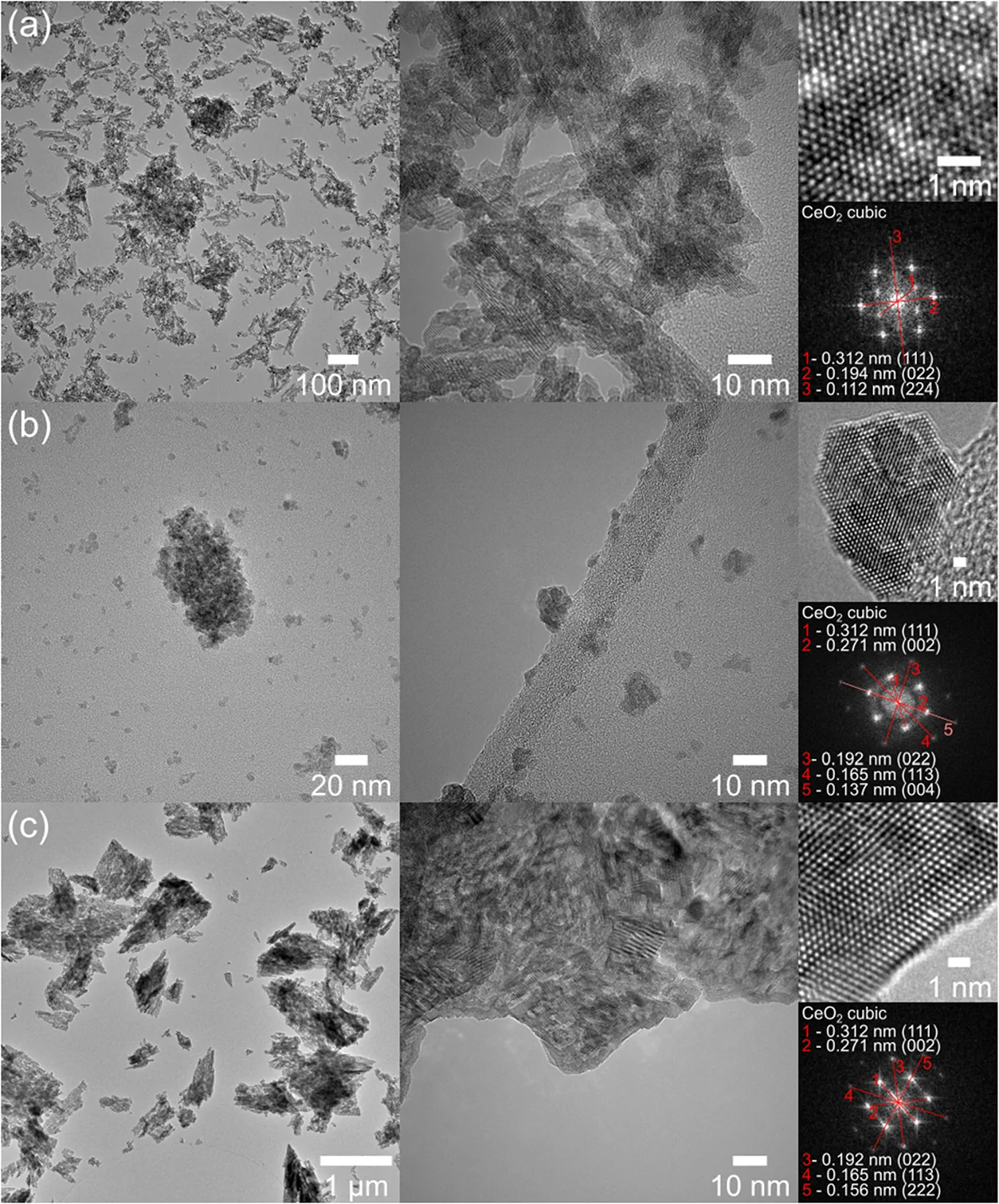
TEM images of the samples (a) CeAMM, (b) CePER, and (c) CeUREA.

TEM analysis (Figure 2) revealed pronounced morphological differences in nanostructure among the CeO_2_ samples, despite their identical cubic fluorite structure (*Fm*3̅*m*), as confirmed by FFT. The CeAMM sample (Figure 2a) is composed of loosely assembled, rod-like aggregates formed by small, faceted crystallites. These structures are relatively open and less densely packed, indicating anisotropic growth and limited aggregation. The CePER sample (Figure 2b) shows densely aggregated, irregular domains consisting of very fine, angular nanoparticles. The crystallites are similar in size to those in CeAMM but are more tightly packed, with less defined morphology and random orientation. In contrast, CeUREA forms large microplates composed of larger and more rounded primary particles. These structures exhibit broader size distribution and frequent contrast variations, suggesting a polycrystalline character and heterogeneous orientation. EDS mapping (Figure S4) confirmed a homogeneous distribution of Ce and O across all samples. Minor traces of K and N were occasionally detected, likely originating from synthesis residues, while Cu and C signals were attributed to the TEM support grid.

### Texture and Porosity

Nitrogen adsorption–desorption isotherms of all samples (Figure S5a) correspond to Type IV isotherm with a distinct hysteresis loop, typical of mesoporous materials. CeAMM exhibits an H3-type hysteresis loop, indicating slit-shaped pores formed by the aggregation of nanoneedles, while CeUREA shows an H4-type loop of narrow slit-like pores often associated with microporous solids.^51^ CePER has a mixed H2/H4-type hysteresis, suggesting the presence of ink-bottle pores and a significant contribution of microporosity. CePER possesses the highest specific surface area (Table 1) along with the smallest average pore diameter, indicating a highly microporous structure.^51^ CeAMM, on the other hand, exhibits the largest total pore volume and the widest pores, resulting in an open mesoporous network of interparticle pores, consistent with the H3-type loop.^51^ As consistent with larger particles shown by TEM and XRD, CeUREA has the lowest surface area and a pore diameter similar to CeAMM, which may reflect partial pore collapse during calcination. This interpretation is further corroborated by NLDFT-derived pore size distributions (Figure S5b), which reveal a dominant peak in the micropore region for CePER, confirming its fine porosity. CeAMM exhibits a broader distribution in the mesoporous range, while CeUREA shows a flatter and less defined profile with limited pore volume. It should be noted that the observed pore size distribution primarily reflects the interparticle voids formed within the dried agglomerates, rather than intraparticle porosity, which explains why the average pore diameter can naturally exceed the primary crystallite size derived from XRD.

The concentration of acidic surface hydroxyl groups (Table 1) was determined from the titration and TOTH curves using the procedure described previously.^28^ Here we also performed a reverse titration (i.e., alkalization of the sample followed by titration with acid), which is commonly used for carbon materials^52,53^ rather than for metal oxides, due to the much easier reversible protonation of oxygen-containing surface groups. However, this method can quantify OH groups with a more basic character than the direct titration procedure so we can more precisely quantify the surface hydroxyl groups on the prepared samples.

Acid–base titration revealed significant differences in the concentration of surface OH groups, indicating that the samples contain varying amounts of hydroxyls with distinct protonation or deprotonation abilities. This is consistent with the fact that the surface of metal oxides (hydroxides) contains various types of OH groups, as well as surface-bound water.^54^ It should be noted that while prolonged exposure to low or high pH can induce partial dissolution of ceria, such processes are kinetically negligible during the brief time frame of the titration, owing to the exceptionally low solubility product of nanoceria (log *K*_sp_ = −59.3) demonstrated by Plakhova et al.^55^ However, potential leaching of synthesis residues, alongside the presence of surface-bound carboxylate and carbonate species,^56^ may partially influence the titration curves. While these coexisting surface species preclude a strictly accurate determination of the absolute number of OH groups, they do not compromise the overall comparative trends observed across the studied samples.

As shown in Table 1, the CePER sample contains noticeably more OH groups in both acid and base titration than the other two samples. It is also worth mentioning that, when alkalizing the CePER sample to an initial pH > 11, twice the amount of NaOH was needed compared with the other two samples, indicating its much higher buffering capacity. It suggests that CePER has an extensively hydroxylated surface, which may be related to its very small particle size and abundance of defect sites,^26^ and may have a significant effect on its surface chemical properties.

### ^31^P MAS NMR

In ^31^P MAS NMR spectroscopy, the chemical shift of TMPO interacting with solid acid–base catalysts is known to be sensitive to the nature and strength of acid sites.^57–61^ The signals at approximately 44–46 ppm are primarily indicative of physisorbed TMPO, whereas signals at higher chemical shifts (typically >50 ppm) can be associated with TMPO interacting with Lewis acid sites (LAS) of varying strength or Brønsted acid sites (BAS), at which TMPO undergoes protonation (53–86 ppm).^59,60^ Note that the TMPO and its analogues are more sensitive for identifying BAS than LAS of different acid strength.^61^ The presence of water on the surface can also affect the signal for LAS, as H_2_O molecules displace TMPO from its position.^60^ The interpretation of the data is based on comparisons with SiO_2_ as a reference and the available literature on related systems, such as zeolites,^62^ silica nanoparticles,^63^ and other metal oxides.^64–67^

The reference ^31^P MAS NMR spectrum of TMPO adsorbed on SiO_2_ (Figure S6a), which contains only silanol groups (Si–OH) as weak acid sites,^58^ shows one distinct signal at 43.96 ppm that is attributed to physisorbed TMPO. The position of the signal is consistent with previous work,^68^ indicating weak interactions of TMPO with the SiO_2_ surface. Nevertheless, a slightly different signal shift for physisorbed TMPO may be expected for nanoceria due to the variations in electronic environment, surface composition, and possible weak interactions with hydroxyl or defect sites.

In addition, several types of OH groups exist on the surface of nanoceria, such as singly coordinated (terminal) and doubly/multicoordinated (bridging) hydroxyl groups.^54^ The latter are strongly influenced by multiple Ce^4+^ cations, which decrease the electron density around oxygen and weaken the bond with hydrogen, thereby leading to an acidic character. In contrast, singly coordinated hydroxyl groups exhibit significantly weaker acidity and are more prone to dissociate as OH^–^ ions, indicating a predominantly basic nature.^69^ TMPO serves as a suitable basic probe molecule, which can be protonated by BAS, specifically by bridging OH groups.^58^ In contrast to BAS on nanoceria, LAS has been observed to abstract electron pairs from the reactant, thereby initiating certain chemical reactions.^70^ Possible interactions of TMPO with the different surface sites on nanoceria are presented in Figure 3.

**Figure 3:**
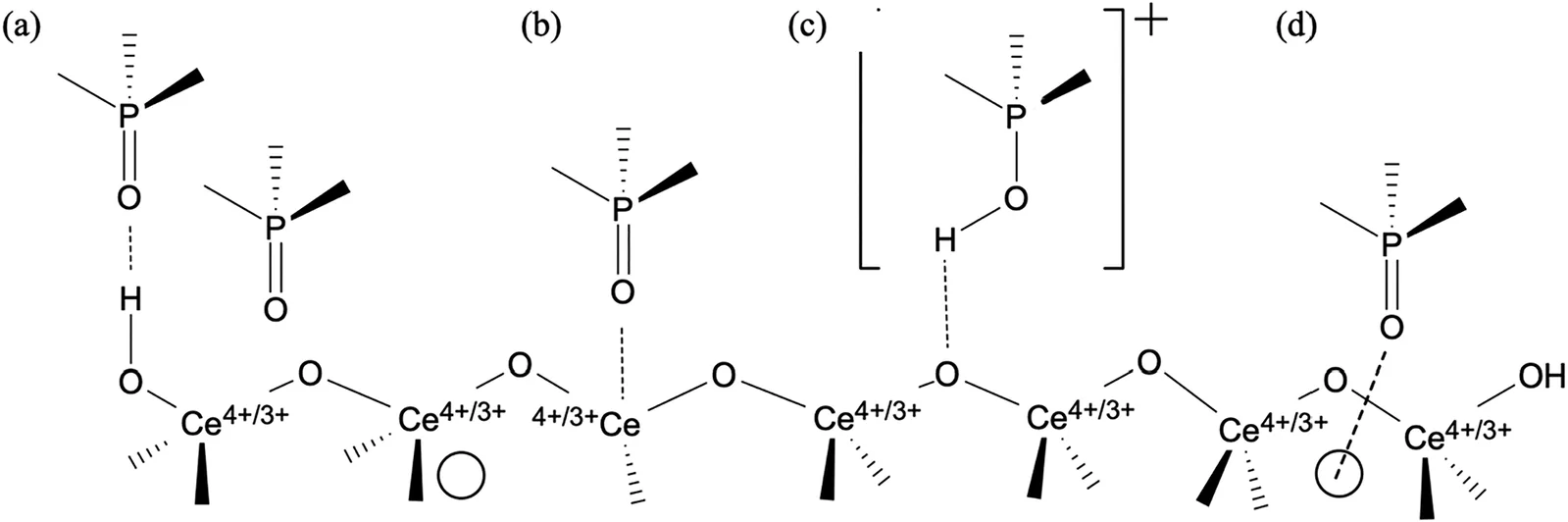
Possible interactions of TMPO with different acid sites on ceria surface: (a) physisorbed and weakly interacting TMPO, (b) Lewis acid site (LAS), (c) Brønsted acid site (BAS)/bridging hydroxyl group, and (d) oxygen vacancy (V_O_). Scheme adapted from ref^60^ available under a CC-BY 4.0 license. Copyright 2021 D. S. Zasukhin, I. A. Kostyukov, I. A. Kasyanov, and Y. G. Kolyagin.

TMPO adsorbed onto CeUREA (Figure 4a) exhibits signals at 41.43, 43.71, and 56.03 ppm, respectively. The dominant 41.43 ppm peak is often ascribed to the excess (bulk-like/crystalline) TMPO molecules^57,65^ with weak surface interactions, rather than protonated TMPOH^+^,^63^ consistent with the sample’s low surface area and negligible microporosity. The signal at 43.71 ppm was assigned to physisorbed TMPO (Figure 3a), based on a comparison with the reference SiO_2_ spectrum and previous work.^57,68^ This suggests that TMPO molecules interact mostly weakly with CeUREA, without substantial acid–base interactions.^63^ The ∼56 ppm signal is tentatively assigned to thermally stable BAS (acidic hydroxyls), despite possible overlap with strong LAS.

**Figure 4:**
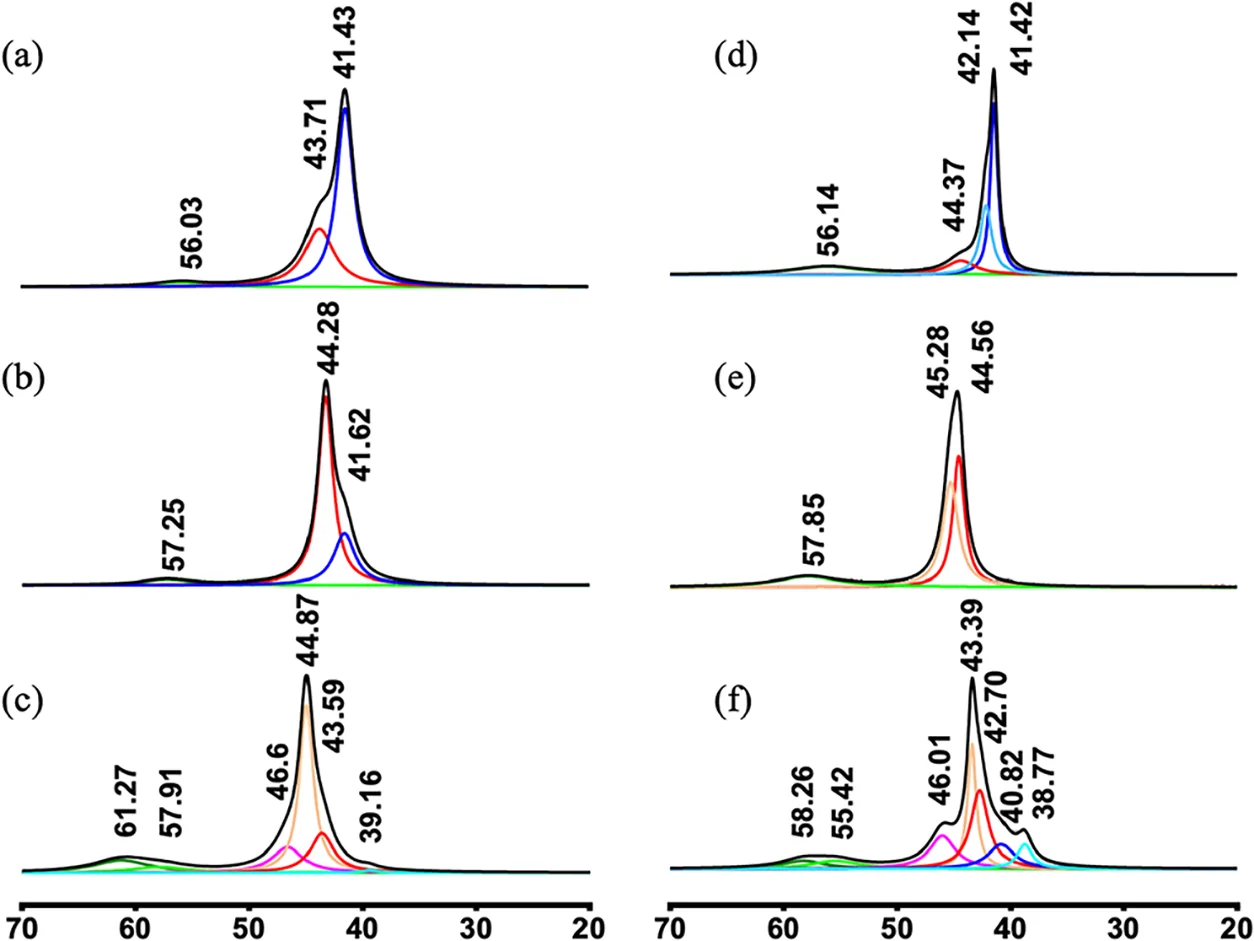
Deconvoluted ^31^P MAS NMR spectra of TMPO adsorbed on CeUREA, CeAMM, and CePER: (a–c) single pulse (left) and (d–f) CP MAS (right) spectra.

Cerium valence influences acid site formation. In nanoceria, oxygen vacancies (V_O_) generated during synthesis or under low oxygen pressure increase the Ce^3+^ fraction to maintain electroneutrality.^71^ However, unlike CeAMM and CePER, CeUREA was calcined at 500 °C in air, which can lead to a decrease in surface area and the surface concentration of Ce^3+^ along with a reduction in defect densities due to oxidation.^72^ Consistently, compared to the other two samples, CeUREA exhibits a lower density of accessible acid sites.

The CeAMM sample (Figure 4b) shows a similar set of signals to CeUREA albeit with significantly different relative intensities. Notably, the intensity of the bulk TMPO signal at ∼41.6 ppm is significantly lower than in CeUREA (∼41.4 ppm), while the signals corresponding to physisorbed TMPO (∼44.3 ppm) and BAS (∼57.3 ppm) are increased. This suggests that CeAMM provides a more favorable environment for TMPO dispersion due to its higher density of accessible active sites. Unlike CeAMM, where TMPO distributes across the surface, the limited number of interactive sites on CeUREA forces the probe molecule to precipitate out as bulk crystals once the solvent is removed. Moreover, although the intensities of the signals at 56.03 ppm for CeUREA and 57.25 ppm for CeAMM are low, their better definition in the latter may indicate a higher abundance of surface hydroxyls in the acidic bridging configuration compared to CeUREA.

The spectrum of CePER (Figure 4c) is notably more complex, with a number of signals at 61.27, 57.91, 46.6, 44.87, 43.59, and 39.16 ppm. Compared to the other two samples, CePER shows a more heterogeneous distribution of active sites for TMPO adsorption, likely due to its surface and structure characteristics.^27^ Concretely, the signals at 61.27 and 57.91 ppm indicate strong acidity attributed to BAS enhanced by surface defects and a high Ce^3+^/Ce^4+^ ratio. The signal at 61.27 ppm is significantly more downfield compared to CeUREA and CeAMM, suggesting stronger BAS. In addition, the signals around 46–47 ppm, can be attributed to LAS of lower strength, while the upfield signals (∼43.6, 39.2 ppm) are due to weak interactions between TMPO and terminal surface hydroxyl groups. In conclusion, the CePER sample has a more complex distribution of acid sites, showing multiple types of interactions with TMPO compared to other investigated samples. Given the inherent complexity and potential overlap of ^31^P TMPO resonances on ceria surfaces, these individual signal assignments should be considered tentative. Nevertheless, the presence of surface defects and oxygen vacancies is independently supported by our Raman data and is consistent with Ce^3+^/Ce^4+^ redox states previously^26^ quantified for similarly synthesized samples via XPS.

### ^31^P MAS NMR of Calcined Samples

To investigate the effect of calcination temperature and to confirm the signal assignment, CeAMM and CePER were treated at 500 °C (as CeUREA^27^) to obtain the samples CeAMM500 and CePER500. In CeAMM500 (Figure S6b), a signal around 45.35 ppm appeared, while the 41.62 ppm signal associated with bulk TMPO disappeared after calcination. In CePER500 (Figure S6c), calcination led to an increase in the intensity of the ∼46 ppm signal, as well as a downfield shift of other signals. This suggests that high-temperature treatment alters the surface composition, in particular enhancing the signal of weak LAS (at 45.8 ppm), and affecting the observed interaction with hydroxyl groups.^59^ Although both CeAMM and CePER undergo structural changes upon calcination (such as dehydration),^73^ the nature and distinct initial surface characteristics of the acid sites associated with their synthesis are preserved.

### ^31^P CP MAS NMR

A cross-polarization (CP) MAS experiment (Figure 3, **right**) was performed on all calcined samples. The CP MAS does not show new spectral features, but it selectively enhances the relative visibility of ^31^P nuclei in close spatial contact with protons, thereby improving the resolution of overlapping signals.^31^ The dominant signal at ∼41–45 ppm, attributed to the weak TMPO interactions (i.e., physisorption or bulk retention), appears sharper and partially resolved into individual contributions. Thus, the signals in the ∼50–61 ppm region, assigned to TMPO interaction with BAS, are more clearly visible. The experiment highlights differences in the composition of the acidic sites across the samples, which may be linked to their distinct adsorption behavior and surface reactivity.

### Adsorption and Hydrolysis of SUHs on Nanoceria

The adsorption (Figure 5) and ceria-catalyzed hydrolysis (Figure 6) of the three SU herbicides amidosulfuron (AS), metsulfuron-methyl (MSM), and nicosulfuron (NS) in aqueous solution were investigated. The structure of the herbicides with a typical structural motif formed by a sulfonyl and ureylene group, but with various heterocyclic substituents, can be seen in the inset graphics in Figure 6.

**Figure 5:**
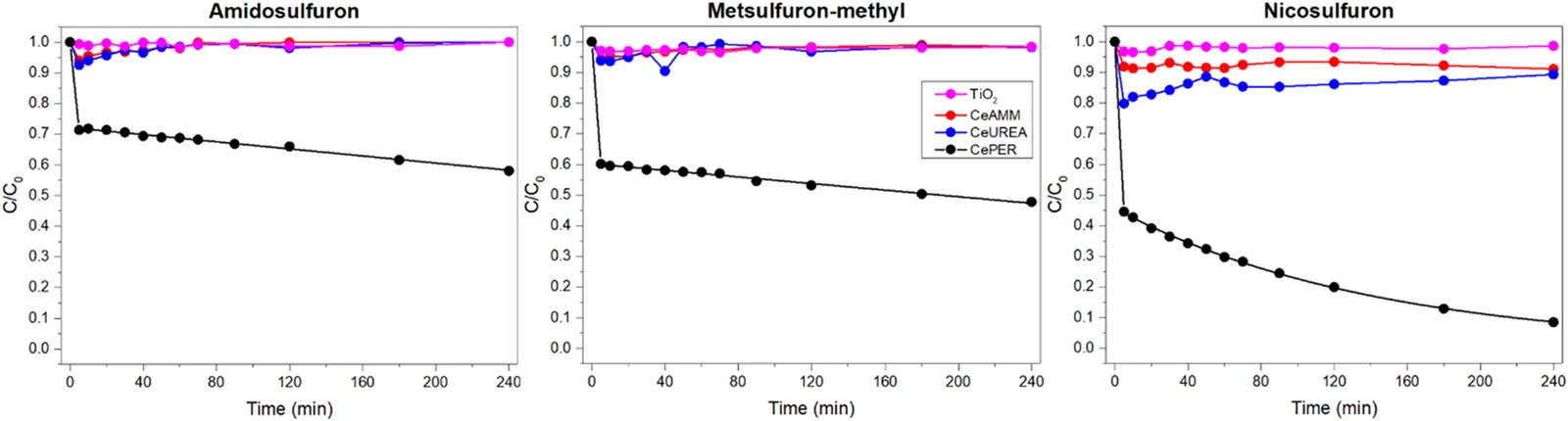
Kinetics of SUH adsorption on prepared ceria samples and a reference TiO_2_ sample.

**Figure 6:**
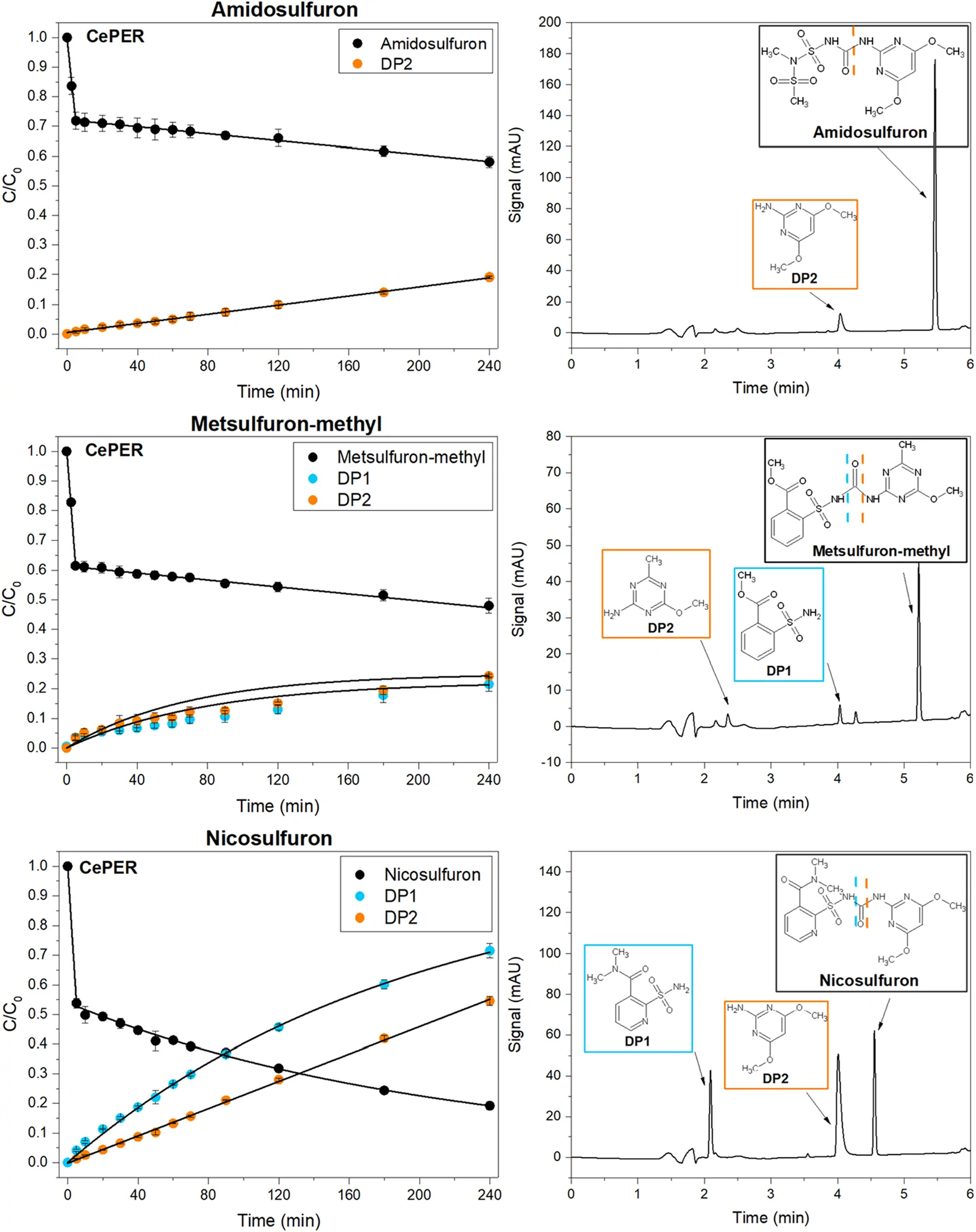
Degradation kinetics of SUH accompanied by the formation of degradation products using the CePER sample (left) and the corresponding HPLC-DAD chromatograms (right) showing the structures of the identified products.

Of the tested materials, only the CePER sample showed a rapid and sustained decrease in the concentration of all three herbicides in water over time (Figure 5). In contrast, both CeAMM and CeUREA showed low adsorption, similar to a reference TiO_2_ sample, synthesized via homogeneous precipitation with urea.^74^ Importantly, the significant SUH adsorption on the CePER sample was accompanied by the gradual formation of two degradation products (Figure 6). This suggests that nanoceria can significantly accelerate the hydrolysis of sulfonylureas on its surface, similarly to other compounds such as phosphate esters,^75^ sulfonamides,^27^ or other esters,^39^ but this was demonstrated by only one sample, which apparently has some unique features compared to the other materials tested. No significant product formation was observed for the CeUREA and CeAMM samples.

As evident, for the tested herbicides, two main degradation products (denoted as DP1, DP2) were identified by comparison of the retention times with reference standards and further confirmed by LC-MS analysis (see below). In general, DP1 is a sulfonamide, while DP2 is an amine residue. They can likely be formed by cleaving either the sulfonyl-side N–C (N_*S*_–C) or the C–N bond, creating an R–NH–C=O species (and an R–NH_2_), which presumably reacts rapidly with water to yield another R–NH_2_ and CO_2_. Because the final stable degradation products identified by LC-MS can result from initial cleavage at either site, the experimental data alone cannot unambiguously distinguish which specific N–C bond undergoes the primary scission. Therefore, based strictly on the experimental observations, only the overall hydrolytic pathway can be confirmed. In addition, only DP2 was detected for AS, which may be due to poor separation in the column and detection of DP1 in our HPLC-DAD system. The formation of other minor degradation products in chromatograms will be discussed together with a tentative cleavage mechanism further below.

Furthermore, long-term 24 h experiments (Figure 7) revealed continuous degradation, indicating the catalytic rather than stoichiometric mechanism of the reaction. Continuous product formation on CePER suggests efficient product turnover without active site poisoning, which was further verified by the recycling experiment (Figure S7), showing that the catalytic performance was maintained over three consecutive cycles. This is in contrast to the degradation of phosphate esters, such as warfare agents^76^ or pesticides,^75^ which often leads to the accumulation of hydrolysis products on the catalyst surface and its deactivation.

**Figure 7:**
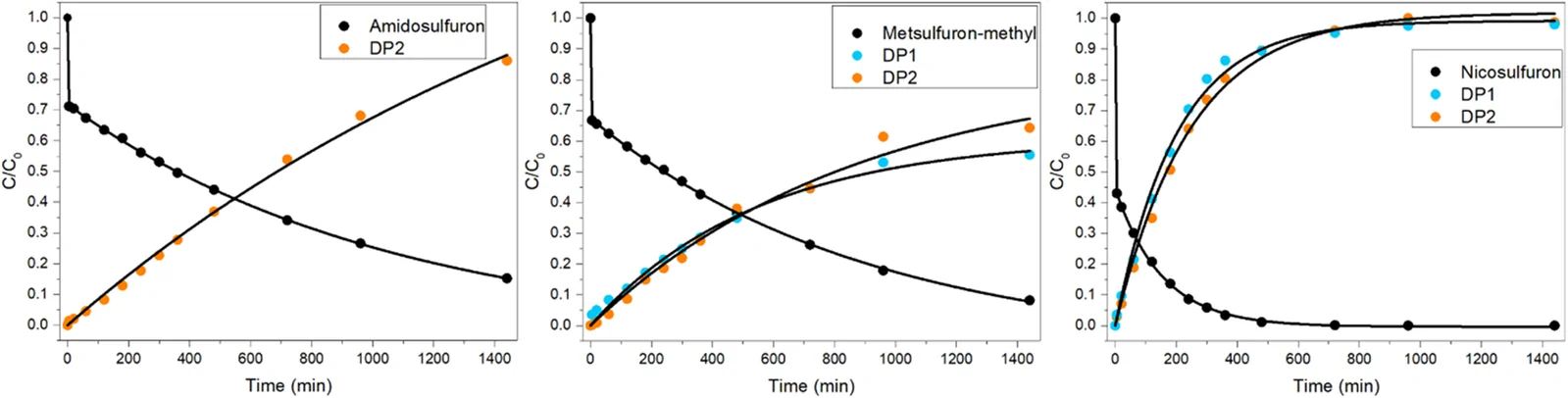
Long-term (24 h) kinetics of the degradation of sulfonylurea herbicides catalyzed by the CePER sample.

NS was degraded most rapidly among tested herbicides, with near-complete removal within 300 min and a sharp, synchronous rise in both degradation products. Evidently, DP1 and DP2 account for most of the NS degradation, as their amounts are stoichiometric with respect to the original herbicide removed from the solution. This also applies to AS, as inferred from the slope of the kinetic curve of DP2, although its formation (or desorption) occurs with a delay. In contrast, this is not the case for MSM, where the production of both DP1 and DP2 slows down significantly over time, likely failing to reach the stoichiometric amount of MSM removed from the solution. This suggests that another degradation pathway may be involved, or that part of the MSM is only adsorbed without undergoing reaction. Interestingly, DP1 is formed at a higher rate than DP2 in the initial stages of NS degradation, showing a noticeable time lag in the accumulation of the latter (Figure 6). This may suggest a stepwise pathway for NS, which is further rationalized in the DFT section below. Furthermore, while NS yields stoichiometric amounts of both products within 24 h, the mass balance for MSM remains incomplete (Figure 7), implying that a portion of MSM remains tightly adsorbed or undergoes alternative degradation pathways.

The kinetics of simultaneous degradation of all three SUHs in one reaction mixture (Figure S8) show similar kinetics as in individual experiments (rate decrease in the order NS > MSM ∼ AS).

These findings underscore that the superior catalytic performance of CePER arises from a complex interplay of multiple surface features rather than a single structural descriptor—a complexity that prevents an unambiguous definition of a governing descriptor and, thereby, precludes an accurate quantification of the exact number of active sites. While its higher BET surface area provides more accessible sites, our comparative approach reveals that materials with similar particle sizes, polyhedral morphologies, oxygen vacancies, and Ce^3+^/Ce^4+^ states (as characterized in detail our previous work^26^) remain catalytically inactive under identical conditions. This clearly demonstrates that materials sharing similar physical and chemical traits can exhibit vastly different catalytic activity. Consequently, nanoceria-mediated hydrolysis is inherently governed by a cooperative combination of several parameters, such as exposed facets, lattice strain, defects, and the specific architecture of various acidic/basic sites in a proper spatial arrangement.^24,25,77,78^ Here, the unique active-site architecture of CePER, qualitatively mapped by TMPO NMR, likely plays a decisive role in driving the interfacial transformation.

### Acid Hydrolysis Versus Ceria-Catalyzed Reaction

Acid hydrolysis is a key route for environmental SUH degradation.^1^ As reported previously,^27^ dispersing the CePER sample in deionized water decreases the pH of the suspension to ∼3, which may drive SUH degradation. Therefore, to evaluate the stability of the herbicides under applied conditions, blank experiments without catalysts were performed. The compounds were incubated in (a) pure deionized water and (b) an acidic environment adjusted to pH ∼ 3 using diluted formic acid. In deionized water, all herbicides remained stable over the period of 4 h (Figure S9), while in the acidic medium, slight hydrolysis was observed (Figure S10). The order of stability, from highest to lowest, was as follows: NS > AS > MSM. After 4 h at pH ∼ 3, approximately 10% of MSM was degraded.

Comparative kinetic studies of ceria-catalyzed and uncatalyzed acidic hydrolysis (Figure S11) reveal that SUH initially rapidly adsorbs onto CePER (0–5 min) before undergoing gradual decomposition and product desorption. Notably, at matching pH without catalyst, the degradation slope for AS and MSM after 5 min is similar to the catalyzed reaction, indicating that acid hydrolysis plays an important role. However, despite partial degradation of the parent herbicides under acidic conditions (∼10% for AS and MSM, and ∼5% for NS after 4 h), only traces of DP1 and DP2 were detected without ceria (Figure S11). To tentatively quantify the individual contribution of adsorption, acid-induced and ceria-catalyzed reaction and their dynamic changes, the parent compound kinetic curves were combined with the product mass balances across multiple time scales, as summarized in Table S4. Overall, these findings highlight that acid-induced hydrolysis does contribute to SUH degradation, but the pathways and efficiency differ substantially from those observed for ceria catalysis.

### Effect of Environmental Conditions

The effects of pH, temperature, and ionic composition on NS reaction kinetics were investigated to assess CePER performance under different reaction conditions. The NS degradation was found to be strongly pH-dependent (Figure S12). The acidic conditions (initial pH ≈ 3.5) increase the degradation rate and yield of DP1/DP2, while increasing pH slows down the reaction rate. At pH ∼ 6.3, the NS degradation was almost completely suppressed. These results suggest that ceria induced low pH correlates with high catalytic activity of ceria. Throughout the experiment, pH was monitored to confirm that the system remained within the intended pH range after equilibration (Figure S13).

The NS degradation also exhibits strong temperature dependence (Figure S14). At lower temperatures (5 °C), rapid adsorption occurs, with almost no subsequent conversion; moreover, a gradual desorption of the herbicide was observed over time. In contrast, at elevated temperatures (35, 45 °C), rapid decomposition is observed, resulting in near-complete conversion within the first 60–90 min. The formation of both DPs follows the same trend, i.e., it is faster with a higher yield with increasing temperature, suggesting that temperature can promote initial bond cleavage as well as subsequent product desorption from the CeO_2_ surface.

The influence of selected inorganic ions (Na^+^, Mg^2+^, Mn^2+^, Fe^2+^, Cl^–^, NO_3_^–^, PO_4_^3–^, SO_4_^2–^ 1 mM each) on the reaction rate was also examined (Figures S15–S16). All reactions proceeded at comparable pH values (3.45–3.75) excluding pH as a factor in the observed differences (see Table S3). As shown, phosphate ions moderately inhibited the reaction, while sulfates nearly stopped it. This strong anion-dependent inhibition is in good agreement with previous findings on ceria nanozymes, where coordinating anions (such as phosphates or sulfates) compete for identical surface defect locations, effectively blocking the open active sites from substrate coordination.^78^ Among cations, Fe^2+^ had the strongest effect, likely due to specific surface interactions. The degree of NS degradation correlated with the formation of hydrolysis products, indicating that ionic inhibition impacts both substrate conversion and product formation. Carbonate, bicarbonate, and humic acid were excluded from the interference tests because at pH < 6, carbonate species convert to dissolved CO_2_, while humic acid undergo protonation and precipitation, rendering their competitive effects negligible.

### LC-MS Analysis

High-resolution liquid chromatography–mass spectrometry (LC–HRMS) was employed to identify both major and minor degradation products of CePER-catalyzed SUH hydrolysis. The parent herbicides were identified in both positive and negative modes: AS at *t*_R_ = 5.52 min (*m*/*z* 370 [M + H]^+^, 368 [M–H]^−^, (Figure S17)), MSM at *t*_R_ = 5.33 min (*m*/*z* 382 [M + H]^+^, 380 [M–H]^−^, (Figure S18)), and NS at *t*_R_ = 4.70 min (*m*/*z* 411 [M + H]^+^, 409 [M–H]^−^, (Figure S19)). Their isotopic envelopes were virtually identical in both modes, fully consistent with theoretical predictions.

Time-resolved chromatograms (Figure S20) demonstrate the progressive decrease of the parent herbicide (NS) peaks in the CePER-catalyzed reaction, accompanied by the simultaneous formation of three DPs (Figure S21). Both major DPs, DP1 - *N*,*N*-dimethyl-2-sulfamoylnicotinamide (C_8_H_11_N_3_O_3_S; *t*_R_ = 2.17 min, *m*/*z* 230 [M + H]^+^), and DP2, 2-amino-4,6-dimethoxypyrimidine (ADMP; C_6_H_9_N_3_O_2_; *t*_R_ = 3.23 min, *m*/*z* 156 [M + H]^+^) were confirmed by comparison with the reference standard (Figures S22 and S23). In this study, ADMP product was consistently detected during the degradation of both AS and NS. For AS, ADMP has long been recognized as the dominant hydrolytic product, released through scission of the sulfonylurea linkage under aqueous conditions.^79^ Its occurrence in the NS pathway, confirmed in this study, aligns with recent reports of ADMP formation during NS hydrolysis.^80^ The parallel outcome reflects the shared pyrimidine fragment within their molecular frameworks. A third, minor DP appeared at *t*_R_ = 3.75 min (*m*/*z* 347 [M + H]^+^) and was assigned to 2-[1-(4,6-dimethoxypyrimidin-2-yl)­ureido]-*N*,*N*-dimethylnicotinamide **(**C_15_H_18_N_6_O_4_). This DP may form under acidic hydrolysis conditions, where C–S bond cleavage of the sulfonylurea bridge occurs with loss of SO_2_.^80^ Although detected only at low intensity, its presence indicates that besides the dominant C–N cleavage route leading to ADMP, alternative acid-driven pathways also contribute to NS degradation on CePER.

Time-resolved chromatograms (Figure S24) again show degradation of AS on CePER with the simultaneous formation of three DPs. The major DP, [(4,6-dimethoxypyrimidin-2-yl)­carbamoyl]­sulfamic acid (C_7_H_10_N_4_O_6_S; BCS-AW41401), *t*_R_ = 3.06 min with *m*/*z* 277 [M–H]^−^ (Figure S24) has been reported in regulatory processing studies of AS under pasteurization conditions.^81^ Similar sulfamic acid derivatives have also been observed during direct photolysis of structurally related sulfonylureas (e.g., foramsulfuron^82,83^ ) in sterile aqueous buffer, indicating that this pathway can proceed abiotically as well. The second degradation product (DP2), 2-amino-4,6-dimethoxypyrimidine (ADMP; C_6_H_9_N_3_O_2_, *t*_R_ = 3.23 min, *m*/*z* 156.0770 [M + H]^+^), was likewise verified against the reference standard (Figure S23). As already noted for AS, ADMP forms through cleavage of the sulfonylurea bridge and reflects the conserved pyrimidine moiety of both herbicides. A third, minor DP was assigned to 4,6-dimethoxypyrimidin-2-ylurea (C_7_H_10_N_4_O_3_), detected at *t*_R_ = 4.11 min with *m*/*z* 199.0830 [M + H]^+^; (Figure S26). This product was detected only as a minor peak, which has been, however, previously reported alongside ADMP as a characteristic hydrolytic product.^79^

For MSM (Figure S27), three DPs were detected. The first major DP, methyl 2-(aminosulfonyl)­benzoate (C_8_H_9_NO_4_S; *t*_R_ = 4.33 min; Figure S28), was confirmed with an authentic standard (Figure S29). In positive ion mode, the expected [M + H]^+^ precursor at *m*/*z* 216 was not detected; instead, the dominant ion appeared at *m*/*z* 199, consistent with in-source loss of NH_3_. Loss of ammonia is a characteristic and frequently observed fragmentation pathway of sulfonamide-containing compounds under electrospray ionization (SI).^84,85^ The second major DP, 2-amino-4-methoxy-6-methyl-1,3,5-triazine (C_5_H_8_N_4_O; *t*_R_ = 2.11 min, *m*/*z* 141 [M + H]^+^ Figure S30), was likewise verified with standard (Figure S31). Both principal degradation products were confirmed to originate from hydrolytic cleavage of the sulfonylurea bridge, consistent with the DFT-based predictions.^86^ A third, minor DP (C_13_H_13_N_5_O_6_S; *t*_R_ = 4.12 min, *m*/*z* 368 [M + H]^+^ Figure S32), was tentatively assigned to MSM without a −CH_3_ substituent. Such O-demethylated species on the aryl ring (forming metsulfuron) or on the triazine ring (forming bis-O-demethylmetsulfuron-methyl) have been previously reported under acidic hydrolytic conditions.^87^ Figure 8 shows the structures of the main ceria-catalyzed hydrolytic products identified by LC-MS.

**Figure 8:**
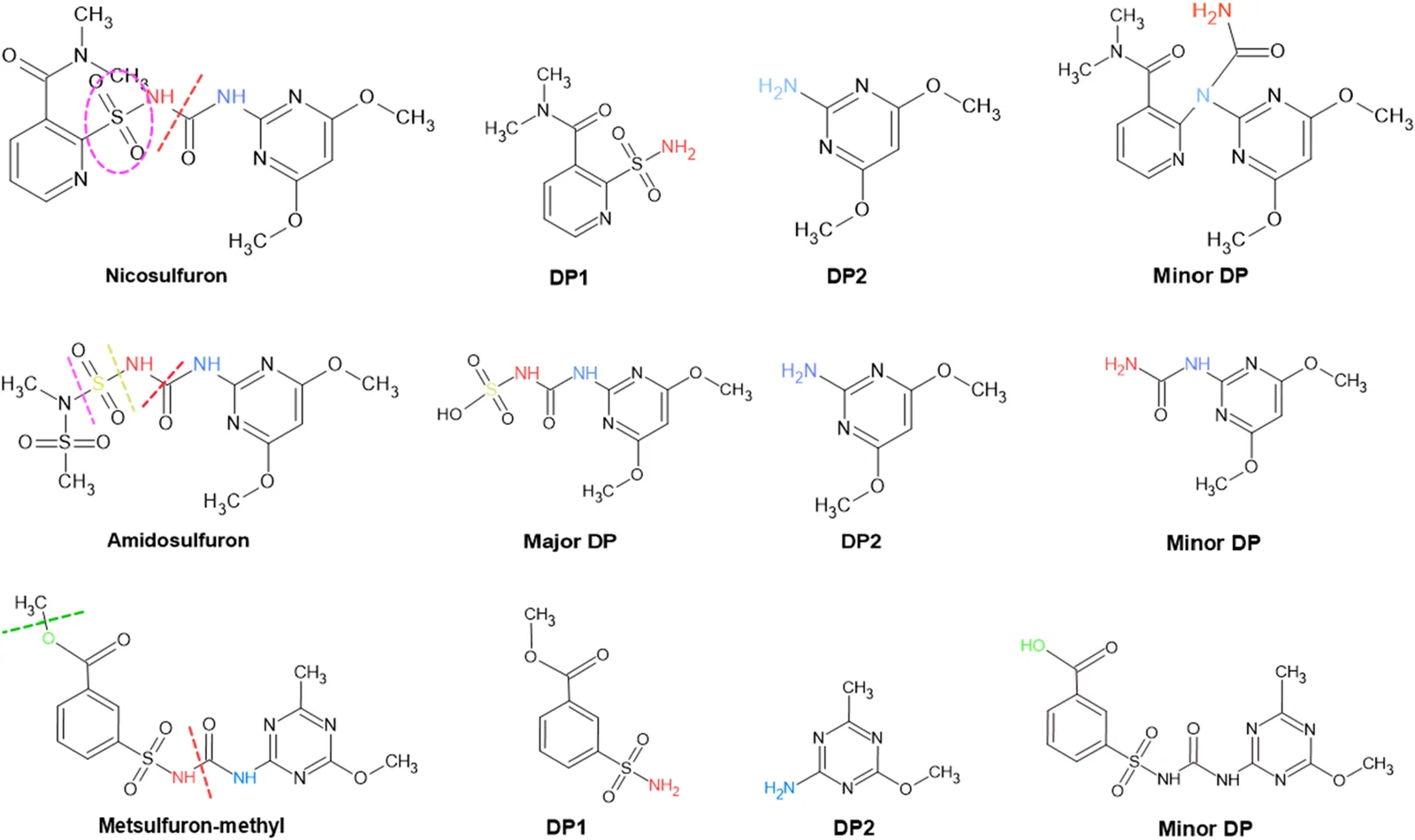
Structures of the investigated SUH herbicides and the main ceria-catalyzed hydrolysis degradation products identified by LC-MS.

### First-Principles Modeling of Adsorption and Hydrolysis of SUH on CeO_2_(111)

To complement the experimental findings and provide theoretical insights into the initial bond activation, first-principles DFT modeling was employed to evaluate the energetic profiles of the potential pathways. The complex and variegated nature of the differently prepared ceria NPs precludes the possibility of modeling them precisely. In this preliminary attempt, we use the minimum-energy (111) facet to represent ceria, which arguably reflects the state of the surface of a thermally annealed ceria particle rather than the highly defective and hydroxylated CePER nanomaterial. On the real, complex CePER surface, parallel and cooperative pathways—such as radical/redox actions or Brønsted acid–base mediation—likely operate in tandem with the proposed lattice-oxygen pathway below. While explicitly modeling these dynamic solid–liquid interfacial reactions remains a major computational challenge, the idealized model and current mechanism serve as a plausible fundamental baseline for understanding the coordination-driven activation of the sulfonylurea bridge. Without losing generality, the compound 1-(benzenesulfonyl)-3-phenylurea, referred to below as 1B3PhU, is chosen to represent SUHs. It has one unsubstituted aromatic group attached to the amine side and one to the S center. 1B3PhU has three ester bonds in the central urea group like all the herbicides tested above: an S–N bond, a N_*S*_–C bond on the sulfonyl side, and another C–N bond on the amine side.

The catalytic cycle is based on what has been proposed in the recent studies on the decomposition of generalized esters, including amides and sulfonamides.^38,39^ The resting state of the surface is CeO_2_(111) exposed to implicit water solvent but with two explicit adsorbed water molecules (state 1, Figure 9), one being a reactant in the reaction and the other approximately occupying the adsorption site of 1B3PhU. The adsorption of 1B3PhU replaces one of the water molecules (state 2, Figure 9), followed by the formation of a covalent bond with the surface via nucleophilic attack by O_latt_ on the central carbonyl C. This results in a tetrahedral (TI) intermediate state (state 4, Figure 9(2)), which activates the C–N bonds connected to the central C. The sulfonyl-side N–C bond is cleaved with an activation barrier (*E*_a_) of 0.21 eV adsorbed, producing a substituted amino group (C_6_H_5_–S­(=O)_2_–NH) coordinated to Ce and a phenylcarbamyl group (C_6_H_5_–NH–C=O) attached to O_latt_ (state 6, Figure 9(6)), which can also be viewed as a phenylcarbamate group occupying a lattice anion site (C_6_H_5_–NH–CO_2_/V_O_). Cleaving the S–N bond and the amine-side C–N bond (also in the presence of a coadsorbed water molecule) both has considerably higher *E*_a_ = 1.03 eV, suggesting that these are not the main reaction channels for 1B3PhU on this surface. Water then donates an H atom to the amino group forming benzenesulfonamide (C_6_H_5_–S­(=O)_2_–NH_2_) that is replaced by a water molecule and desorbs (states 8 → 9; Δ*G* = +0.25 eV). The remaining OH group displaces the carbamate group from the lattice site, forming a pair of adsorbed phenylcarbamate anion and proton (Figure 9(11)), which is replaced by water as phenylcarbamic acid (states 11 → 12; Δ*G* = +0.82 eV). The desorption of the oxy-anion is the most rate-controlling step in the catalytic cycle as is the case for other generalized esters,^38,39,88^ followed by the desorption of the sulfonamide with water. Once in the aqueous phase, a carbamic acid is expected to react rapidly with water to lose the remaining C–N bond to yield a primary amine (aniline in this case) and CO_2_. Depending on the pH, CO_2_ may be absorbed by the aqueous phase, adsorb onto ceria, or escape to the gas phase. The removal of CO_2_ drives the reaction in the forward direction. The overall hydrolysis reaction in the aqueous phase is slightly exergonic:

$$1B3{\mathrm{PhU}}_{\left(a\right)}+H_{2}O_{\left(a\right)}\to {\mathrm{benzenesulfonamide}}_{\left(a\right)}+{\mathrm{phenylcarbamic}}_{\left(a\right)}\mathrm{acid},,\,{\Delta G}_{\mathrm{rxn}}=-0.15\mathrm{eV}$$

$$\begin{array}{l} 1B3{\mathrm{PhU}}_{\left(a\right)}+H_{2}O_{\left(a\right)}\to {\mathrm{benzenesulfonamide}}_{\left(a\right)}+{\mathrm{aniline}}_{\left(a\right)}+{\mathrm{CO}}_{2}\left(g\right),,{\Delta G}_{\mathrm{rxn}}=-0.26\;eV\;\, \end{array}$$

Due to possible overbinding produced by optB86b-vdW,^89^ the reaction energy profile presented in Figure 9 is expected to be deeper on the energy scale than the actual reaction energy profile is. This energetic preference for sulfonyl-side N–C bond cleavage over amine-side C–N activation rationalizes the initial kinetic profile observed for NS. It explains why the sulfonamide derivative (DP1) form first, while the release of the amine residue (DP2) is delayed due to the subsequent hydrolysis of the remaining carbamyl intermediate.

**Figure 9:**
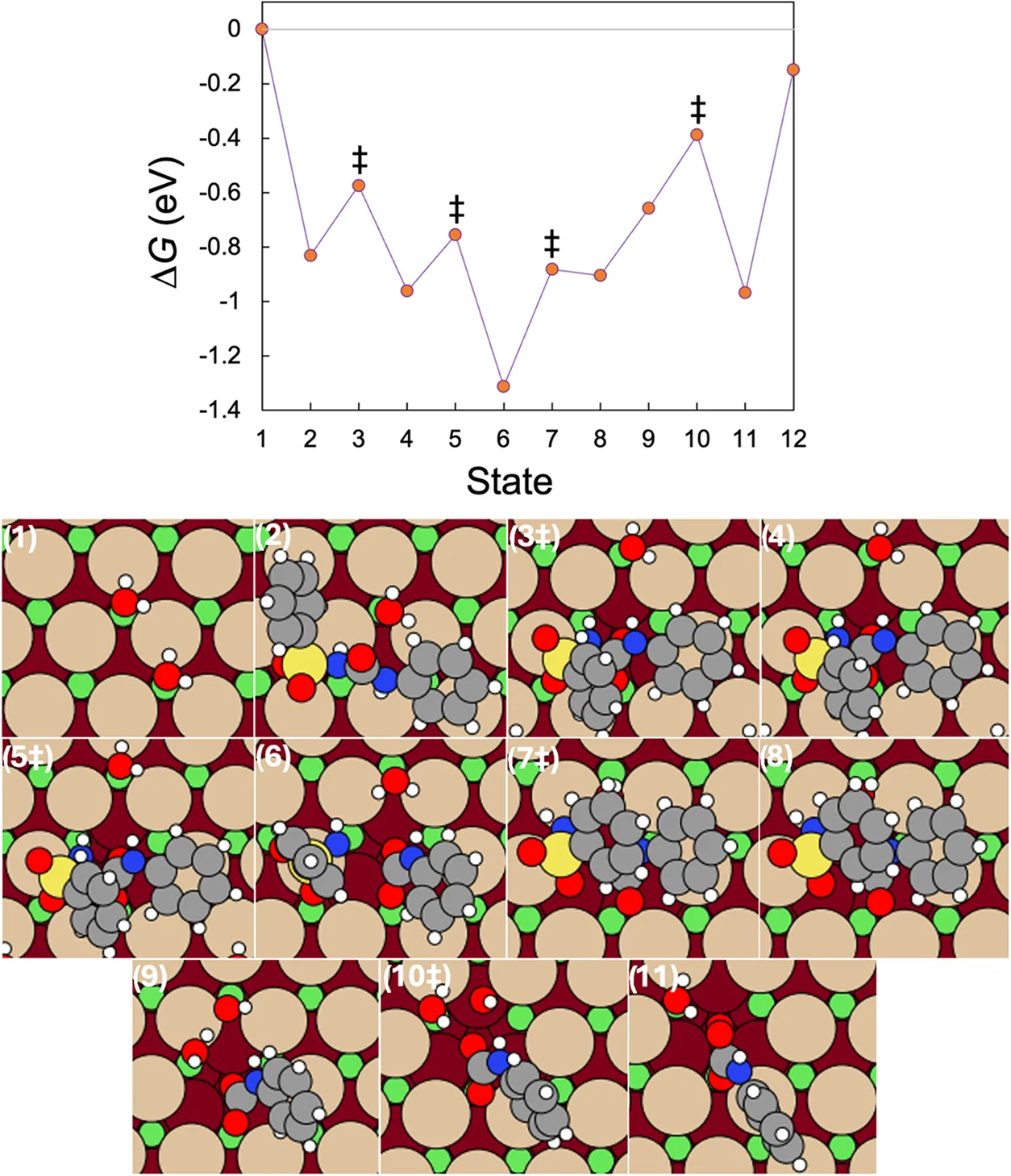
Top: DFT-calculated minimum-energy reaction energy profile for hydrolysis of 1B3PhU on CeO_2_(111). Bottom: Top view of the stable intermediate and transition (indicated by ‡) states of the elementary steps in the proposed reaction pathway (see Figure S33 for corresponding side views). Numbering of states corresponds to that in the top panel. The states shown are (1) water adsorption, (2) 1B3PhU adsorption, (3‡) TS of nucleophilic attack by O_latt_, (4) TI, (5‡) TS of N_*S*_–C bond scission, (6) amino + carbamyl, (7‡) TS of hydrogen transfer from H_2_O, (8) amine + OH + carbamyl, (9) OH + carbamyl, (10‡) TS of OH attack, and (11) carbamate + H. Color code: green = Ce; light and dark brown = top- and lower-layer O_latt_; red = O in molecules; black = C; blue = N; and white = H. O_latt_ bonded to C or H atoms are considered part of molecules.

The theoretical calculations thus help clarify the sequence of bond scissions in the decomposition of the SUHs. The desorption of the oxy-anion as acid (states 11 → 12) is the most kinetically limiting step on the reaction pathway as is the case for other generalized esters,^38,39,88^ with Δ*G* = +0.82 eV that can be estimated to be just higher than what is surmountable at ambient temperature. Crucially, this theoretical barrier (∼79.1 kJ/mol) is in line with the experimental apparent activation energy (*E*_a_ = 75.6 kJ/mol) derived from temperature-dependent kinetic experiments (Figure S13 and following *E*_a_ calculation in SI). This close correspondence provides strong support for the proposed lattice-oxygen-mediated mechanism and suggests that the overall reaction rate is governed by the energetics of surface-bound species and steps at ceria sites, rather than being diffusion-controlled. Clearly, the simple surface model and a single model compound used here cannot capture the range of reactivity of different SUHs on the different ceria nanoparticles. Differently substituted sulfonamide and carbamic acid species could alter the desorption energetics. The proposed mechanism predicts that more open crystalline facets of ceria would pose greater hindrance to the product desorption in this type of reaction,^39^ and that it would not be operative on a hydrogenated O_latt_ site since the key bond scission step involving the nucleophilic attack by O_latt_ would be blocked, implying that the higher the surface concentration of H, the less active a ceria surface should be for this reaction. This apparently contradicts the fact that CePER is the most active of the three types of samples toward SUH decomposition, which hints at rich catalytic chemistry occurring over CePER, including additional acid–base and/or redox actions based on the protons or their conjugate electrons. These possibilities will be the subject of future research.

## Conclusions

As shown here, nanoceria can initiate the spontaneous abiotic hydrolysis of sulfonylurea herbicides such as amidosulfuron, metsurfuron-methyl, and nicosulfuron, under mildly acidic conditions, without any additional reactants or illumination. Based on the identified major degradation products, experimental results demonstrate that the overall hydrolysis proceeds via urea N–C bond cleavage, although the experimental data alone cannot unambiguously differentiate which bond is cleaved initially. To further explore this, density functional theory (DFT) calculations were utilized to propose a specific mechanistic pathway, where the reaction is driven by a lattice-oxygen-mediated nucleophilic attack leading preferentially to sulfonyl-side N–C bond cleavage. The inherent acid–base (and associated superior redox) properties of nanoceria are responsible for this exceptional reactivity, which appears to be relatively unique among other transition metal oxide materials. NMR spectroscopy using probe molecules such as trimethylphosphine oxide has proven to be a sensitive method that can map the various acidic sites that play a significant role in the interfacial reactions studied here. However, as shown by the different reactivity among the prepared CeO_2_ samples, effective control over the synthesis conditions and the resulting surface and structural characteristics of the resulting catalyst is needed. Ultimately, our findings demonstrate that engineered and naturally occurring metal oxide interfaces can actively control the abiotic degradation kinetics and pathways of persistent agrochemicals. Furthermore, these insights suggest that nanoceria holds significant potential for a wide range of acid–base catalytic reactions relevant for environmental, industrial and biological processes.

## Supplementary Material



## Notes

Sample synthesis protocols; HPLC-DAD, LC-MS, and NMR measurement details; additional adsorption, kinetic and blank tests; time-resolved chromatograms; MS spectra of standards, reaction mixtures, and identified DPs; additional SEM and TEM figures; nitrogen physisorption measurements data (PDF)
