# Supplementary material for: Nanoceria as an Acid–Base Catalyst for Interfacial Hydrolysis of Sulfonylureas: A Combined 31P MAS NMR and DFT Study
Source: Inorg Chem. 2026 Oct 23;65(44):26168–83. doi: 10.1021/acs.inorgchem.6c04712 (PMC13753729; doi:10.1021/acs.inorgchem.6c04712)
Supplement: Supplementary file 1 [file ic6c04712_si_001.pdf]

SUPPLEMENTARY INFORMATION

Nanoceria as an Acid-base Catalyst for Interfacial Hydrolysis of Sulfonylureas: A Combined ^{31}P MAS NMR and DFT Study

Jan Čundrle,[†,‡] *Martin Šťastný,*[†] *Tereza Dušková,*[§] *Dmytro Bavol,*[†] *Daniela Popelková,*[†,¶] *Jakub Ederer,*[‡] *Nusrat Jahan Rifat,*[∥] *Ye Xu,*[∥] *Jiří Henych,*[*†,‡]

† Institute of Inorganic Chemistry of the Czech Academy of Sciences, 250 68 Husinec-Řež, Czechia

‡ Faculty of Environment, Jan Evangelista Purkyně University in Ústí nad Labem, Pasteurova 3632/15, 400 96 Ústí nad Labem, Czechia

§ Faculty of Science, Jan Evangelista Purkyně University in Ústí nad Labem, Pasteurova 3632/15, 400 96 Ústí nad Labem, Czechia

∥ Cain Department of Chemical Engineering, Louisiana State University, Baton Rouge, Louisiana 70803, United States

¶ Charles University Prague, Faculty of Science, Imaging Methods Core Facility BIOCEV, Vestec 252 50, Czech Republic

*Corresponding author e-mail address: henych@iic.cas.cz

Sample synthesis details. CeAMM: 1 L of an aqueous solution of $Ce(NO_3)_3.6H_2O$ was heated at 60 ˚C in a double-walled glass reaction apparatus (1 L, Atlas HD, Syrris, UK) with constant stirring and a continuous supply of air, which was previously purged of CO_2 by passing it through a 1 M KOH solution. After reaching the target temperature, the mixture was precipitated with 20 mL of 30% aqueous ammonia solution. The mixture was further stirred for 3.5 h, during which time the characteristic yellow precipitate of CeO_2 was formed. After cooling, the suspension was washed with distilled water using a decantation and dialysis membrane (Membra-Cel™ cellulose tubing, 44 mm, MWCO 14,000 Da) and then filtered, air-dried and finely ground using a mortar and pestle.

CePER: 0.1 L of an aqueous solution of $Ce(NO_3)_3.6H_2O$ (0.1 M) was precipitated by the addition of 20-25 mL of 1 M aqueous NaOH solution to form a gelatinous precipitate, achieving a pH ≈ 11. The precipitate was washed with distilled water by decantation and 50 mL of 30% H_2O_2 solution was carefully added while cooling in an ice bath. The resulting red-brown precipitate was left aging for 30 min, then transferred to a reflux apparatus and heated under

reflux at 100 ˚C. The resulting yellowish precipitate was further washed by decantation and dialysis membrane, then filtered, air-dried and finely ground using a mortar and pestle.

CeUREA: 1 L of an aqueous solution of $Ce(NO_3)_3.6H_2O$ (0.02 M) acidified with 3 mL of HNO3 (65 %) was mixed with 200 g of urea. The mixture was heated to 92 ˚C for 5 h with constant stirring, and continuously the mixture was replenished with warm dH_2O, which was gradually evaporated. After cooling, the white precipitate was washed several times with water by decantation, air-dried and finely ground using a mortar and pestle. This was followed by calcination for 2 hours at 500 ˚C with a temperature rise of 5 ˚C/min.

TiO_2: 1 L of an aqueous solution of $TiOSO_4$ (0.02 M) acidified with 5 mL of H_2SO_4 (98 %) was mixed with 100 g of urea. The mixture was heated to 92 ˚C for 4 h with constant stirring, and continuously the mixture was replenished with warm dH_2O, which was gradually evaporated. After cooling, the white precipitate was washed several times with water by decantation, air-dried and finely ground using a mortar and pestle.

HPLC/DAD measurement details. HPLC system was equipped with a high-pressure pump, column thermostat, autosampler and DAD detector. An Accucore™ PFP column, grain size 2.6 μm, pentafluorophenyl phase with alternative selectivity to C18 (manufactured by Thermo Fisher Scientific, USA) with dimensions of 150 x 4.6 mm was used for the separation of SUHs herbicides and their hydrolytic products. The gradient elution with the following composition of the mobile phase was used: acetonitrile with 0.1 % HCOOH (compartment C) and water with 0.1 % HCOOH (compartment A). The flow rate of the mobile phase was 1 mL/min. The separation

was realized at a constant temperature of 30 ˚C and the injection volume was 40 µl. The gradient elution settings for individual herbicides and their hydrolytic products are presented below (Table S1) together with the specific optimized conditions for the detection of herbicides and their hydrolytic products (Table S2).

LC-MS measurement details. A Vanquish Core HPLC system equipped with a Diode Array detector (DAD) and further connected to an Orbitrap Exploris™ 120 spectrometer detector were used to separate and identify the newly formed degradation products. Chromatographic conditions were as follows: Accucore PFP column (2.6 µm, 150 x 4.60 mm *I.D.*) at 30 ˚C; gradient elution with ACN/H_2O (0.1% FA, HPLC gradient grade, Aldrich) from 15/85 (0 min) linearly to 95/5 (6 min), subsequently off the record linearly back to 15/85 (in 30 sec) for the equilibration (2.5 min); flow rate, 0.7 mL/min; detection, DAD (190–800 nm). Samples of the standard were dissolved in H_2O to a final concentration of 2 mg/L. The injection volume of all samples was 40 µL. High-Resolution Mass Spectrometry (HRMS) measurements were performed using a HESI probe (Heated Electrospray Ionisation) in both negative and positive modes, with nitrogen (4.8 Air Products) as the collision gas. By comparison, the experimental isotopic distribution in the plot of the peaks in the measured spectra corresponded fully to the calculated spectral pattern. Conditions used for the ESI interface: vaporizer temperature 60 ˚C; N_2 (isolated from air in Genius XE35, Peak Scientific) as a nebulizing sheath gas and auxiliary gas, flow 45 arb. and 15 arb., respectively; spray voltage 3.5 kV; ion transfer tube temperature 320 ˚C; RF lens 10% and mass range from 50 to 600.

Table S1. Gradient elution setup for HPLC/DAD system.

Time (min)	%A	%C	Flow rate (mL/min)
-2 (equilibration)	85	15	1
0	85	15	1
6	5	95	1

Table S2. Optimized conditions for the analysis of individual drugs and their degradation products on both analytical systems.

Analyte	Wavelength (nm)	Retention time/HPLC-DAD (min)*	Retention time/LC-MS (min)**
Amidosulfuron	235	5.46	5.53
Metsulfuron-methyl	228	5.24	5.33
Nicosulfuron	235	4.63	4.70
2-amino-4,6-dimethoxypyrimidine	260	4.11	3.23
N,N-Dimethyl-2-sulfamoylnicotinamide	260	2.09	2.17
2-amino-4-methoxy-6-methyl-1,3,5-triazine	260	2.29	2.11
Methyl 2-(aminosulfonyl)benzoate	260	4.17	4.33

**Retention times corresponding to kinetic profiling and HPLC-DAD chromatograms in the main text (Figure 6). | **Retention times corresponding to high-resolution mass spectrometry product identification and HRMS spectra in the Supporting Information (Figures S20–S32).*

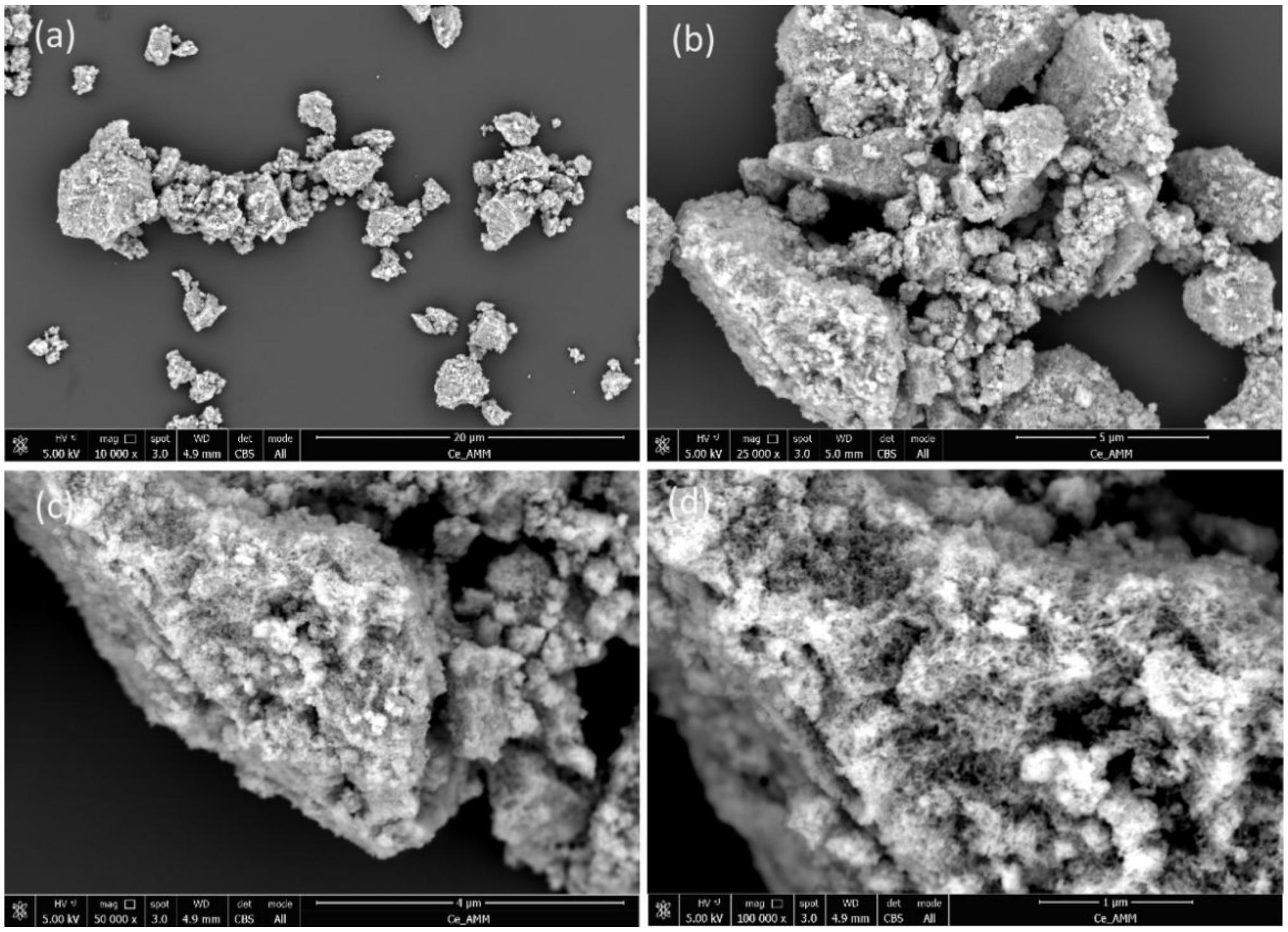

Figure S1. SEM images of CeAMM, magnification (a) 5kx, (b) 25kx, (c) 50kx, and (d) 100kx.

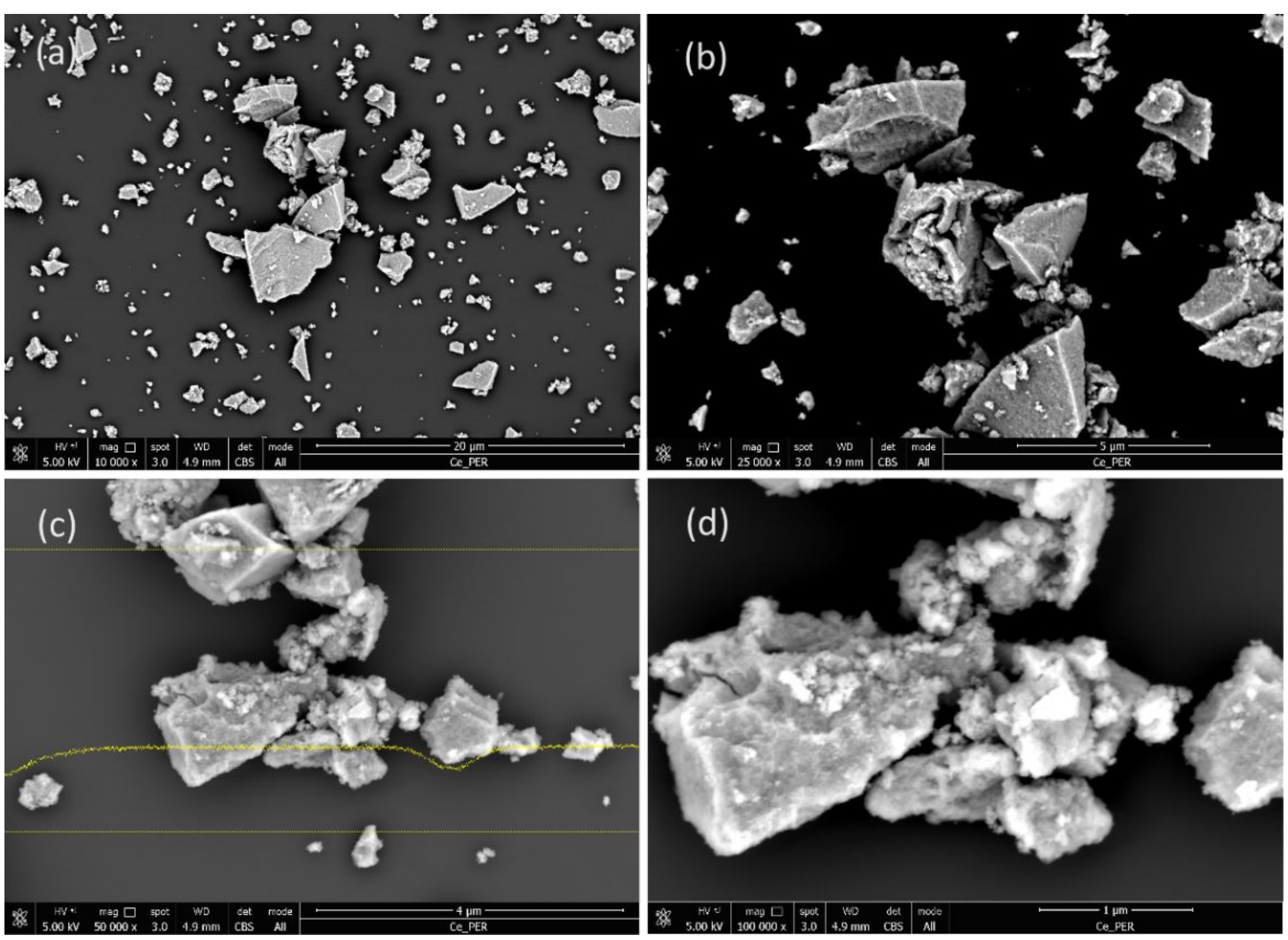

Figure S2. SEM images of CePER magnification (a) 5kx, (b) 25kx, (c) 50kx, and (d) 100kx.

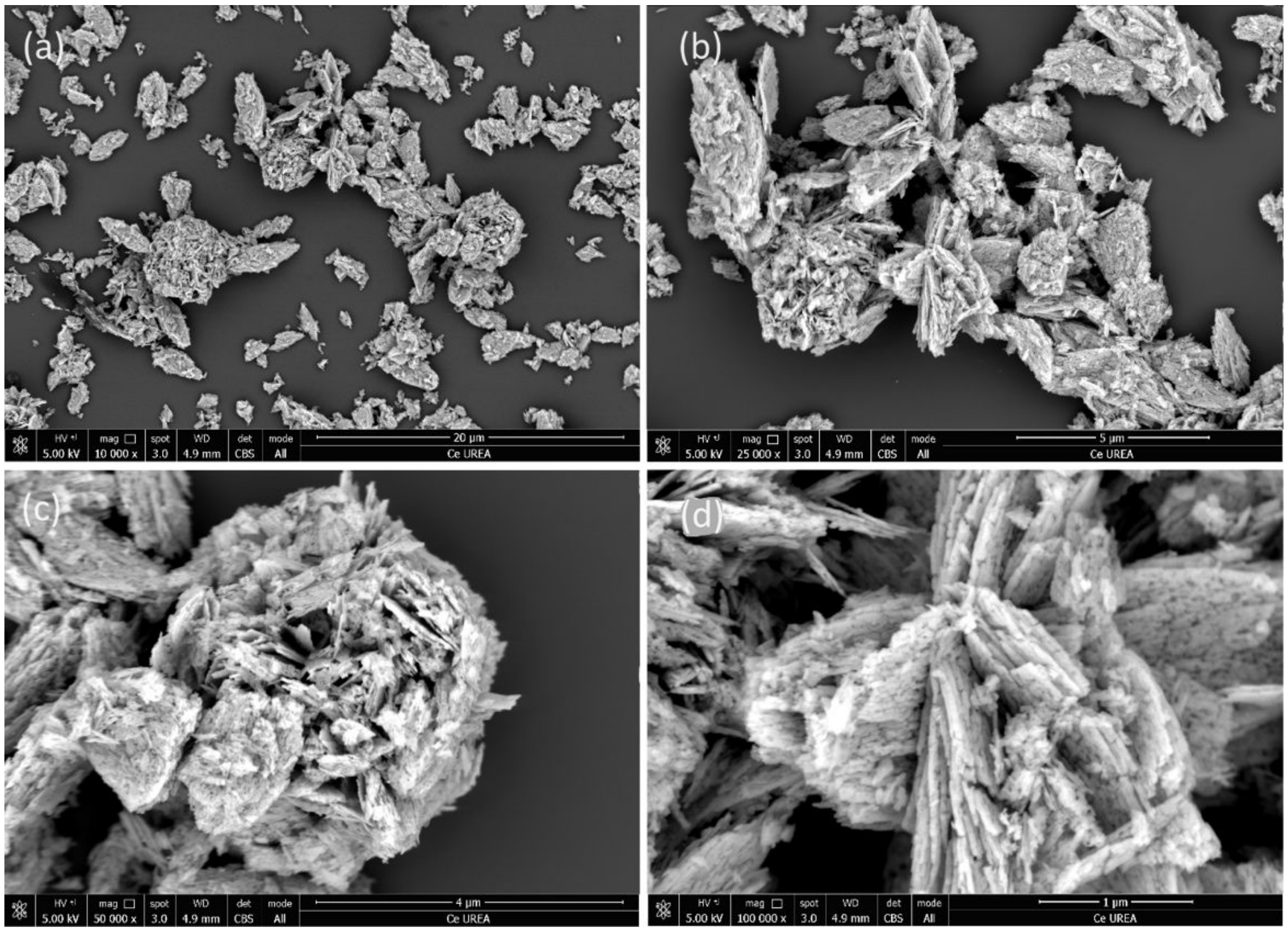

Figure S3. SEM images of CeUREA magnification (a) 5kx, (b) 25kx, (c) 50kx, and (d) 100kx.

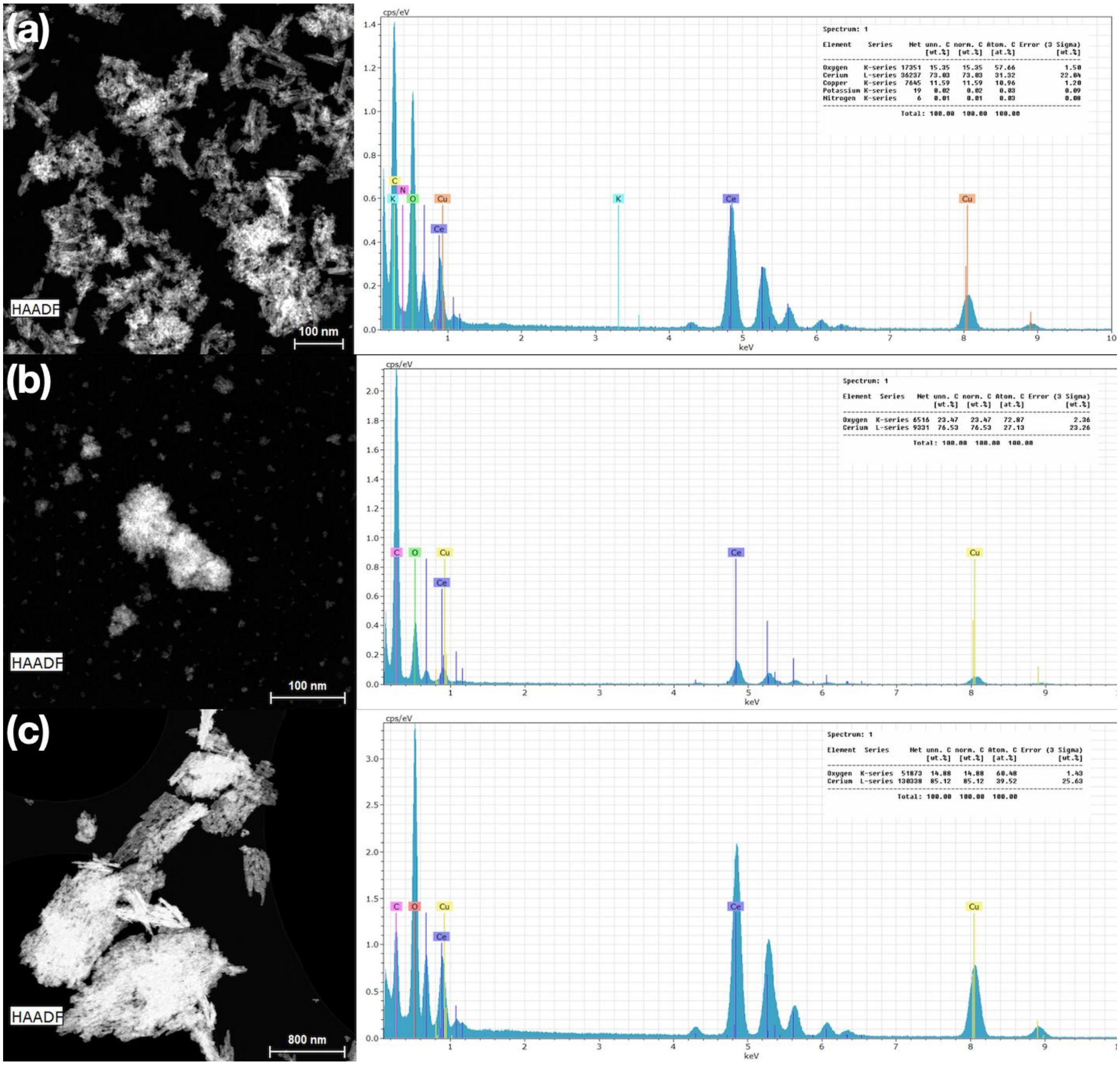

Figure S4. TEM/EDS of (a) CeAMM, (b) CePER and (c) CeUREA.

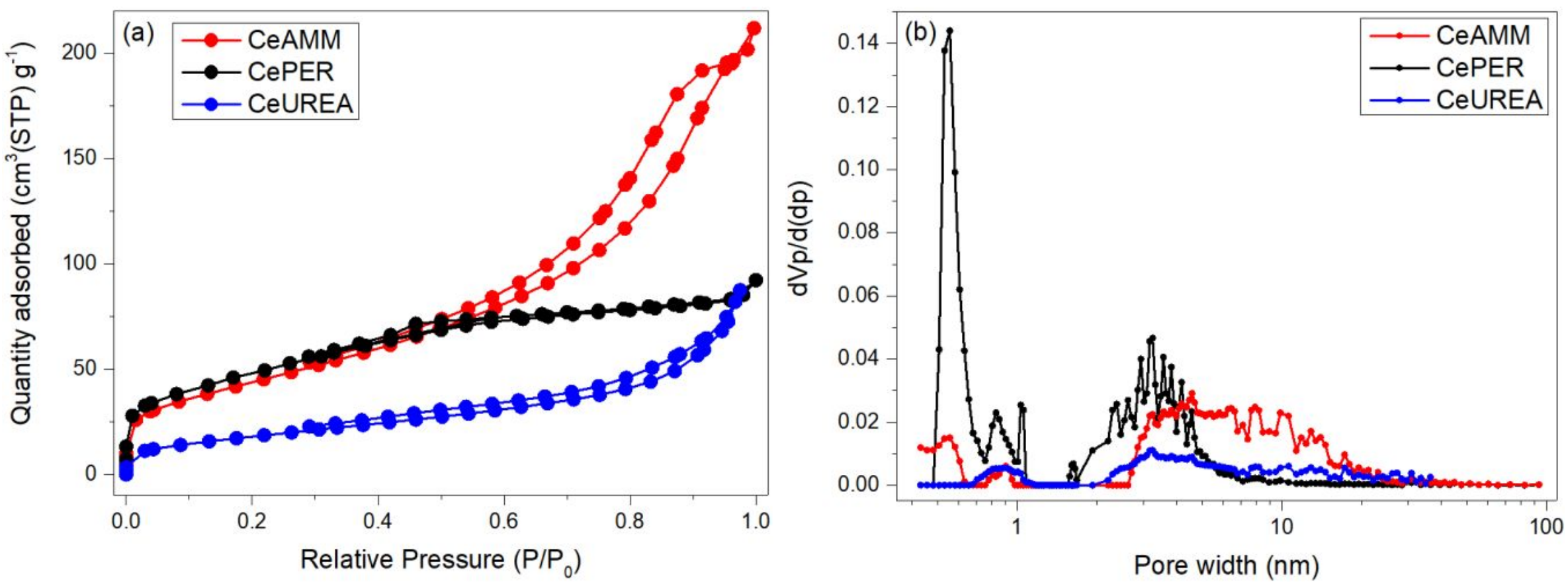

Figure S5. (a) Adsorption-desorption isotherms and (b) pore size distribution obtained from NLDFT analysis of prepared ceria samples.

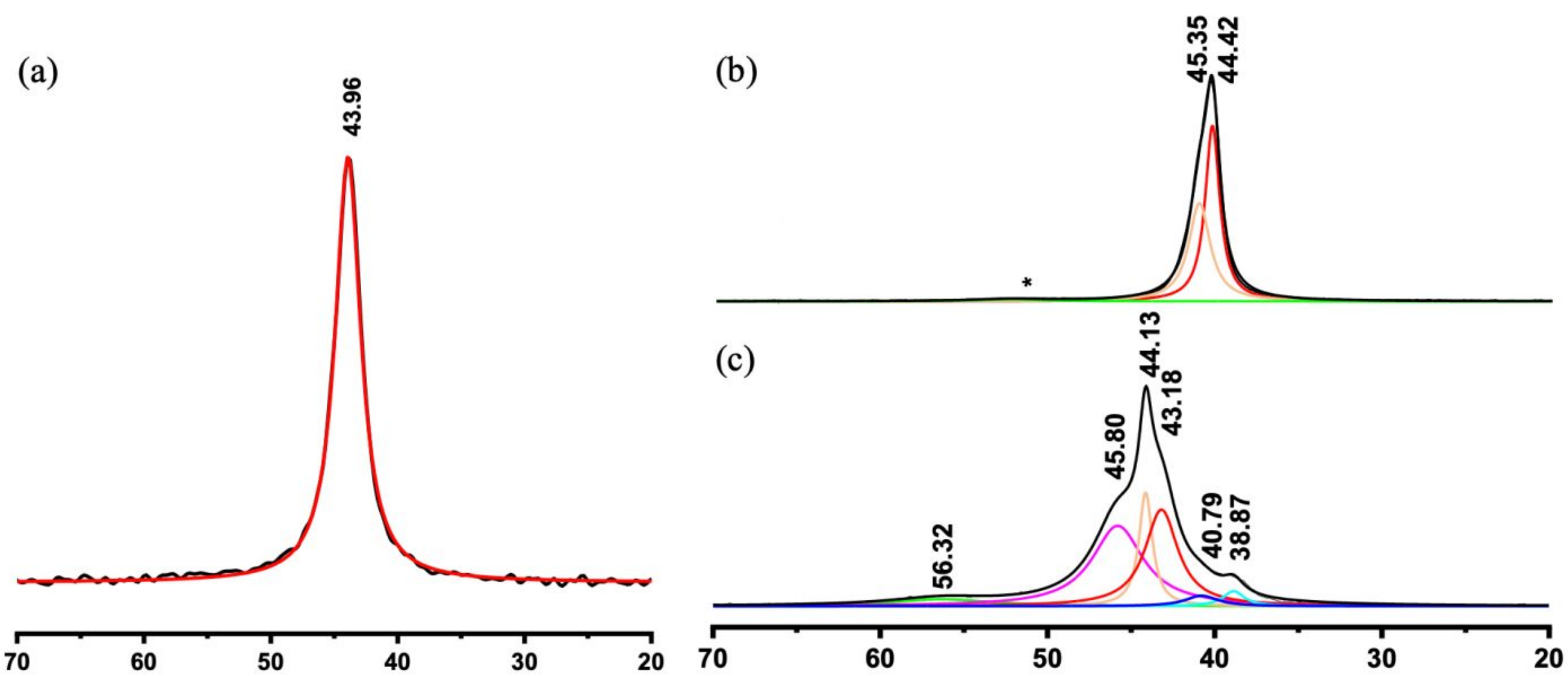

Figure S6. The single-pulse ^{31}P MAS NMR deconvoluted spectra of TMPO adsorbed on (a) SiO_2, (b) CeAMM500, and (c) CePER500. The asterisk (*) indicates a low–intensity signal (~56 ppm) near detection limit (S/N ≈ 7:1)

Recycling experiments. Recycling experiments were performed analogously to the standard hydrolysis experiments, but on an increased scale, using 250 mg of CePER in 30 mL of aqueous

solution containing NS at the same concentration (10 mg/L). The experiments were conducted in 50 mL polypropylene tubes to minimize material losses associated with the larger-scale setup. The CePER suspension was dispersed in an ultrasonic bath, after which NS was added from a stock solution. At predetermined time intervals, 0.2 mL aliquots of the suspension were withdrawn, diluted with 0.8 mL of deionized water in 2 mL Eppendorf tubes, vortexed for 3 s, and centrifuged at 18,000 rpm for 2 min. The supernatants were analyzed immediately by HPLC-DAD. After completion of the reaction, the solid material was recovered by centrifugation, washed three times with deionized water, and dried overnight at 60 ˚C. A total of three cycles was performed.

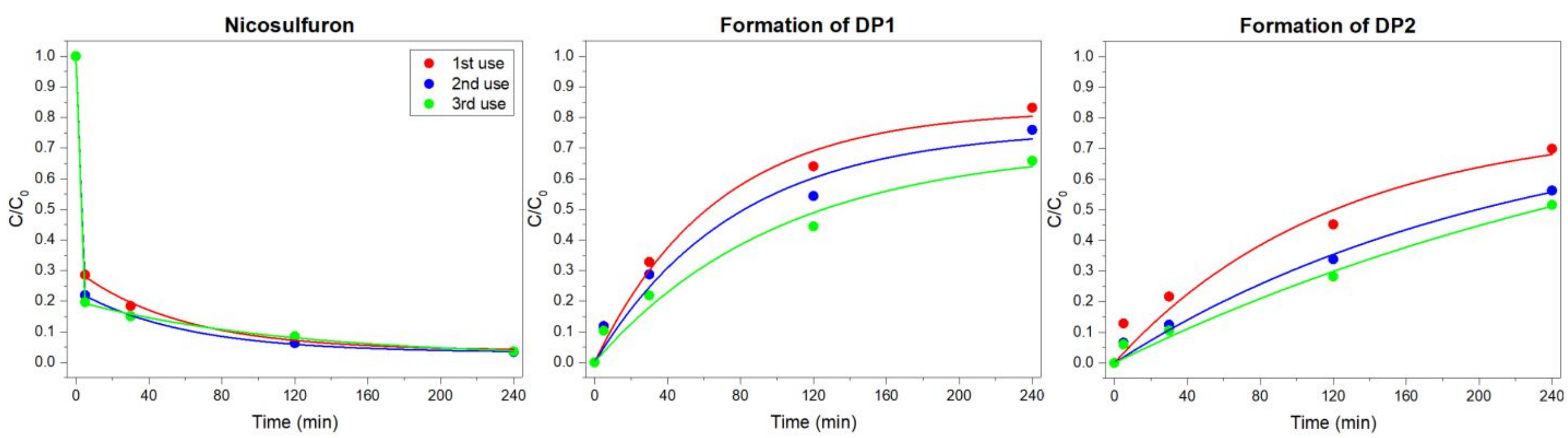

Figure S7. Recycling experiment of the CePER-catalyzed hydrolysis of NS.

Simultaneous degradation. The simultaneous degradation of all three sulfonylurea herbicides on CePER was tested in a single reaction mixture. The kinetics observed for each compound closely resemble those obtained in individual experiments, with nicosulfuron showing the fastest degradation, followed by metsulfuron-methyl and amidosulfuron. Nicosulfuron was degraded most efficiently, with rapid loss of the parent compound and formation of DP-NS1 and DP-NS2=DP-AS2. Metsulfuron-methyl showed slower conversion, with only DP-MSM1 being

formed; DP-MSM2 was not detected under these conditions. Amidosulfuron showed the slowest degradation among the three compounds, forming DP-AS2. These results indicate that while CePER retains high activity for nicosulfuron, the presence of multiple substrates may partially suppress the degradation of less reactive compounds.

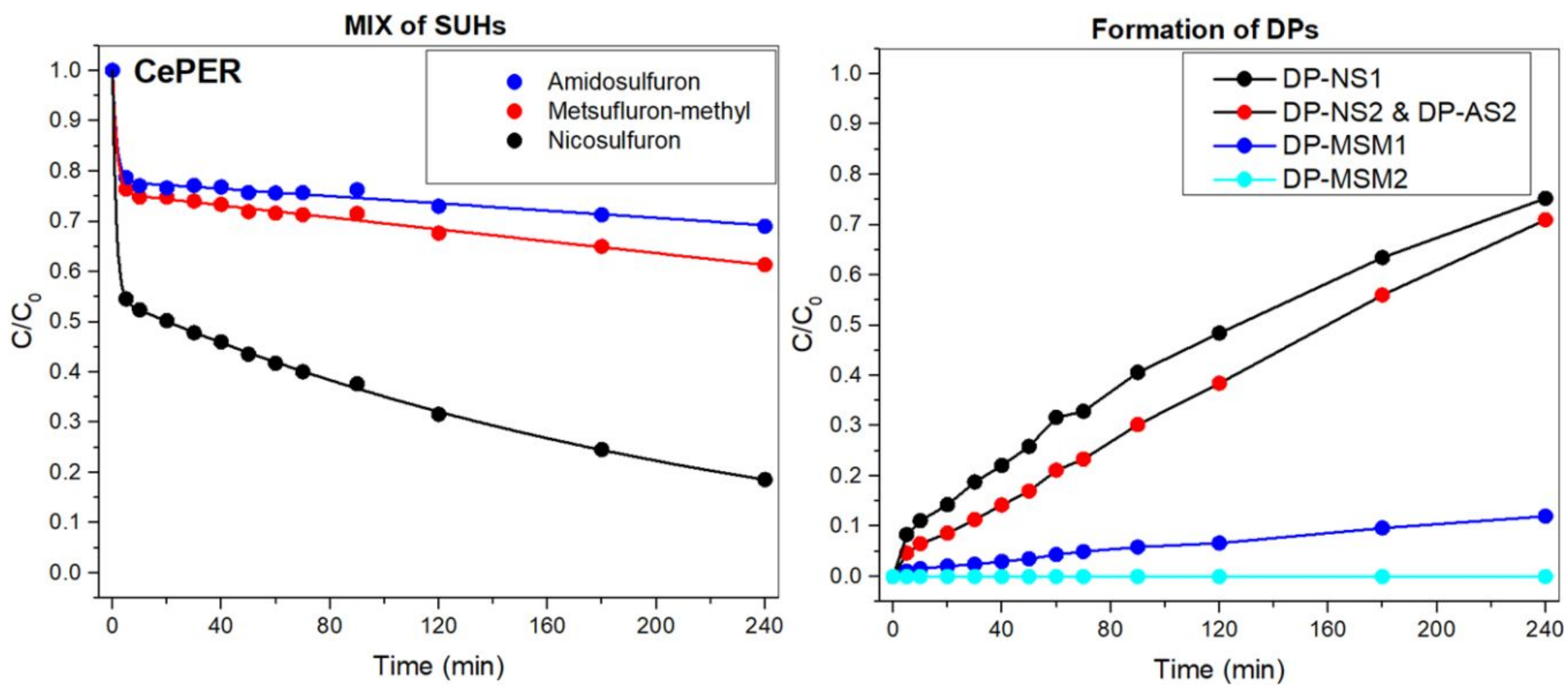

Figure S8. Kinetics of (a) simultaneous degradation of a ternary mixture of all three sulfonylurea herbicides catalyzed by CePER and (b) formation of the corresponding degradation products (DPs), where NS stands for nicosulfuron, AS for amidosulfuron and MSM for metsulfuron-methyl. Note that DP2 is the same for NS and AS.

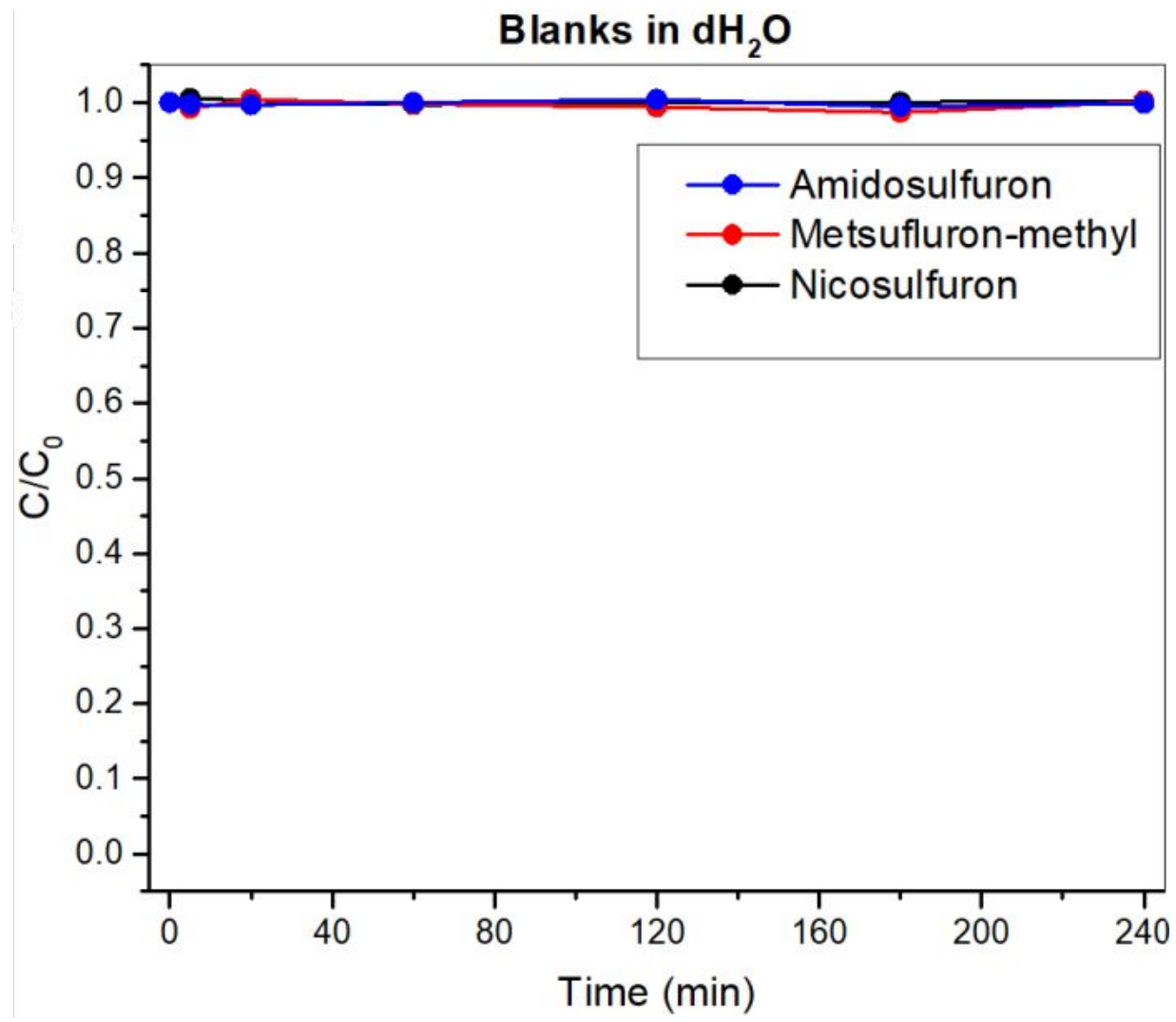

Figure S9 Stability of all three sulfonylurea herbicides in deionized water (dH_2O) without the catalyst.

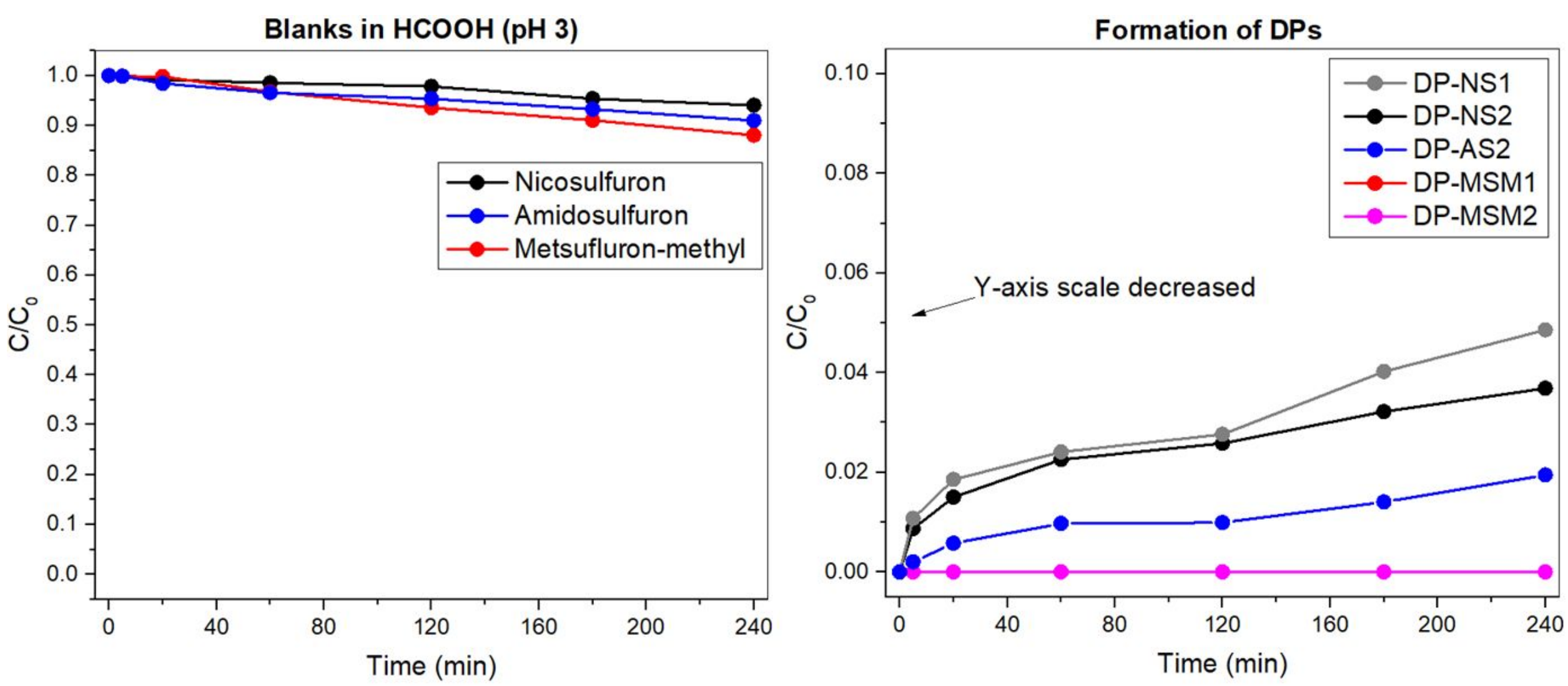

Figure S10. Stability of all three sulfonylurea herbicides in acidic aqueous solution (pH ~3) without the catalyst (left) and formation of degradation products (DPs), shown with expanded y-axis scale (right).

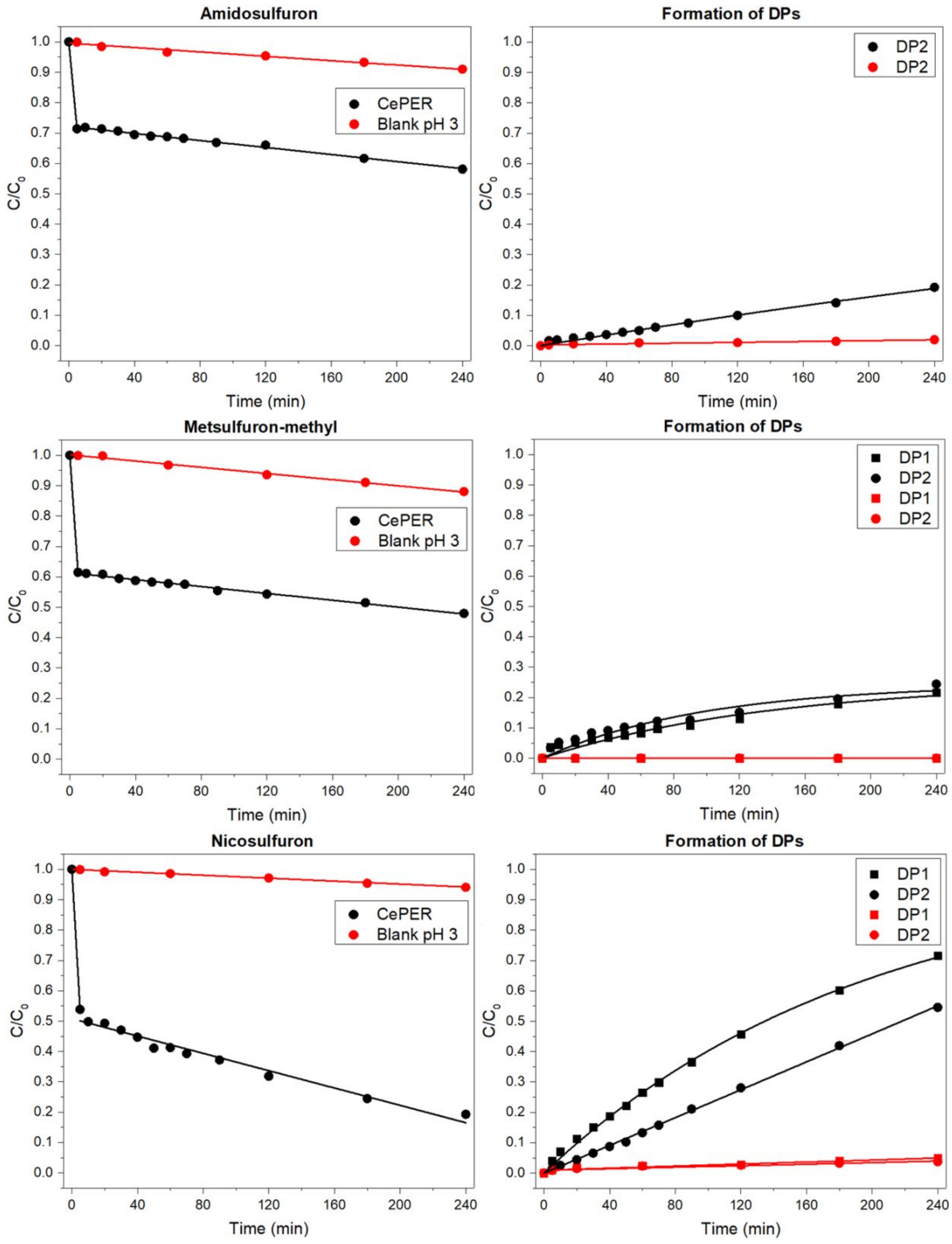

Figure S11. Comparison of the degradation kinetics of three sulfonylurea herbicides catalyzed by CePER versus uncatalyzed acidic hydrolysis at pH ~3 (left) and the kinetics of formation of the corresponding degradation products (right).

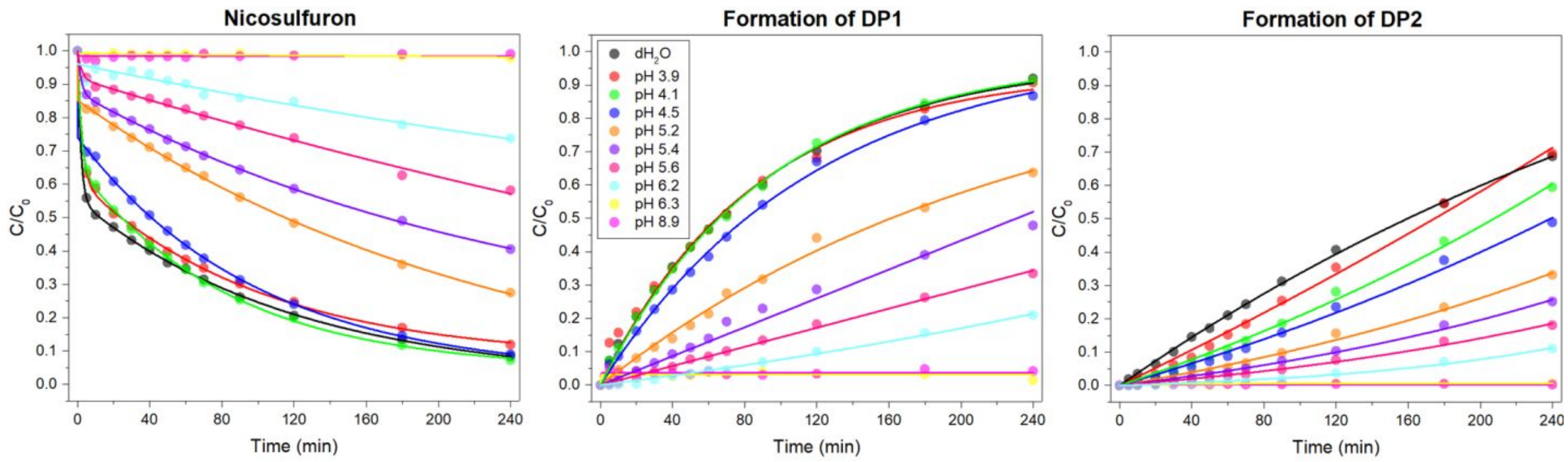

Figure S12. Kinetics of NS degradation on CePER under various pH (left) and the formation of degradation products DP1 (middle) and DP2 (right).

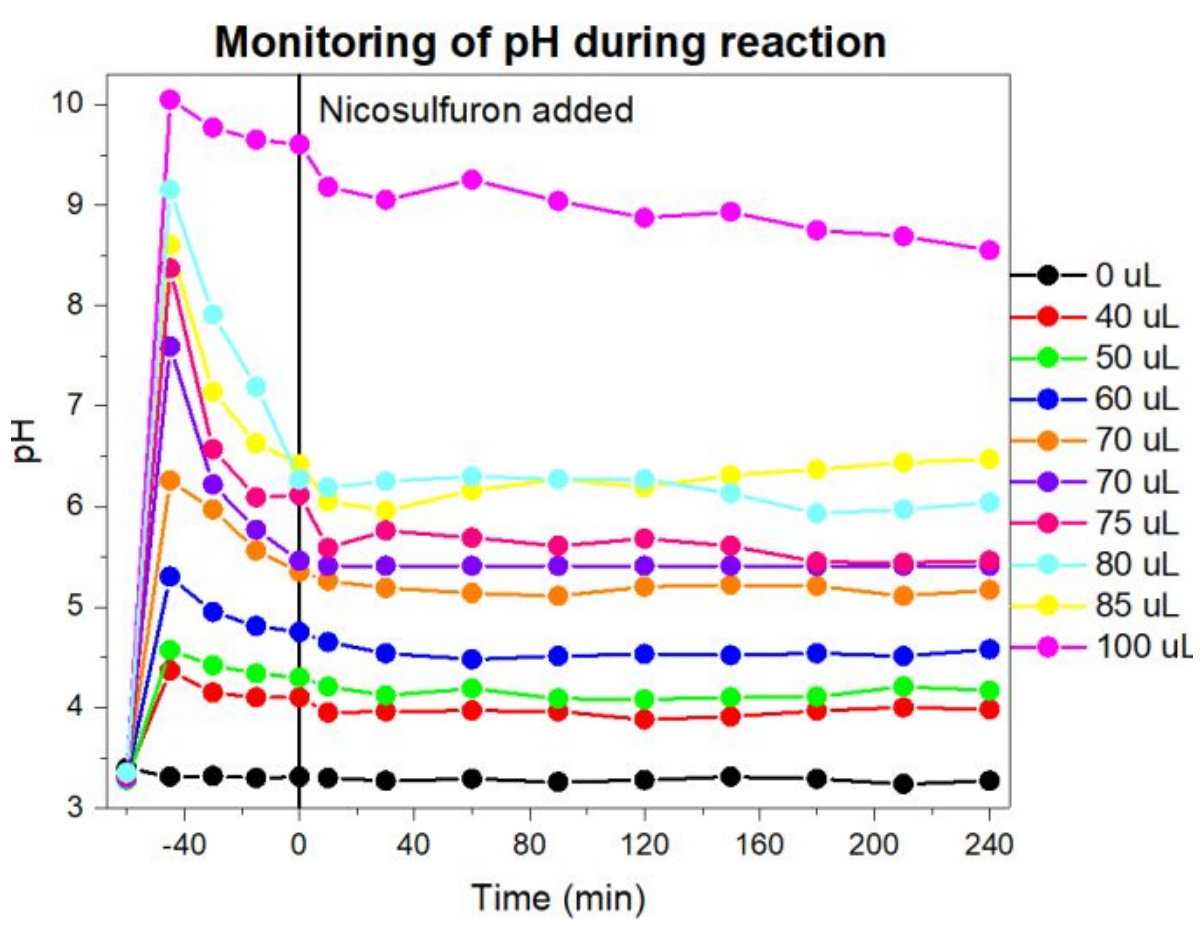

Figure S13. In situ monitoring of pH during the catalytic degradation of NS on CePER under various initial pH conditions. The vertical line at time zero marks the addition of NS to the reaction mixture.

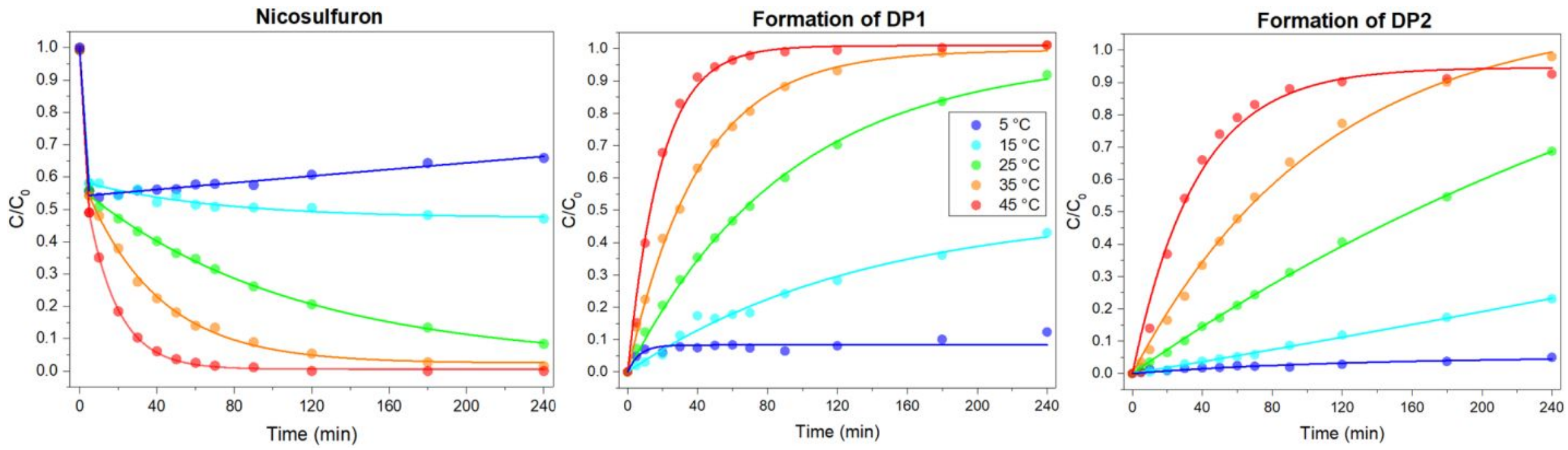

Figure S14. Kinetics of CePER-catalyzed NS degradation at different temperatures (left), and the formation of the corresponding DP1 (middle) and DP2 (right).

Activation energy calculation. The activation energy (*Ea*) of the CeO_2-catalyzed degradation of NS was determined from temperature-dependent kinetic data (Figure S13). Apparent rate constants (k) were obtained by fitting the concentration profiles (C/C_0) to a pseudo-first-order kinetic model using the equation: $\ln(C/C_0) = -kt$

Fitting was performed over the time interval 5–240 minutes; the initial point at 0 minutes ($C/C_0 = 1$) was excluded to avoid bias from early adsorption effects. The dataset obtained at 5 and 15 ˚C were excluded from the Arrhenius analysis due to anomalous behavior—likely resulting from desorption of NS—which deviated from the expected kinetic trend and distorted the linear correlation. A plot of ln(k) versus ($1/T$) (Arrhenius plot) was constructed, and *Ea* was calculated from the slope of the linear regression line according to the Arrhenius relation: slope = $-E_a/R$. The resulting ***Ea* is 75.6 kJ/mol** and provides insight into the temperature dependence and mechanistic nature of the catalytic degradation process.

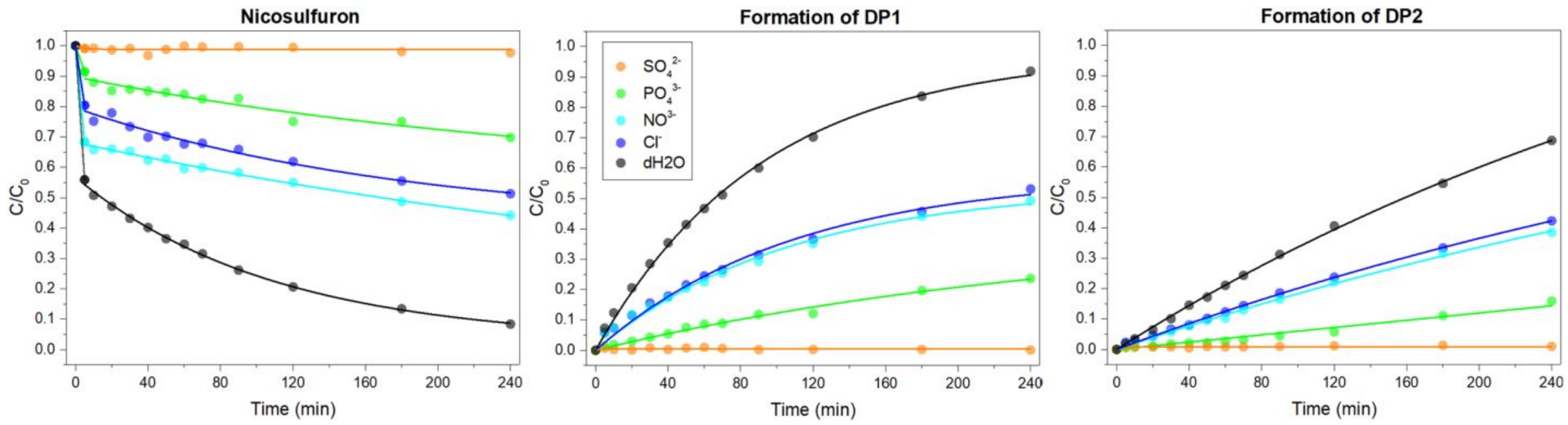

Figure S15. Kinetics of CePER-catalyzed NS degradation in the presence of various anions (SO_4^{2-}, PO_4^{3-}, NO_3^-, Cl^-) and in deionized water (dH_2O) (left) and kinetics of DP1 (middle) and DP2 formation (right).

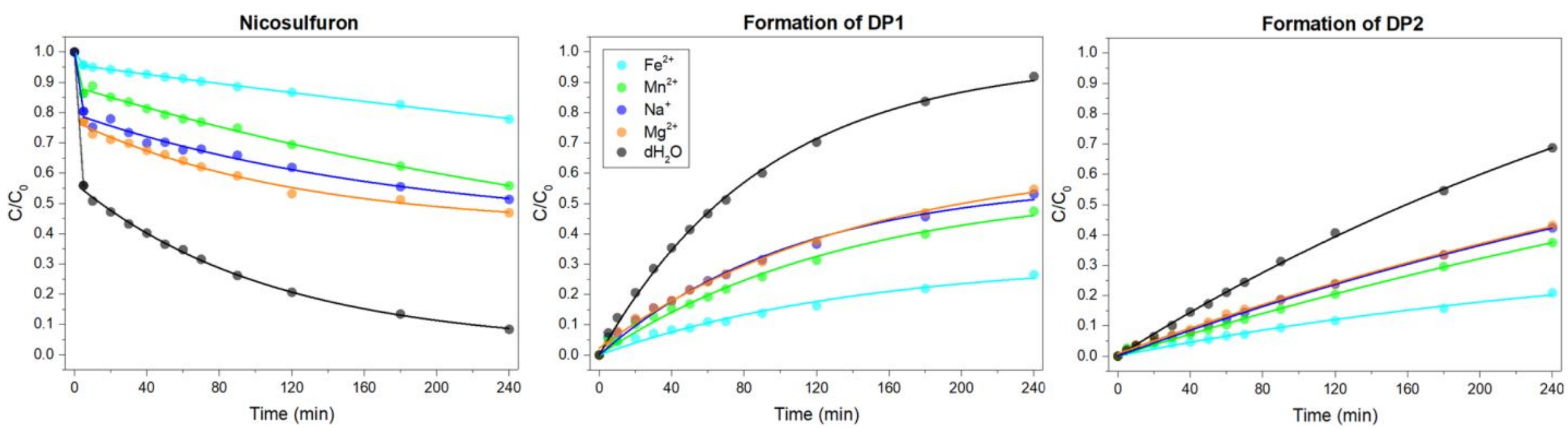

Figure S16. Kinetics of CePER-catalyzed NS degradation in the presence of various cations (Mg^{2+}, Mn^{2+}, Fe^{2+}, Na^+) and in deionized water (dH_2O) (left) and kinetics of DP1 (middle) and DP2 formation (right).

Table S3. Summary of initial pH values of aqueous solutions of selected salts and the corresponding pH of CePER suspensions before and after the catalytic reaction

Ions	pH of 1 mM water solution	*pH with CePER dispersion	pH after reaction
dH_2O	6,25	3,37	3,26
Na^+ / Cl^-	6,07	3,54	3,44
Fe^{2+}	5,59	3,62	3,61
Mn^{2+}	5,99	3,58	3,54
Mg^{2+}	5,92	3,62	3,56
NO_3^-	6,16	3,59	3,49
PO_4^{3-}	5,39	3,67	3,59
SO_4^{2-}	6,25	5,20	5,11

* 10 mg CePER in 4 mL 1 mM ions solution

Table S4. Tentative quantification of the contribution of adsorption, acid-induced catalysis, and ceria-catalyzed reaction based on C/C_0 kinetics and product mass balance.

Herbicide	Time point	Initial adsorption (5 min)	Homogeneous Acid Hydrolysis	Heterogeneous Surface Catalysis	Persistent Surface Adsorbed / Alternative Products
Nicosulfuron (NS)	240 min	~46 %	< 3 %	> 80 %	< 5 %
	1440 min	—	< 5 %	~100 %	~0 %
Amidosulfuron (AS)	240 min	~28 %	< 2 %	~20–25 %	~15–20 % (Slow surface kinetics)
	1440 min	—	< 5 %	~50–60 %	~35–45 % (Continuous slow release)
Metsulfuron-methyl (MSM)	240 min	~39 %	< 2 %	~20–25 %	~15–20 % (Locked on surface)
	1440 min	—	< 5 %	~50–60 %	~35–45 % (Plateau / Side-products)

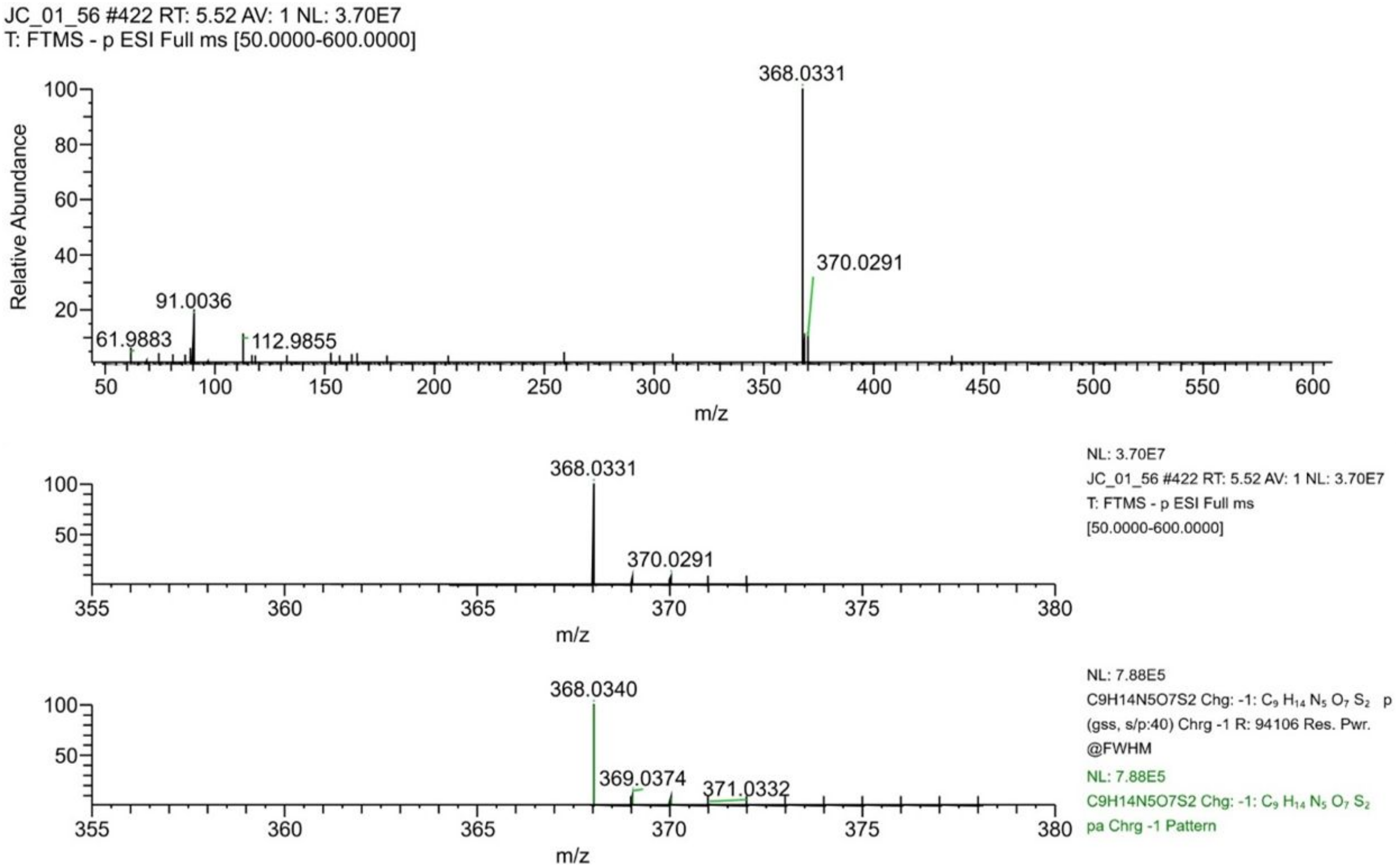
JC_01_56 #422 RT: 5.52 AV: 1 NL: 3.70E7
T: FTMS - p ESI Full ms [50.0000-600.0000]
368.0331
370.0291
91.0036
61.9883
112.9855
Relative Abundance
m/z
NL: 3.70E7
JC_01_56 #422 RT: 5.52 AV: 1 NL: 3.70E7
T: FTMS - p ESI Full ms
[50.0000-600.0000]
368.0340
369.0374
371.0332
NL: 7.88E5
C9H14N5O7S2 Chg: -1: C9 H14 N5 O7 S2 p
(gss, s/p:40) Chrg -1 R: 94106 Res. Pwr.
@FWHM
NL: 7.88E5
C9H14N5O7S2 Chg: -1: C9 H14 N5 O7 S2
pa Chrg -1 Pattern

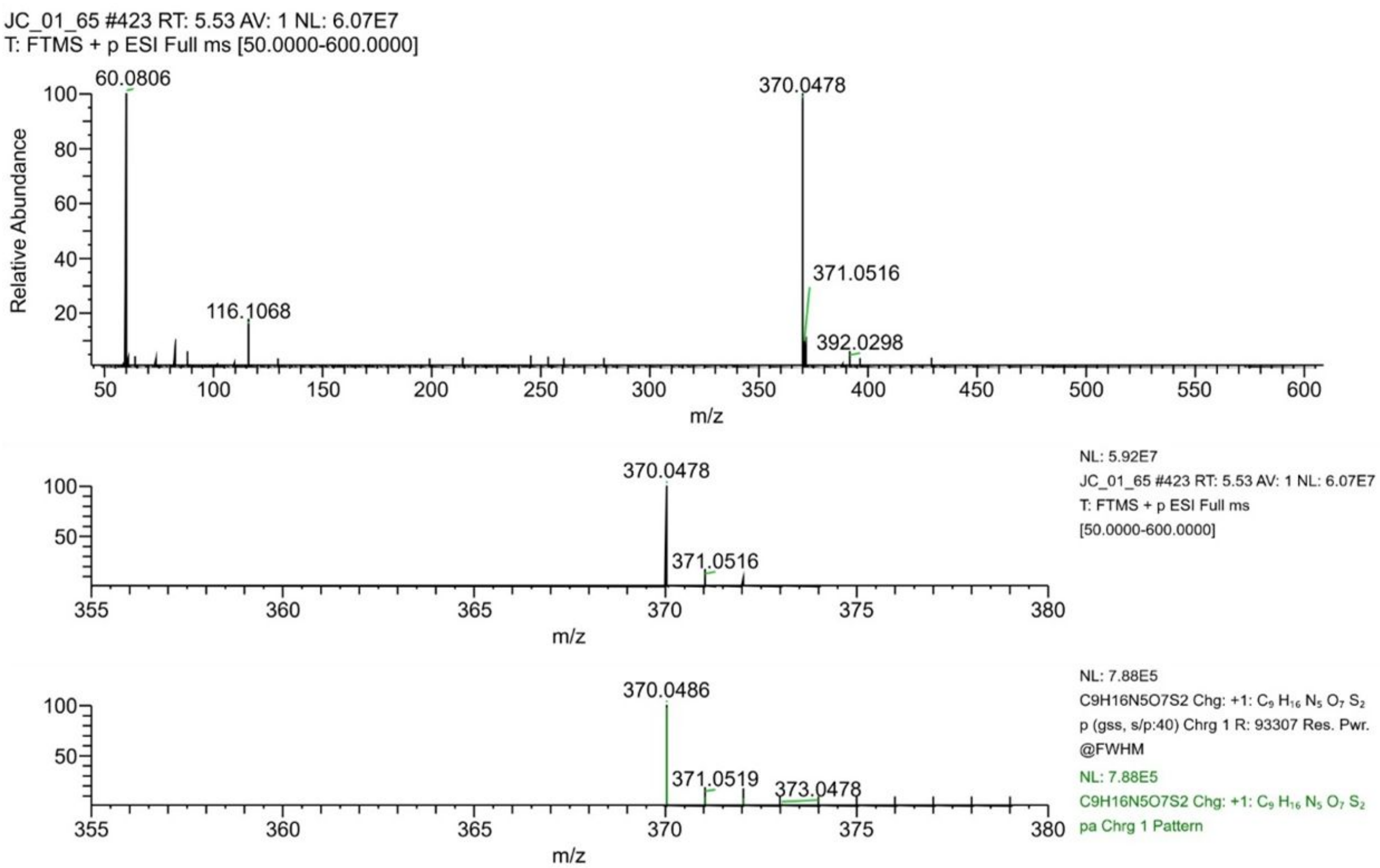
JC_01_65 #423 RT: 5.53 AV: 1 NL: 6.07E7
T: FTMS + p ESI Full ms [50.0000-600.0000]
60.0806
370.0478
371.0516
116.1068
392.0298
Relative Abundance
m/z
NL: 5.92E7
JC_01_65 #423 RT: 5.53 AV: 1 NL: 6.07E7
T: FTMS + p ESI Full ms
[50.0000-600.0000]
370.0486
371.0519
373.0478
NL: 7.88E5
C9H16N5O7S2 Chg: +1: C9 H16 N5 O7 S2
p (gss, s/p:40) Chrg 1 R: 93307 Res. Pwr.
@FWHM
NL: 7.88E5
C9H16N5O7S2 Chg: +1: C9 H16 N5 O7 S2
pa Chrg 1 Pattern

Figure S17. HRMS spectra of the AS standard (t_R = 5.52 min) in both negative and positive modes.

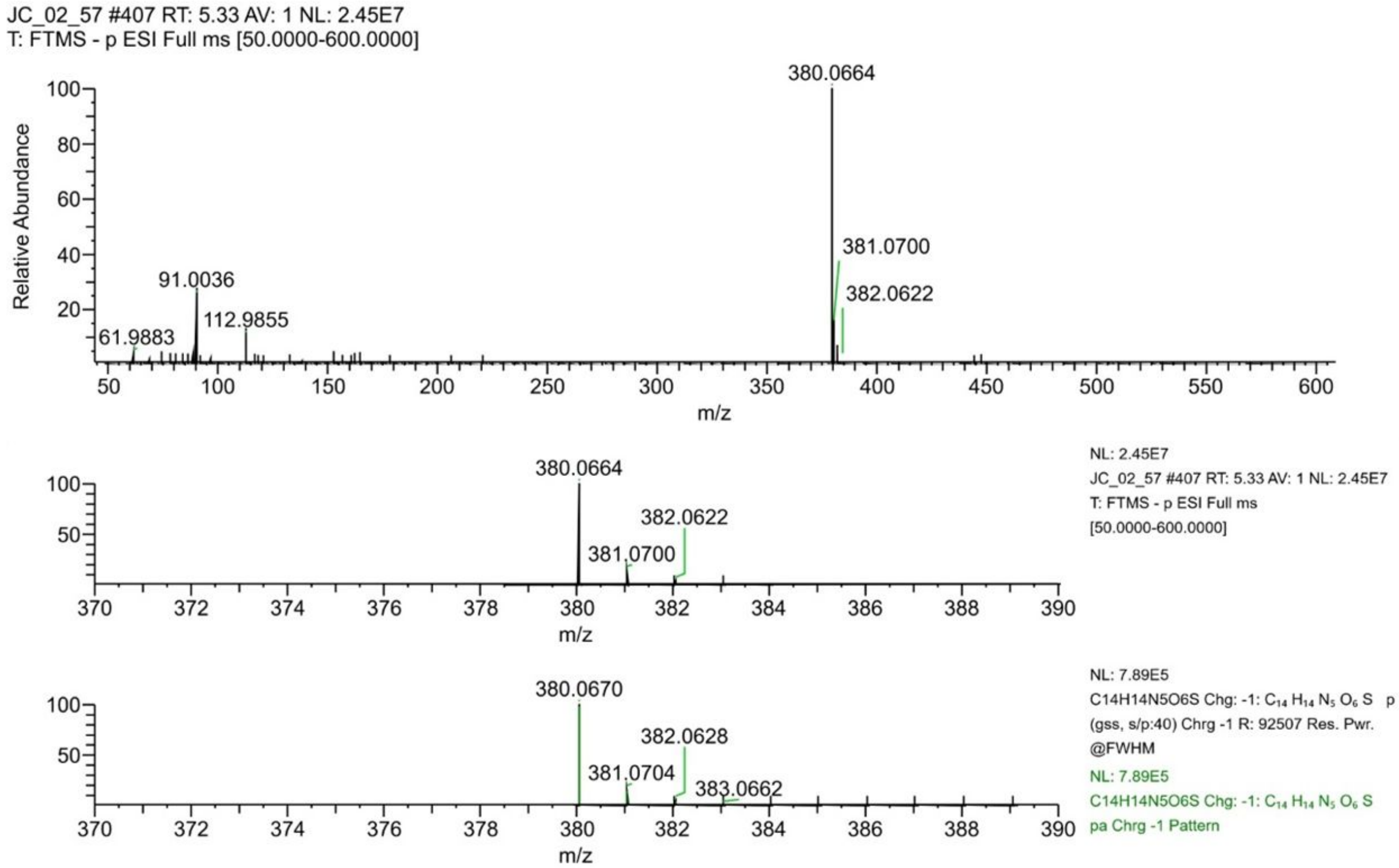
JC_02_57 #407 RT: 5.33 AV: 1 NL: 2.45E7
T: FTMS - p ESI Full ms [50.0000-600.0000]
Relative Abundance
m/z
380.0664
381.0700
382.0622
91.0036
112.9855
61.9883
NL: 2.45E7
JC_02_57 #407 RT: 5.33 AV: 1 NL: 2.45E7
T: FTMS - p ESI Full ms
[50.0000-600.0000]
NL: 7.89E5
C14H14N5O6S Chg: -1: C14 H14 N5 O6 S p
(gss, s/p:40) Chrg -1 R: 92507 Res. Pwr.
@FWHM
NL: 7.89E5
C14H14N5O6S Chg: -1: C14 H14 N5 O6 S
pa Chrg -1 Pattern
380.0670
382.0628
381.0704
383.0662

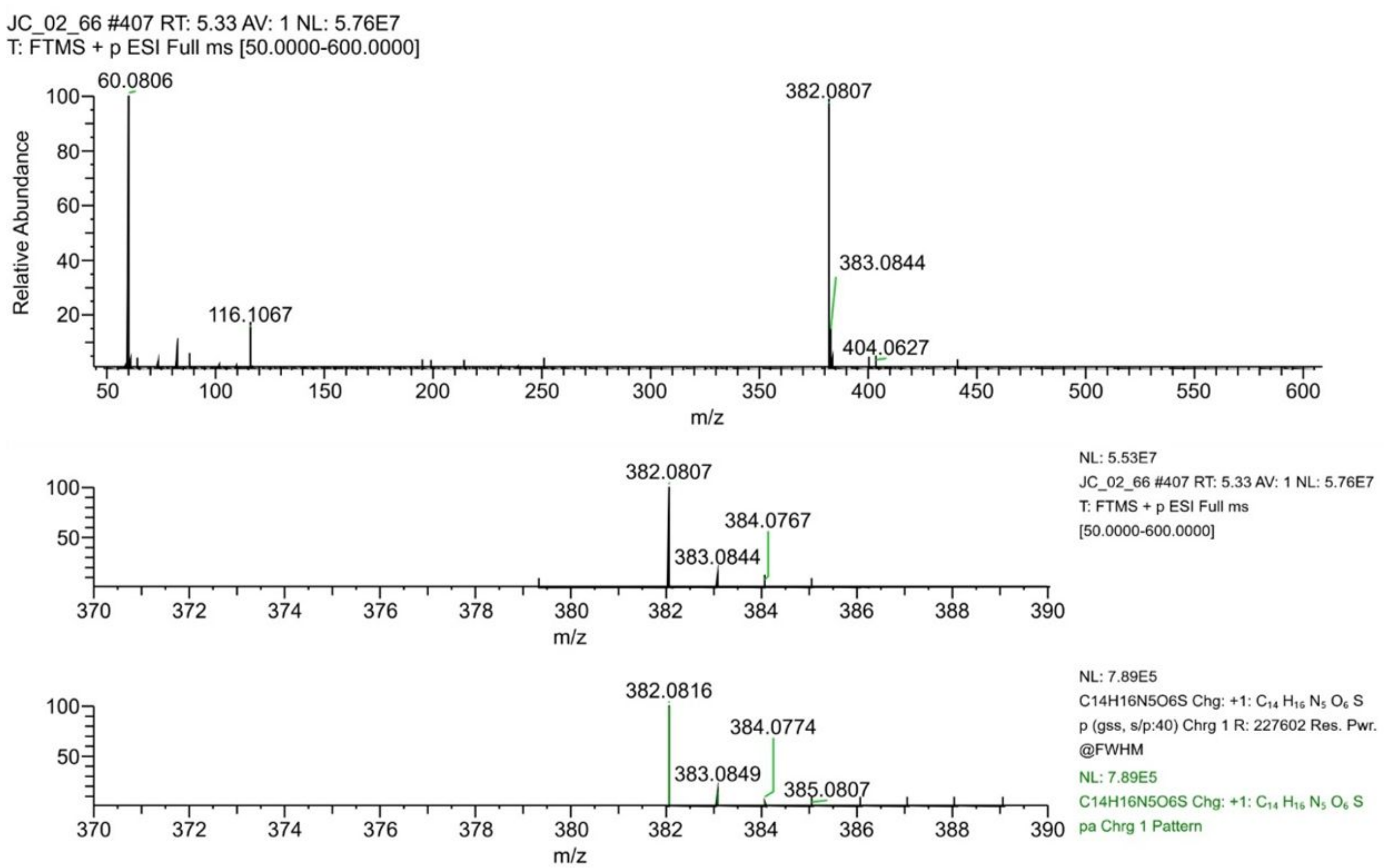
JC_02_66 #407 RT: 5.33 AV: 1 NL: 5.76E7
T: FTMS + p ESI Full ms [50.0000-600.0000]
Relative Abundance
m/z
60.0806
382.0807
383.0844
116.1067
404.0627
NL: 5.53E7
JC_02_66 #407 RT: 5.33 AV: 1 NL: 5.76E7
T: FTMS + p ESI Full ms
[50.0000-600.0000]
384.0767
NL: 7.89E5
C14H16N5O6S Chg: +1: C14 H16 N5 O6 S
p (gss, s/p:40) Chrg 1 R: 227602 Res. Pwr.
@FWHM
NL: 7.89E5
C14H16N5O6S Chg: +1: C14 H16 N5 O6 S
pa Chrg 1 Pattern
382.0816
384.0774
383.0849
385.0807

Figure S18. HRMS spectra of the MSM standard (t_R = 5.52 min) in both negative and positive modes.

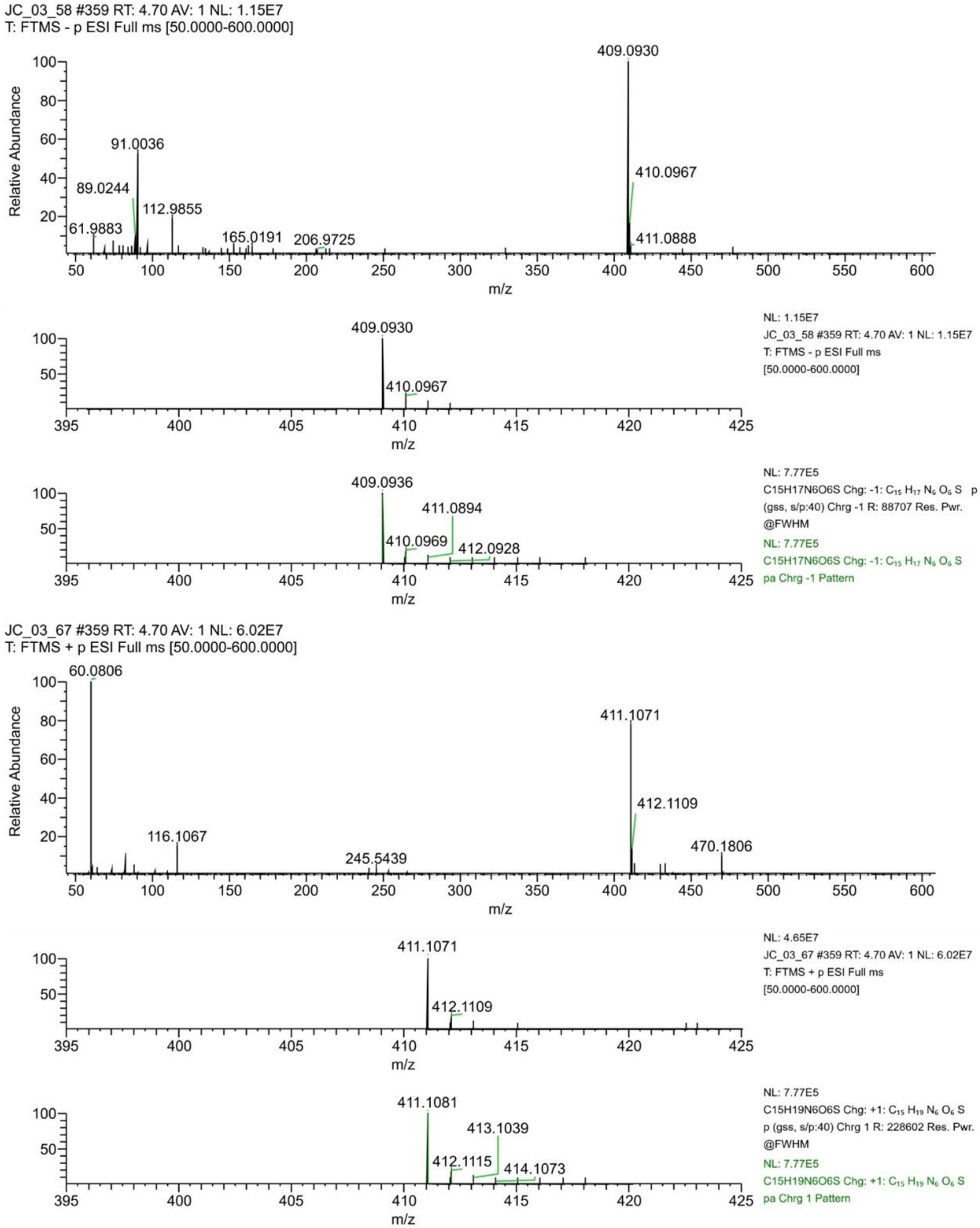

Figure S19. HRMS spectra of the NS standard (t_R = 5.52 min) in both negative and positive modes.

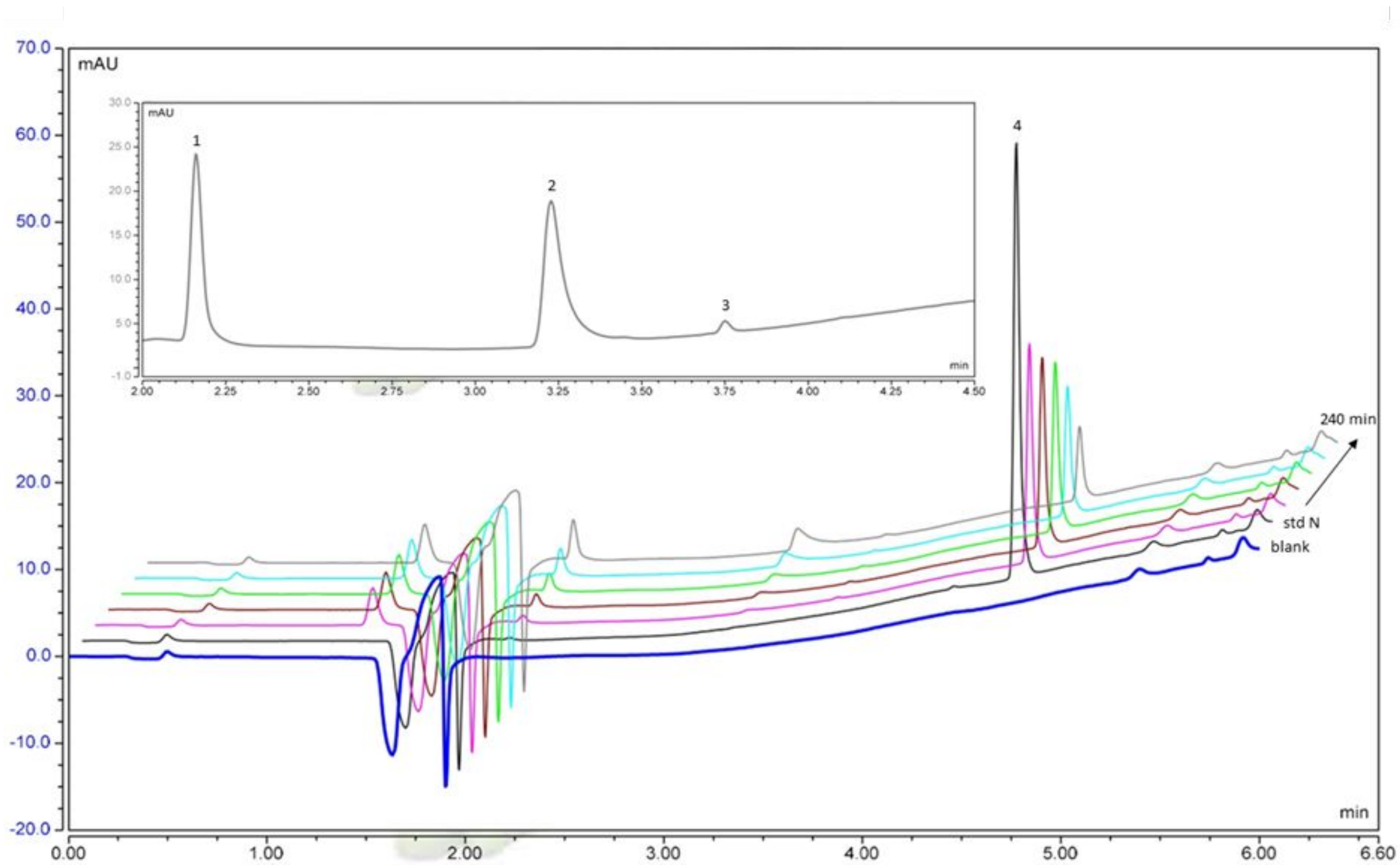

Figure S20. Time-resolved chromatograms of NS degradation on CePER sample (blank, std NS, and sample after 5, 30, 60, 120, and 240 min); 1 – N,N-dimethyl-2-sulfamoylnicotinamide ($C_8H_{11}N_3O_3S$; t_R = 2.17 min); 2 – 2-amino-4,6-dimethoxypyrimidine (ADMP; $C_6H_9N_3O_2$; t_R = 3.23 min); 3 – 2-[1-(4,6-dimethoxypyrimidin-2-yl)ureido]-N,N-dimethylnicotinamide ($C_{15}H_{18}N_6O_4$, t_R = 3.75 min), and 4 – NS (t_R = 4.70 min).

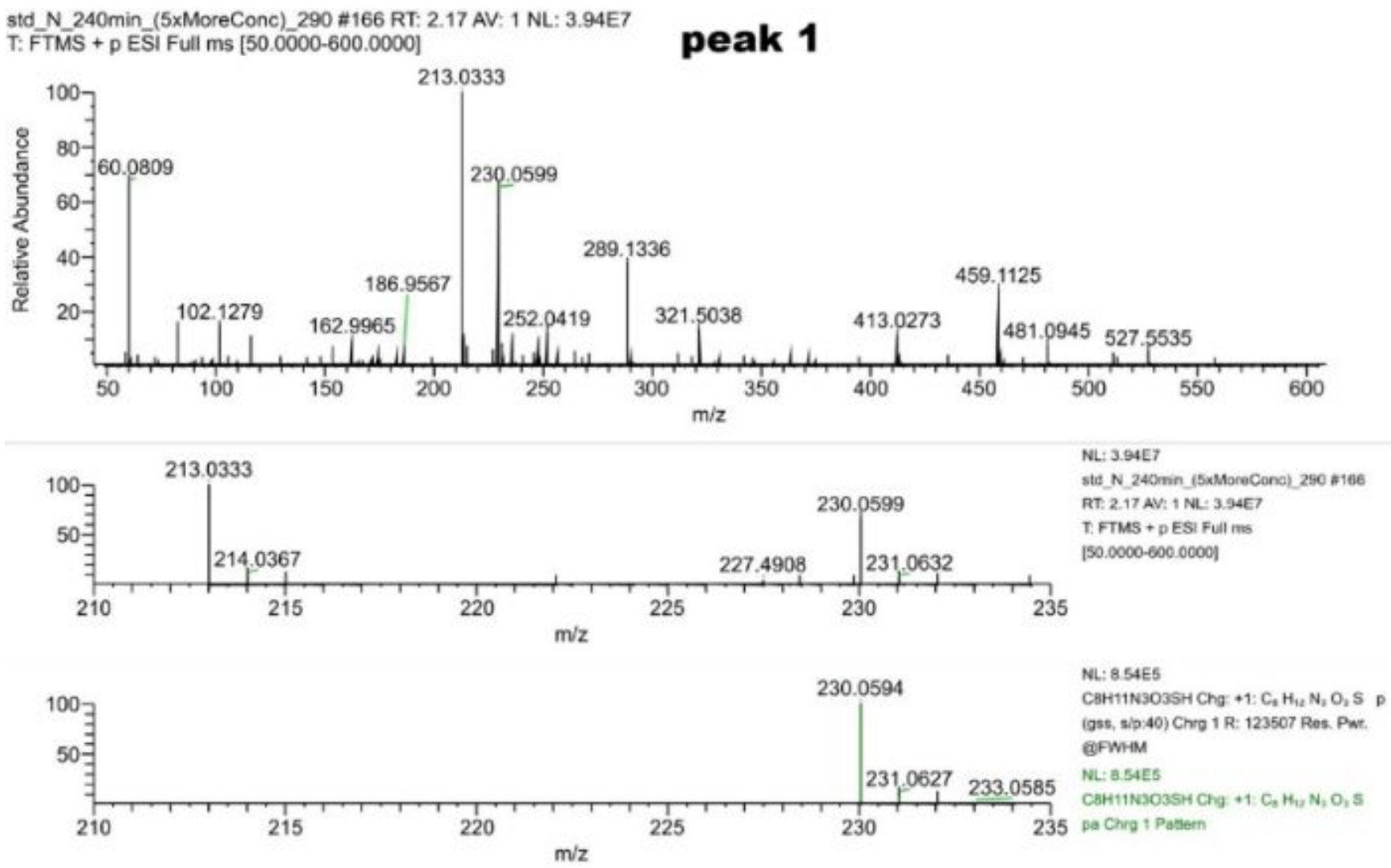
std_N_240min_(5xMoreConc)_290 #166 RT: 2.17 AV: 1 NL: 3.94E7
T: FTMS + p ESI Full ms [50.0000-600.0000]
peak 1
Relative Abundance
m/z
213.0333
60.0809
230.0599
289.1336
186.9567
459.1125
102.1279
162.9965
252.0419
321.5038
413.0273
481.0945
527.5535
214.0367
227.4908
231.0632
NL: 3.94E7
std_N_240min_(5xMoreConc)_290 #166
RT: 2.17 AV: 1 NL: 3.94E7
T: FTMS + p ESI Full ms
[50.0000-600.0000]
230.0594
231.0627
233.0585
NL: 8.54E5
C8H11N3O3SH Chg: +1
NL: 8.54E5
C8H11N3O3SH Chg: +1
pa Chrg 1 Pattern

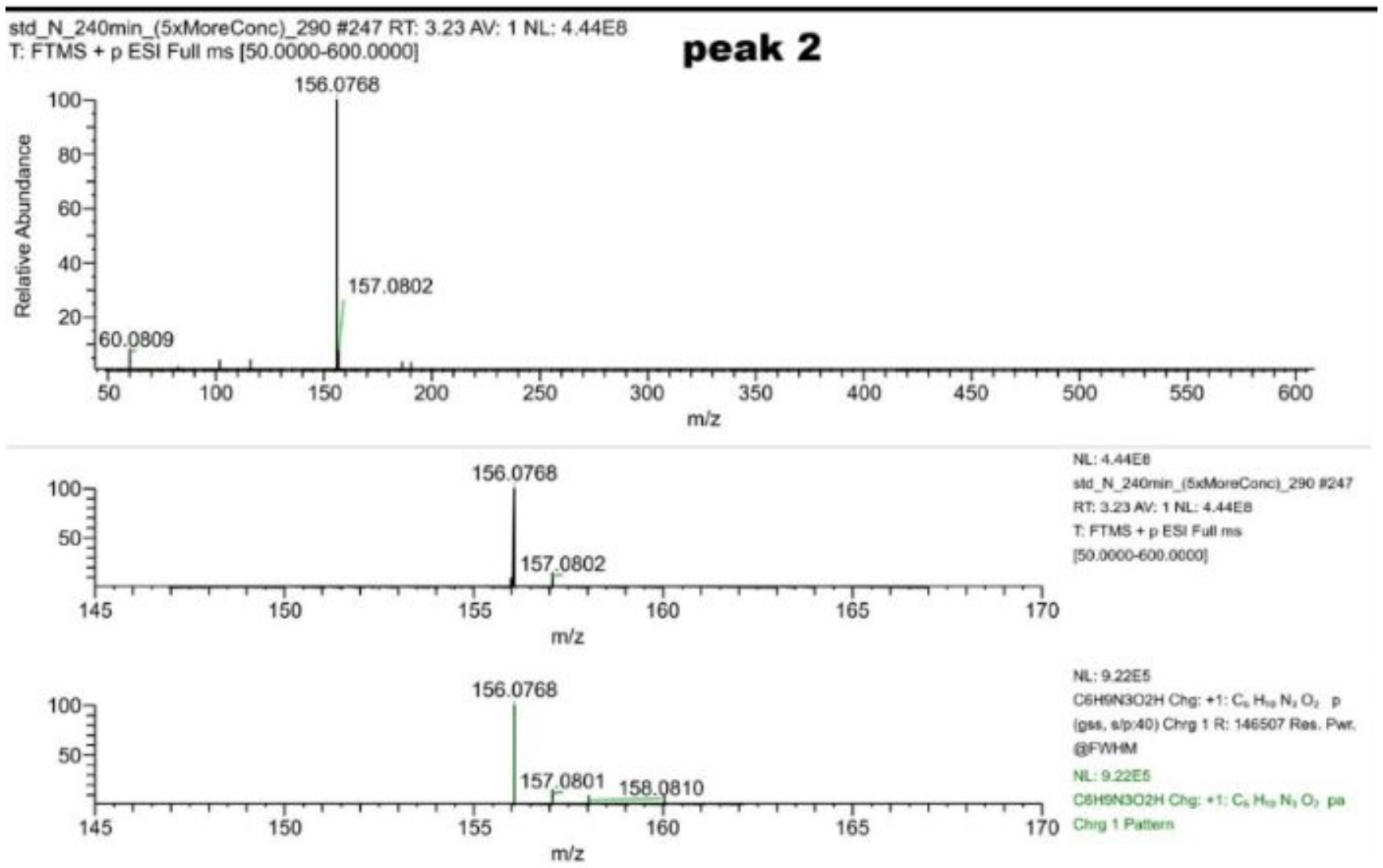
std_N_240min_(5xMoreConc)_290 #247 RT: 3.23 AV: 1 NL: 4.44E8
T: FTMS + p ESI Full ms [50.0000-600.0000]
peak 2
Relative Abundance
m/z
156.0768
157.0802
60.0809
NL: 4.44E8
std_N_240min_(5xMoreConc)_290 #247
RT: 3.23 AV: 1 NL: 4.44E8
T: FTMS + p ESI Full ms
[50.0000-600.0000]
157.0801
158.0810
NL: 9.22E5
C6H9N3O2H Chg: +1
NL: 9.22E5
C6H9N3O2H Chg: +1
Chrg 1 Pattern

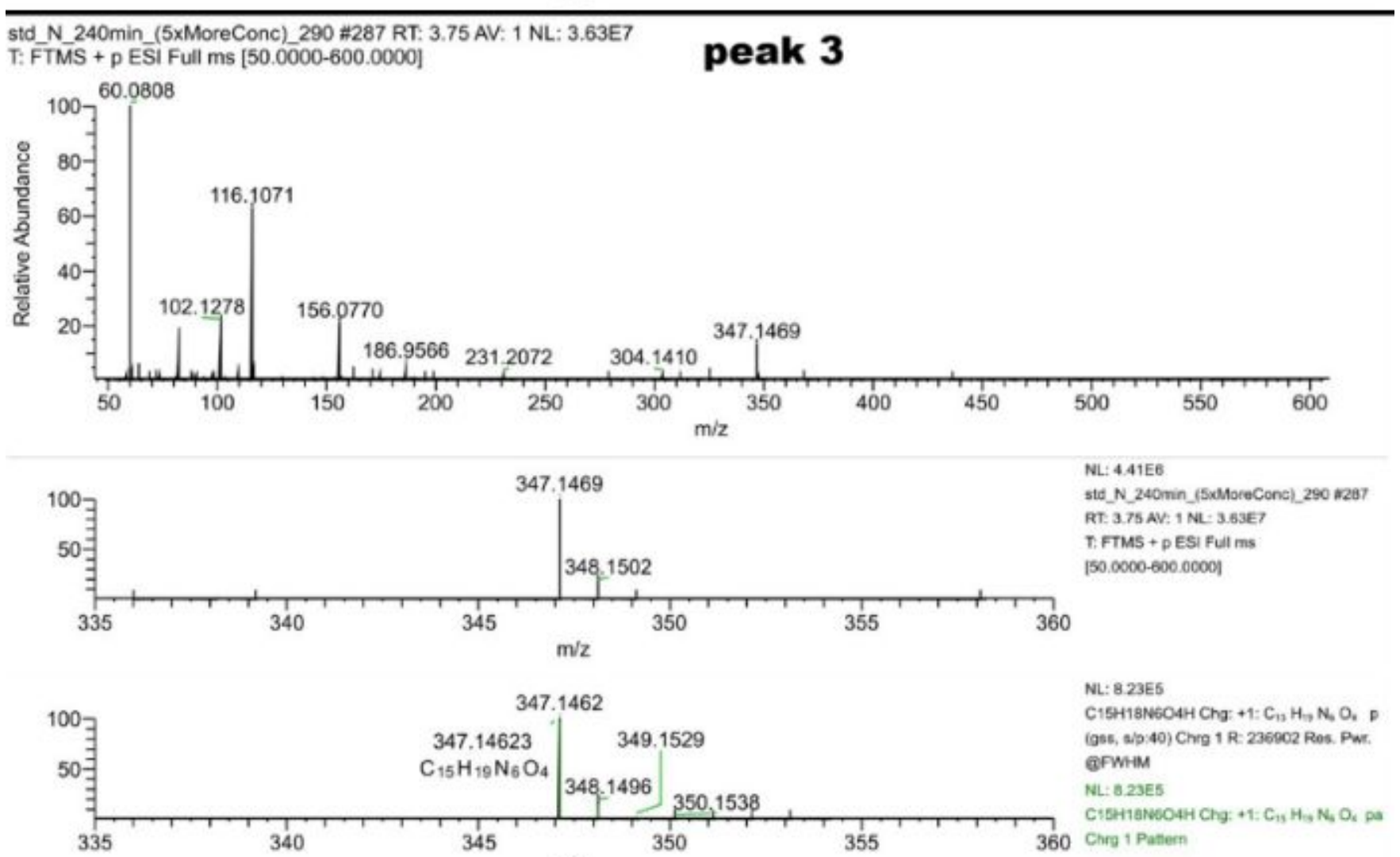
std_N_240min_(5xMoreConc)_290 #287 RT: 3.75 AV: 1 NL: 3.63E7
T: FTMS + p ESI Full ms [50.0000-600.0000]
peak 3
Relative Abundance
m/z
60.0808
116.1071
102.1278
156.0770
186.9566
231.2072
304.1410
347.1469
348.1502
NL: 4.41E6
std_N_240min_(5xMoreConc)_290 #287
RT: 3.75 AV: 1 NL: 3.63E7
T: FTMS + p ESI Full ms
[50.0000-600.0000]
347.1462
347.14623
C15H19N6O4
349.1529
348.1496
350.1538
NL: 8.23E5
C15H18N6O4H Chg: +1
NL: 8.23E5
C15H18N6O4H Chg: +1
Chrg 1 Pattern

Figure S21. HRMS spectrum of compounds identified as DPs of NS: peak 1: N,N-dimethyl-2-sulfamoylnicotinamide ($C_8H_{11}N_3O_3S$) detected at t_R = 2.17 min, peak 2: 2-amino-4,6-dimethoxypyrimidine (ADMP; $C_6H_9N_3O_2$) detected at t_R = 3.23 min and peak 3: 2-[1-(4,6-dimethoxypyrimidin-2-yl)ureido]-N,N-dimethsylnicotinamide ($C_{15}H_{19}N_6O_4$) detected at t_R = 3.75 min.

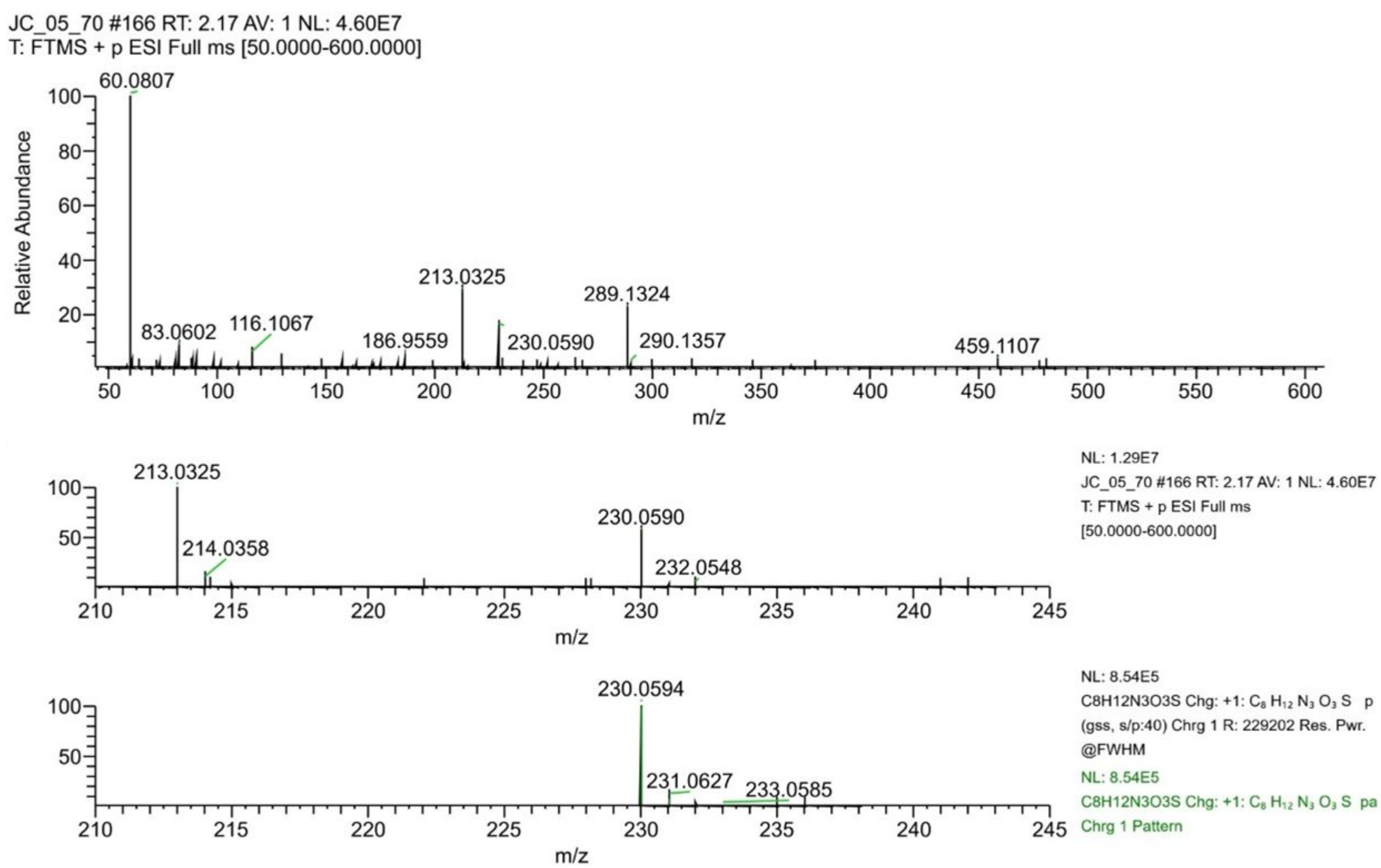

Figure S22. HRMS spectra of the N,N-dimethyl-2-sulfamoylnicotinamide standard (t_R = 2.17 min).

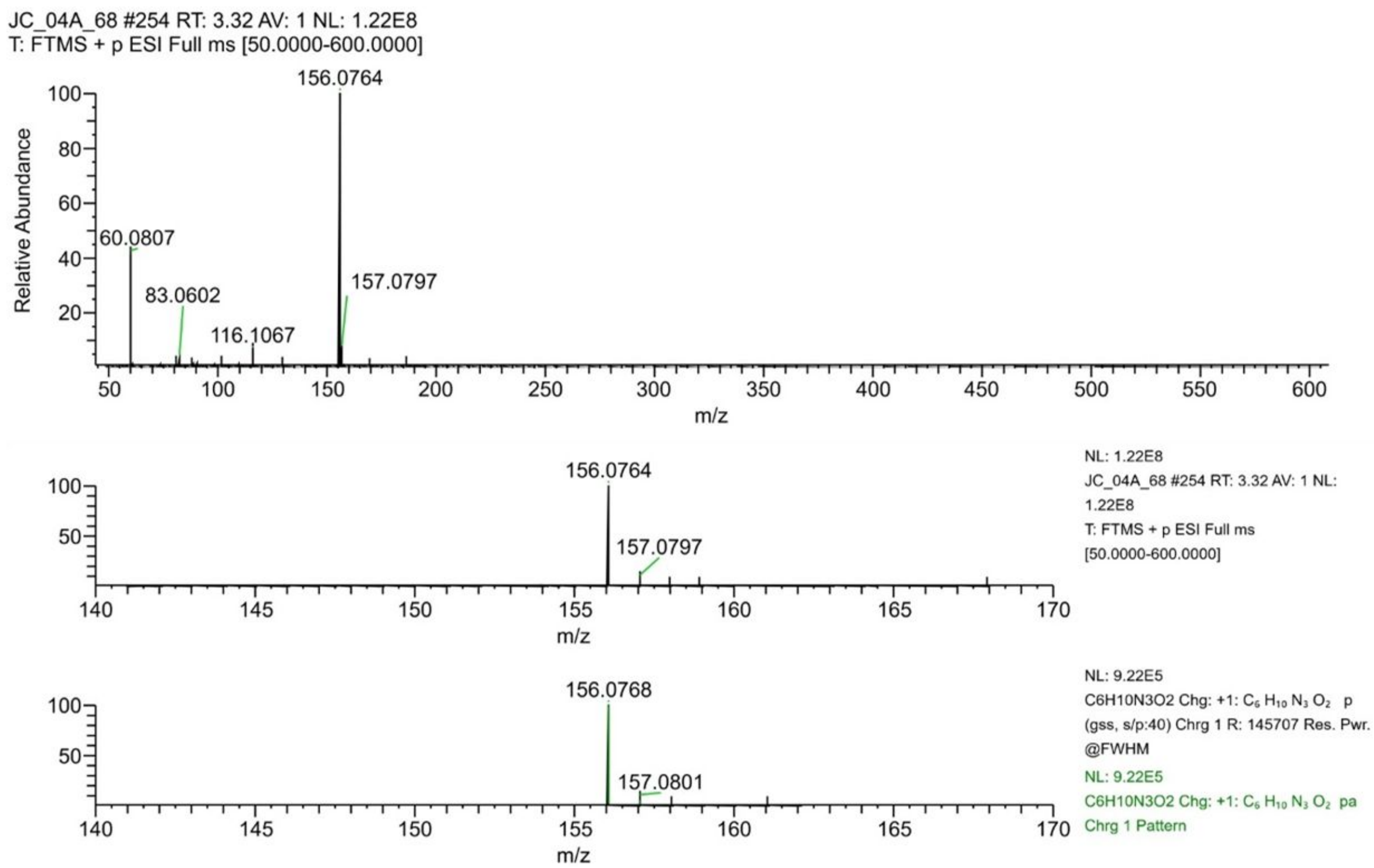

Figure S23. HRMS spectra of 2-amino-4,6-dimethoxypyrimidine (ADMP) standard.

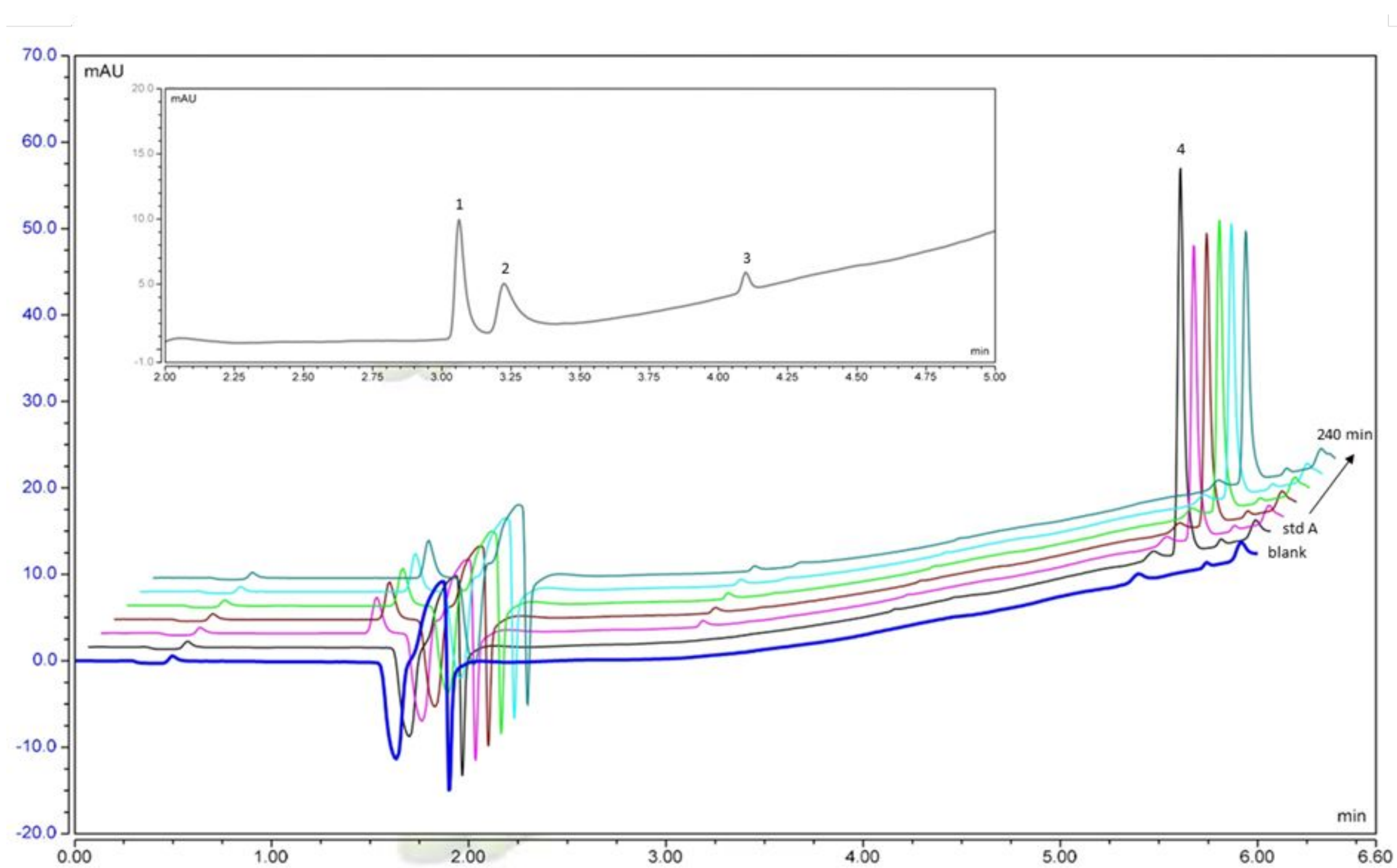

Figure S24. Time-resolved chromatograms of AS degradation on CePER sample (blank, AS standard, and samples after 5, 30, 60, 120, and 240 min): 1 – [(4,6-dimethoxypyrimidin-2-yl)carbamoyl]sulfamic acid ($C_7H_{10}N_4O_6S$; BCS-AW41401; t_R = 3.06 min); 2 – 2-amino-4,6-dimethoxypyrimidine (ADMP; $C_6H_9N_3O_2$, t_R = 3.23 min); 3 – 4,6-dimethoxypyrimidin-2-ylurea ($C_7H_{10}N_4O_3$, t_R = 4.11 min), and 4 – AS (t_R = 5.53 min).

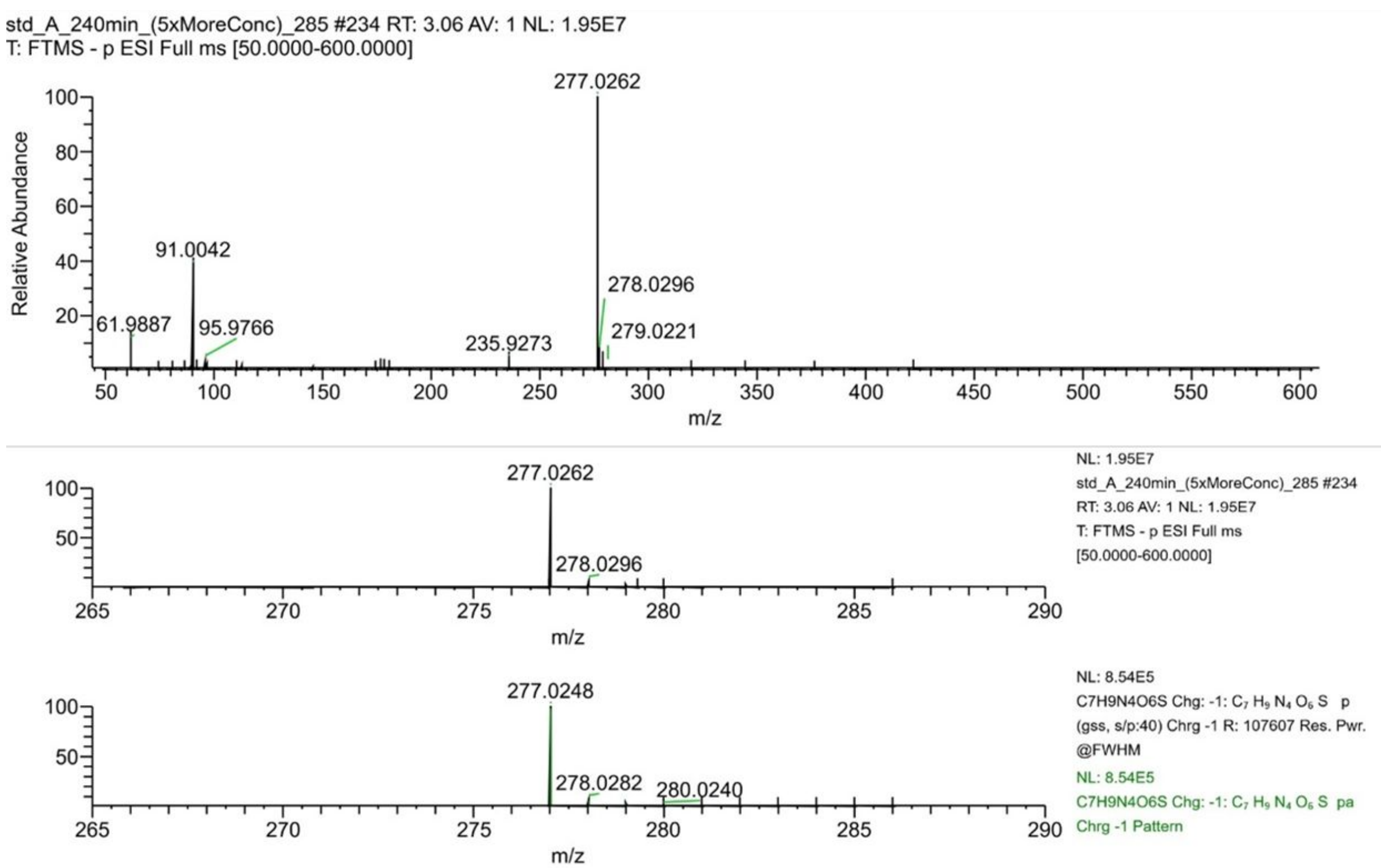

Figure S25. HRMS spectrum of [(4,6-dimethoxypyrimidin-2-yl)carbamoyl]sulfamic acid ($C_7H_9N_4O_6S$; BCS-AW41401) detected at t_R = 3.06 min, identified as DP of AS (peak 1).

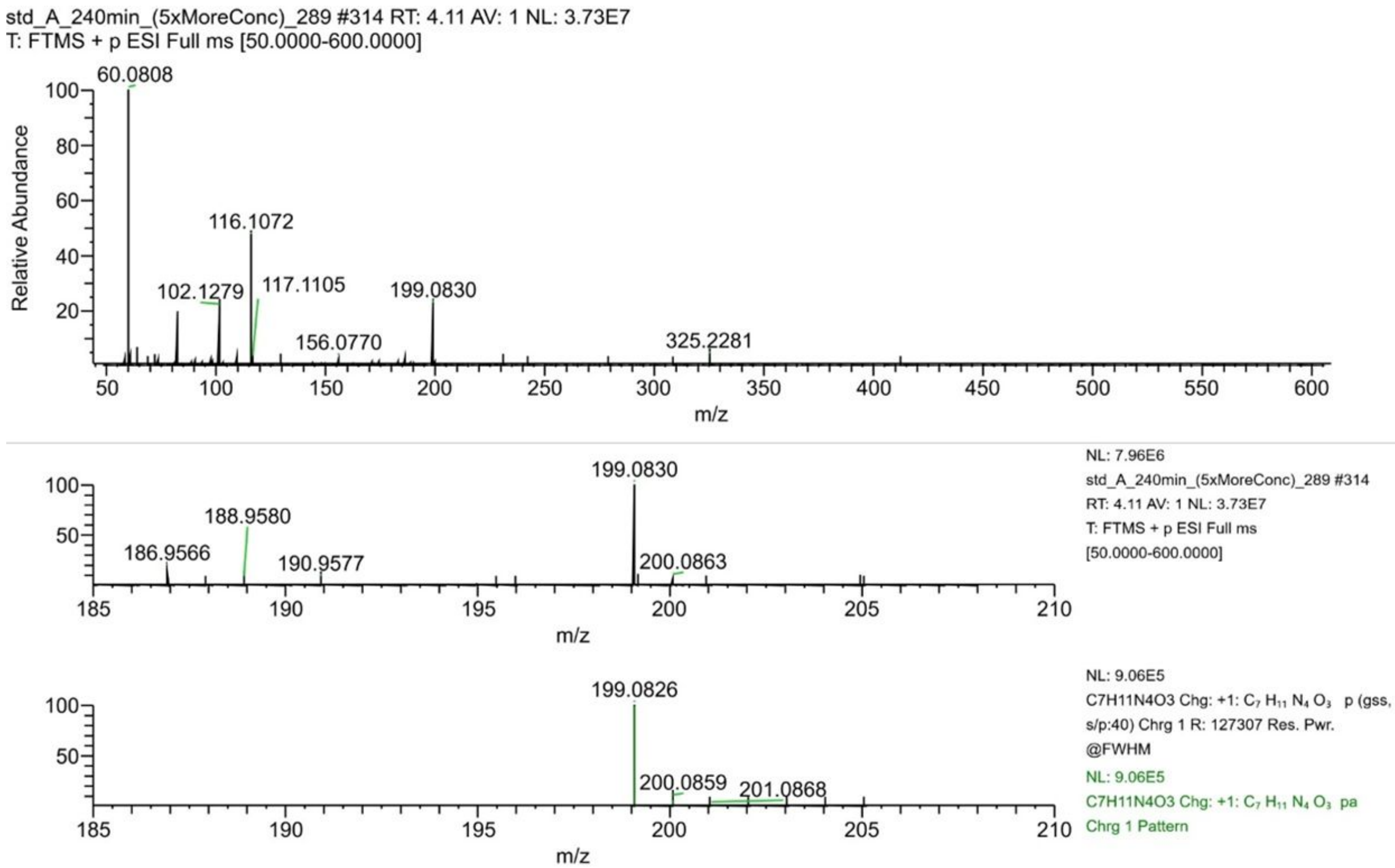

Figure S26. HRMS spectrum of 4,6-dimethoxypyrimidin-2-ylurea($C_7H_{11}N_4O_3$) detected at t_R = 4.11 min, identified as DP of AS (peak 3).

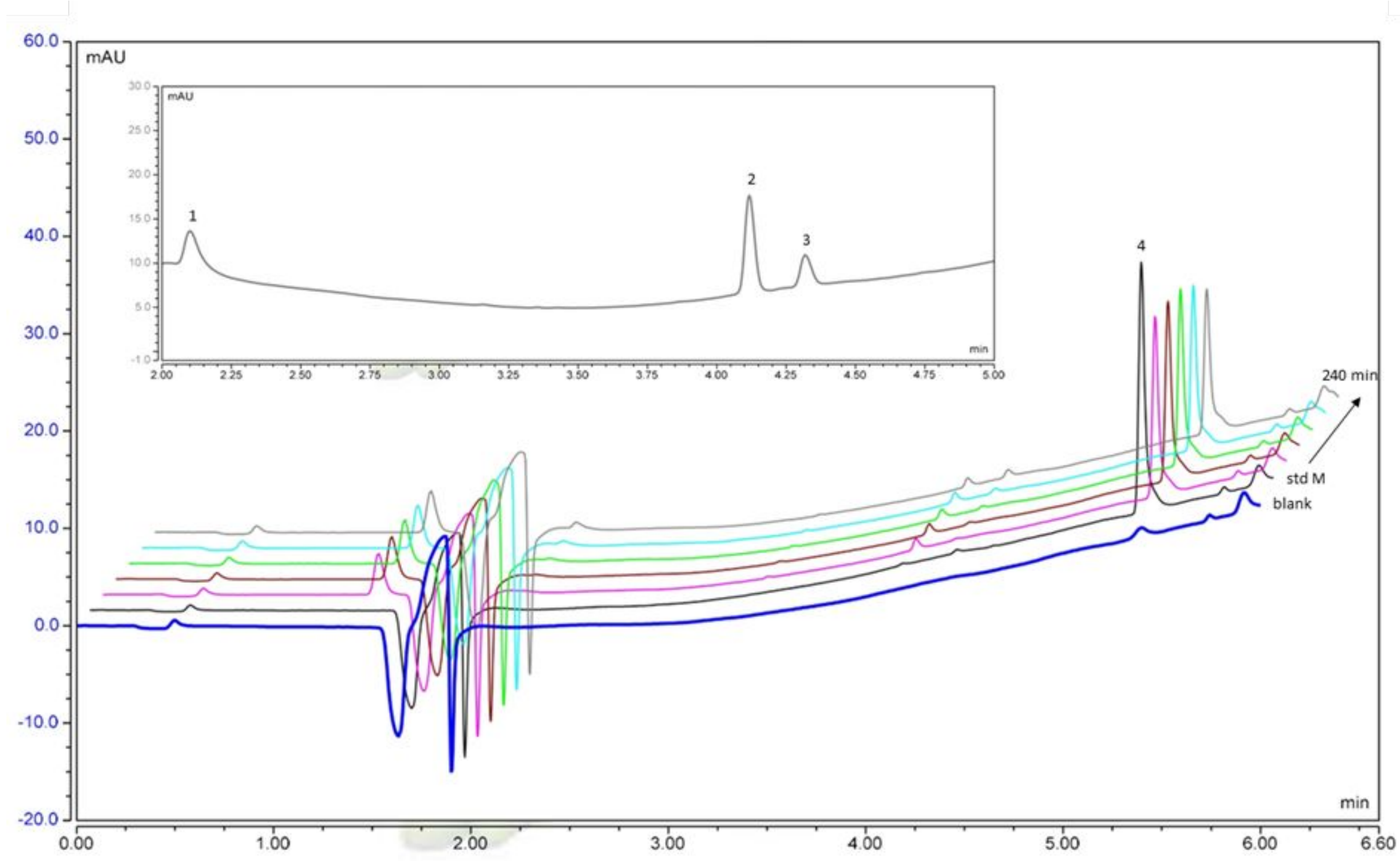

Figure S27. Time-resolved chromatograms of MSM degradation on CePER sample (blank, std MSM, and sample after 5, 30, 60, 120, and 240 min): 1 – 2-amino-4-methoxy-6-methyl-1,3,5-triazine (C5H8N4O; t_R = 2.11 min), 2 – MSM without –CH_3 $C_{13}H_{14}N_5O_6S$ (t_R = 4.12 min), 3 – methyl 2-(aminosulfonyl)benzoate ($C_8H_9NO_4S$; t_R = 4.33 min), and 4 – MSM (t_R = 5.33 min).

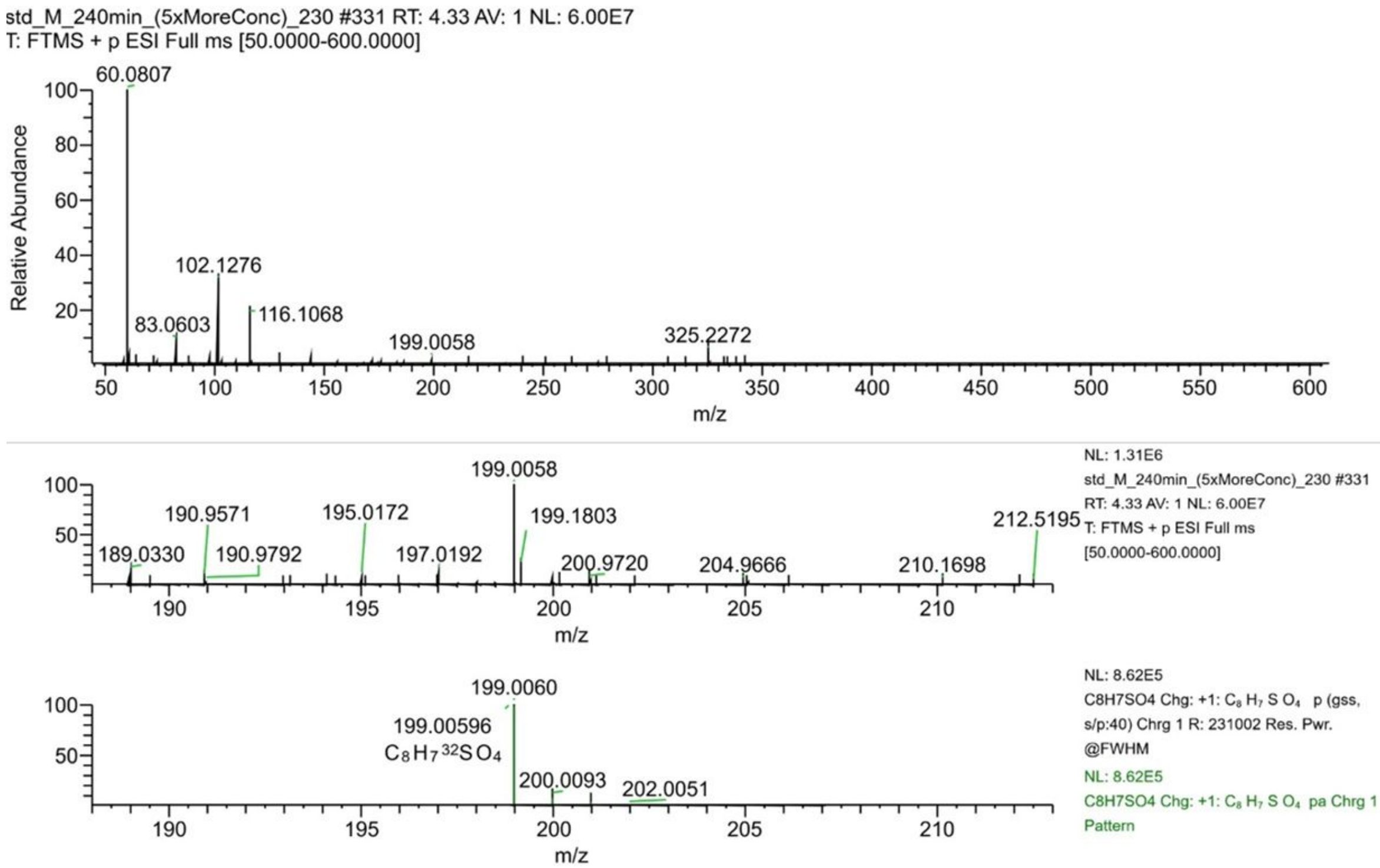

Figure S28. HRMS spectra of methyl 2-(aminosulfonyl)benzoate ($C_8H_{10}NO_4S$) detected at t_R = 4.33 min, identified as the main DP of metsulfuron-methyl (peak 3).

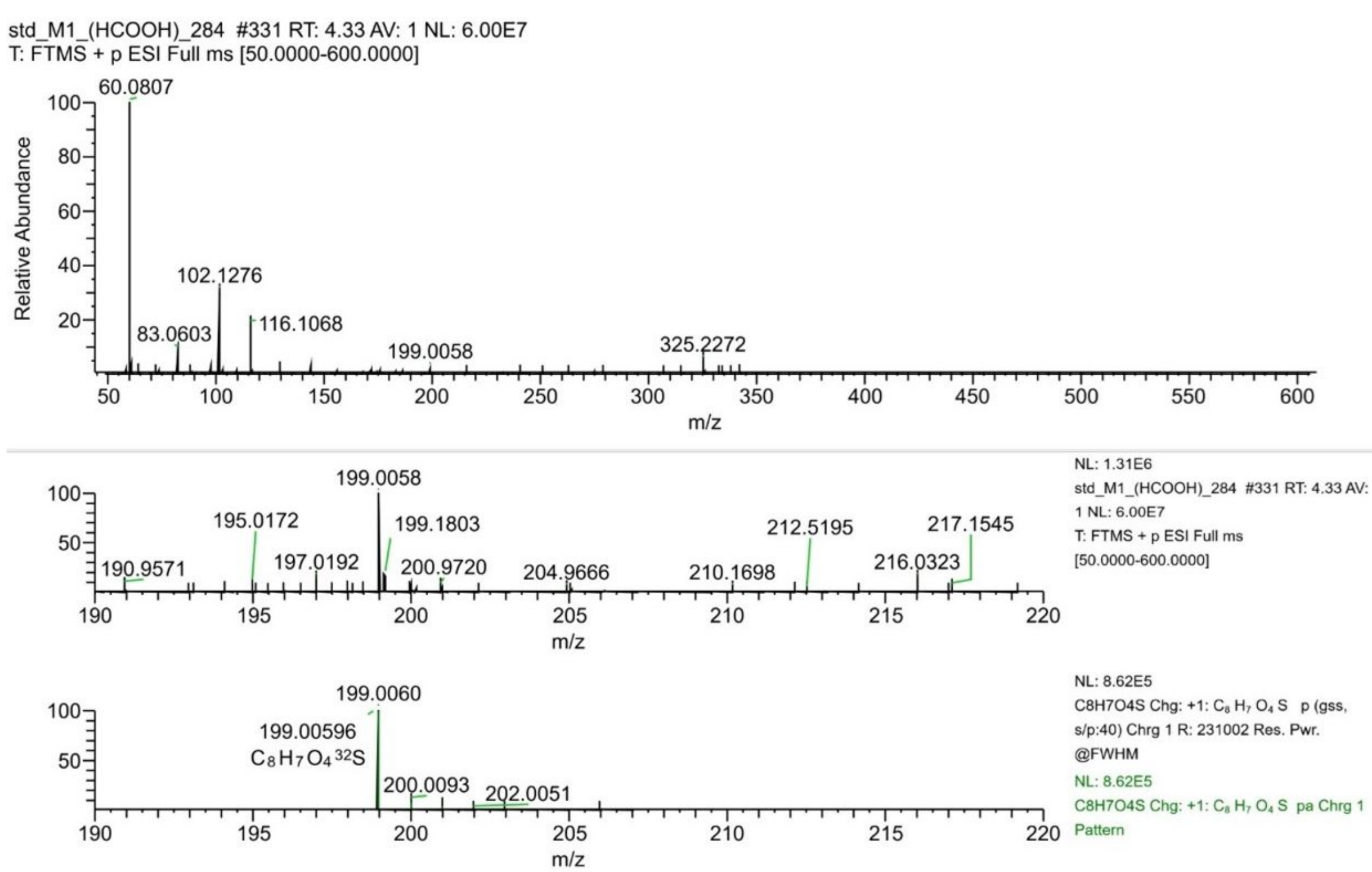

Figure S29. HRMS spectra of methyl-2-(aminosulfonyl)benzoate standard (t_R = 4.33 min).

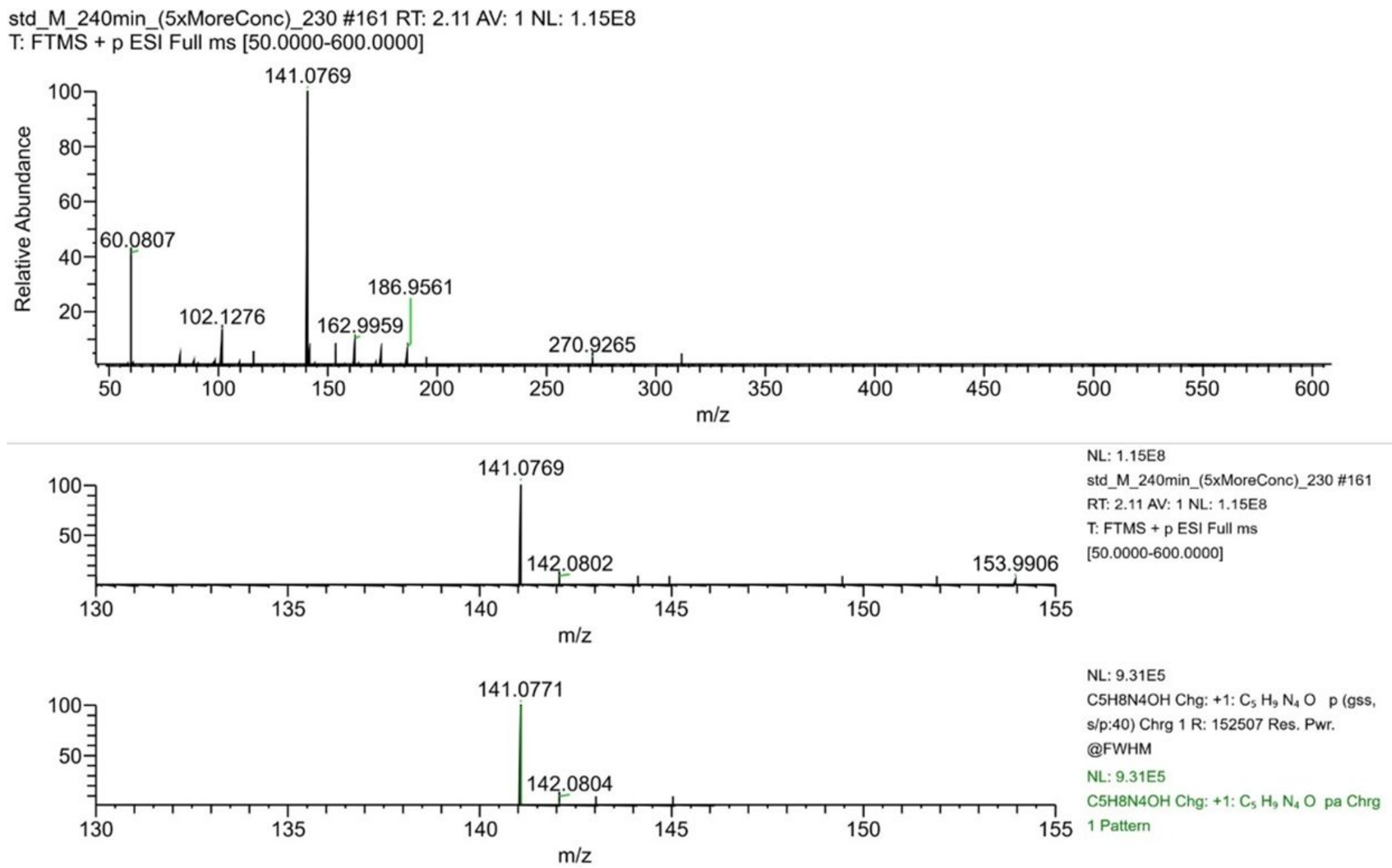

Figure S30. HRMS spectra 2-amino-4-methoxy-6-methyl-1,3,5-triazine ($C_5H_8N_4O$) detected at t_R = 2.11 min, identified as the main DP of MS (peak 1).

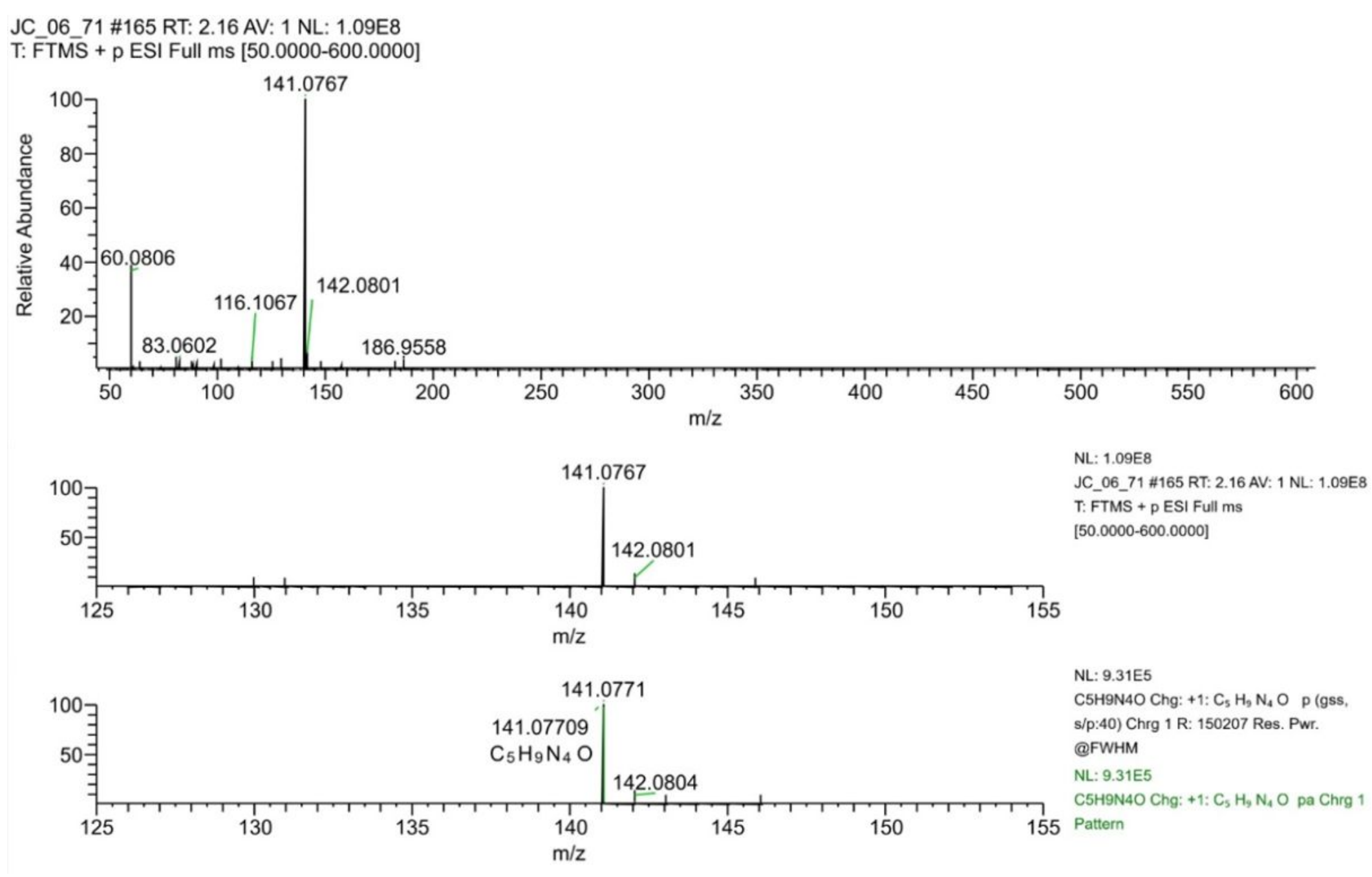

Figure S31. HRMS spectra of the 2-amino-4-methoxy-6-methyl-1,3,5-triazine standard (t_R = 2.11 min).

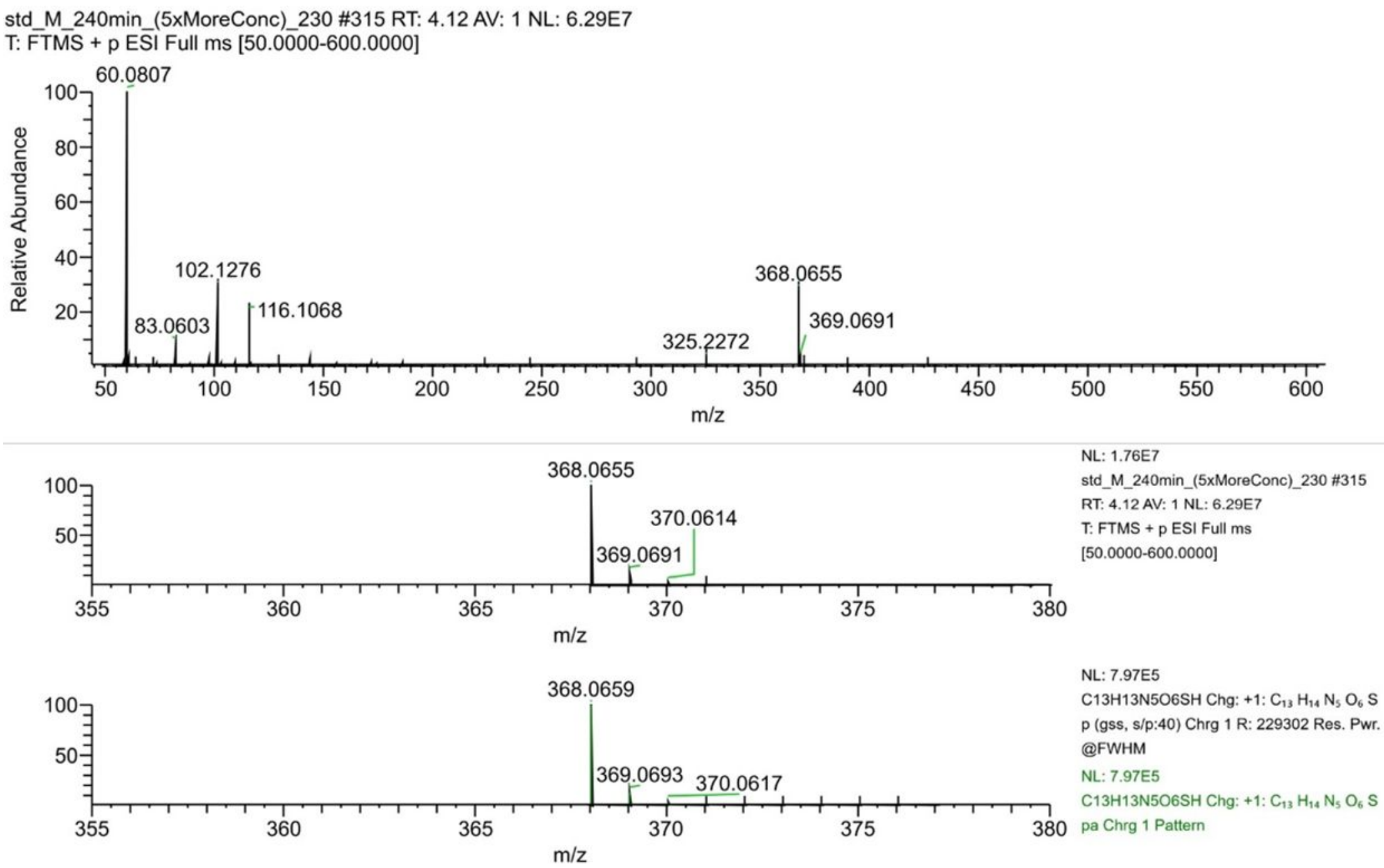

Figure S32. HRMS spectra of MSM lacking a –CH_3 substituent ($C_{13}H_{13}N_5O_6SH$) detected at t_R = 4.12 min, identified as the DP of MSM (peak 2).

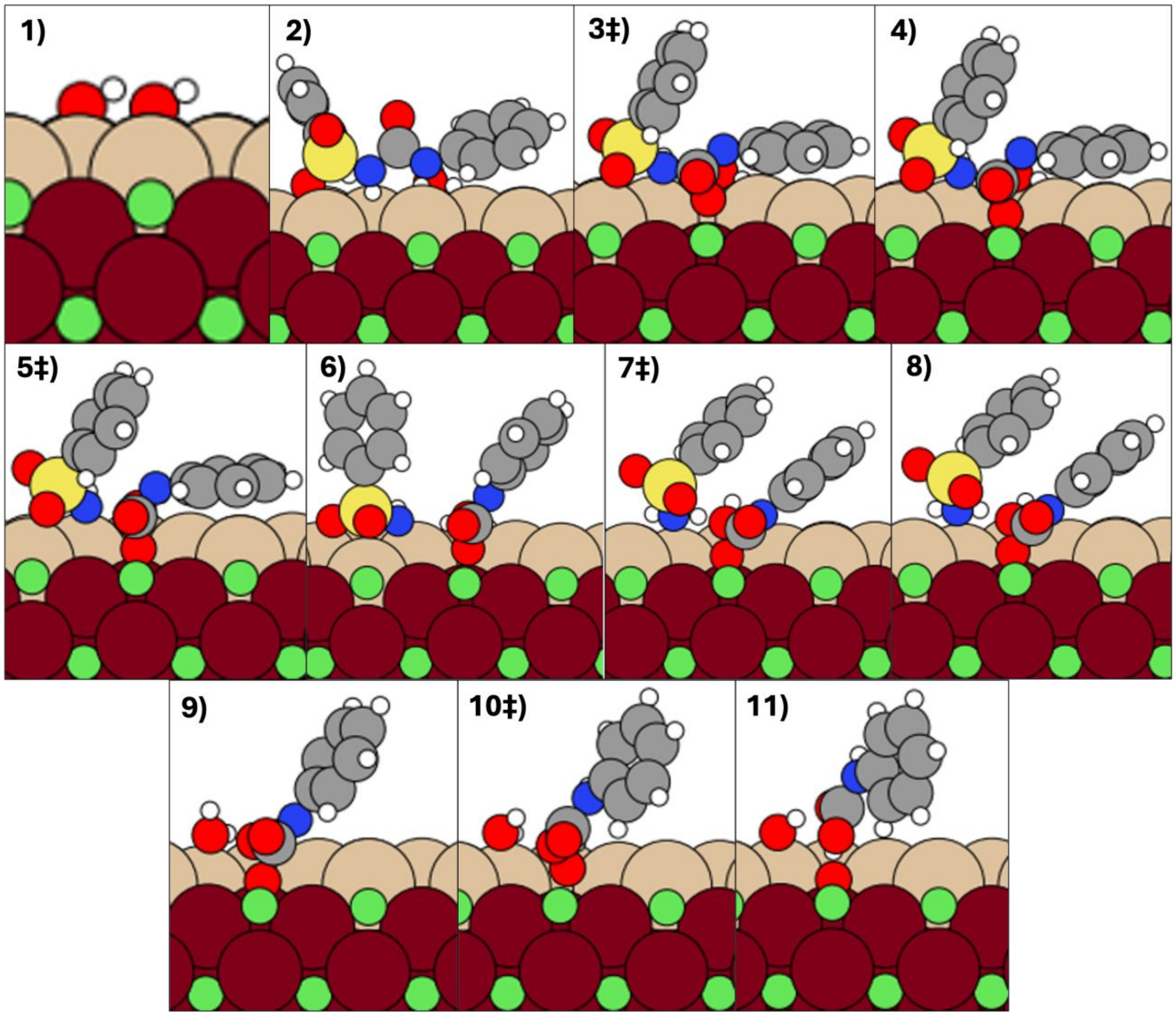

Figure S33. Side view of the stable intermediate and transition (indicated by ‡) states in the elementary steps in the proposed reaction pathway. Numbering of states corresponds to that in Figure 9. The states shown are (1) water adsorption, (2) 1B3PhU adsorption, (3‡) TS of nucleophilic attack by O_{latt}, (4) TI, (5‡) TS of N_S–C bond scission, (6) amino + carbamyl, (7‡) TS of hydrogen transfer from H_2O, (8) amine + OH + carbamyl, (9) OH + carbamyl, (10‡) TS of OH attack, and (11) carbamate + H. Color code: green=Ce; light and dark brown=top- and lower-layer O_{latt}; red=O in molecules; black=C; blue=N; and white=H. O_{latt} bonded to C or H atoms are considered part of molecules.
